# Comparative Proteomic Analyses Between Biofilm-Forming and Non-biofilm-Forming Strains of *C*o*rynebacterium pseudotuberculosis* Isolated From Goats

**DOI:** 10.3389/fvets.2021.614011

**Published:** 2021-02-16

**Authors:** Maria Conceição Aquino de Sá, Wanderson Marques da Silva, Carla Catarine Santos Rodrigues, Cristiana Perdigão Rezende, Silvana Beutinger Marchioro, José Tadeu Raynal Rocha Filho, Thiago de Jesus Sousa, Helinando Pequeno de Oliveira, Mateus Matiuzzi da Costa, Henrique César Pereira Figueiredo, Ricardo Dias Portela, Thiago Luiz de Paula Castro, Vasco Azevedo, Nubia Seyffert, Roberto Meyer

**Affiliations:** ^1^Institute of Health Sciences, Federal University of Bahia, Salvador, Brazil; ^2^Instituto de Agrobiotecnología y Biologia Molecular Instituto Nacional de Tecnología Agropecuária/Consejo Nacional de Investigaciones Científicas y Técnicas (IABIMO-INTA/CONICET), Buenos Aires, Argentina; ^3^Veterinary School, Federal University of Minas Gerais, Belo Horizonte, Brazil; ^4^Institute of Biological Sciences, Federal University of Minas Gerais, Belo Horizonte, Brazil; ^5^Institute of Materials Science, Federal University of São Francisco Valley, Juazeiro, Brazil; ^6^Department of Zootechnics, Federal University of São Francisco Valley, Petrolina, Brazil

**Keywords:** bacterial biofilm, caseous lymphadenitis, proteomics, small ruminants, virulence factors

## Abstract

Caseous lymphadenitis (CLA) is a chronic disease that affects small ruminants and causes economic losses in the associated breeding system. The causative agent of CLA is *Corynebacterium pseudotuberculosis*, a Gram-positive bacterium that exhibits tropism for external and internal lymph nodes and induces abscess formation in the host. Bacterial communities often produce a biofilm matrix that serves various functions, including protection against hostile environmental conditions, antibiotics, and the host immune response. Although biofilm formation has been reported for *C. pseudotuberculosis*, not all strains demonstrate this property in culture. In this work, we report the first comparative proteomic analysis of one biofilm-forming (CAPJ4) and one biofilm-non-forming strain (CAP3W) of *C. pseudotuberculosis* isolated from goats. Bacterial whole cell protein extracts were obtained for mass spectrometry analyses. Using LC-MS/MS, our studies reveal three and four proteins exclusively found in the CAPJ4 and CAP3W proteome, respectively. In addition, label-free quantitative analysis identified 40 proteins showing at-least 2-fold higher values in CAPJ4 compared CAP3W proteome Notably, CAPJ4 differentially synthesized the penicillin-binding protein, which participates in the formation of peptidoglycans. CAPJ4 also exhibited upregulation of N-acetylmuramoyl-L-alanine amidase and galactose-1-phosphate uridylyltransferase, which are involved in biofilm formation and exopolysaccharide biosynthesis. Here, we demonstrate that biofilm formation in *C. pseudotuberculosis* is likely associated with specific proteins, some of which were previously shown to be associated with virulence and biofilm formation in other organisms. Our findings may drive studies related to the bacterial mechanisms involved in the biofilm formation, in addition to providing targets for the treatment of CLA.

## Introduction

*Corynebacterium pseudotuberculosis* is the etiologic agent of caseous lymphadenitis (CLA), a disease that primarily affects goats and sheep (1). Infection in humans by this pathogen, has also been reported (2, 3). This disease can be found worldwide; however, it is concentrated in regions that rely heavily on the commercialization of products derived from small ruminants (4). CLA presents with symptoms characterized by the development of abscesses in visceral and superficial lymph nodes of animals (5).

Bacterial biofilms are composed of microbial communities embedded in an extracellular polymeric matrix and can attach to various surfaces (6). Pathogens may form biofilms in animals, which can consequently lead to chronic diseases characterized by the presence of abscesses (7).

The biofilm life cycle includes attachment, growth, and detachment. During the detachment stage, the surface bacteria begin to shed from the biofilm with the objective of colonizing other areas; however, the bacteria residing in the inner layers remain in the biofilm and continue to grow (8). This process of protecting the bacterial community may facilitate the development of abscesses because of the associated host immune response (9). Moreover, accumulation of purulent material and encapsulation may interfere with the capacity of antibiotics to penetrate the abscess and destroy the pathogen (10). Thus, the bacterial capacity to grow in biofilms is a consequence of a survival strategy adopted by many species in hostile environments (11).

The formation of biofilms by *C. pseudotuberculosis* has been previously reported (6, 12, 13), and bacteria in planktonic stages have been shown to be more susceptible to antibiotic action than those in the biofilm stage (7). Furthermore, when *C. pseudotuberculosis* is released from animals, it can persist in the environment for a long period (14). However, the dynamics of biofilm formation in *C. pseudotuberculosis* remain poorly understood and require an in-depth identification of genes and their respective products responsible for this characteristic.

Proteomics has been widely applied to help understand the dynamic functional state of a genome at the protein level and can provide information about the proteins that might be responsible for the development of a specific observed phenotype (15). Since pathogenic bacterial strains exhibit different phenotypes, comparative proteomic analysis between bacterial pathogens has contributed to the identification of proteins related to virulence factors, antibiotic or chemical resistance, physiological processes, and adaptation to specific environmental conditions (15, 16).

Various studies have shown the use of proteomics in the study of bacterial biofilm both Gram-positive and Gram-negative bacterial strains, allowing for the identification of bacterial proteins and functional groups that might be involved in their biofilm formation. Proteins identified through this approach, represent potential targets for antibiotics for reducing the formation of the bacterial biofilm (17).

Regarding *C. pseudotuberculosis*, comparative proteomic studies have expanded our knowledge on specific factors related to its pathophysiology (18–21). In this study, we applied label-free proteomic analysis to evaluate the differences between the proteome of two *C. pseudotuberculosis* isolates from goats, the biofilm-forming CAPJ4 and, the non-biofilm-forming CAP3Wstrains. These findings may contribute to a better understanding of the molecular mechanisms underlying the biofilm formation used by these bacteria.

## Materials and Methods

### Bacterial Strains and Culture Conditions

*C. pseudotuberculosis* CAPJ4 and CAP3W strains were isolated from granulomatosis lesions of CLA in goats (12). The genome of the biofilm-forming strain CAPJ4 and the non-biofilm-forming CAP3W strain was sequenced and deposited in GenBank with the accession numbers NZ_CP026499 and NZ_CP026500, respectively. For proteomic analysis, the strains were cultivated in brain heart infusion broth (HiMedia, Mumbai, India) at 37°C for 48 h without agitation. All experiments, including cultivation, were performed in triplicates for each strain.

### Biofilm Assay

The *C. pseudotuberculosis* isolates were inoculated into tubes containing 3 mL of tryptic soy broth (TSB; Merck, Darmstadt, Germany) and incubated at 37°C for 48 h without agitation. The bacterial suspensions were then diluted in TSB until they reached an optical density (OD) of 0.2 at 600 nm. Next, 200-μL samples of the bacterial cultures were transferred to each well of a sterile flat-bottom culture plate and incubated at 37°C for 24 h. Quantitative analysis of the biofilm production, was then performed using the gentian violet test (22). The ODs were measured at 595 nm using a microplate reader (BioChrom, Cambridge, UK). The experiment was repeated three times, and the means were compared using Student's *t*-test, with differences considered statistically significant when *p* < 0.05.

### Scanning Electron Microscopy

Morphological differences between the CAPJ4 and CAP3W strains were observed using scanning electron microscopy (SEM). The strains were grown in TSB and incubated at 37°C for 48 h without agitation. SEM preparation was conducted according to a previously established protocol (23) with modifications. The bacterial pellet was centrifuged at 4,000 × *g* for 10 min at 20°C and then washed with sterile saline solution for 1 min and spread on a glass slide. Subsequently, the slides were fixed in 1% glutaraldehyde (Sigma Aldrich, Saint Louis, USA) for 12 h and immersed in gradient concentrations (50, 70, 80, 95, and 100%) of ethanol (Sigma Aldrich, Saint Louis, USA), for 20 min each. At the end of the dehydration process, the samples were immersed in 100% acetone (Merck, Darmstadt, Germany) and subjected to metallization in gold. The fragments obtained were observed using an electronic microscope TM-1000 (Hitachi, Tokyo, Japan).

### Protein Sample Preparation for LC-MS/MS Analysis

Following growth of bacterial strains, extracts of the whole bacterial lysates were prepared according to a previously described protocol (21, 24) with specific modifications. Briefly, bacterial cultures were centrifuged at 5,000 × *g* for 10 min at 4°C. The pellet was resuspended in 1 mL of lysis buffer containing 7M Urea (Merck, Darmstadt, Germany), 2M thiourea (Merck, Darmstadt, Germany), 3% sodium deoxycholate (SDC) (Thermofisher Scientific), 12.5 mM Tris-HCl pH 7.5, 1.5% dithiothreitol (DTT) (Sigma Aldrich, Saint Louis, USA), and 10 μL of Protease Inhibitor Cocktail powder (Merck, Darmstadt, Germany). The bacteria were then sonicated for five cycles of 1 min each, with 1 min intervals between cycles, on ice and the sonicated suspensions were centrifuged at 14,000 × *g* for 40 min at 4°C. The supernatant was concentrated using a Vivaspin® 500 column (Gottingen, Germany) with a threshold of 10 kDa (centrifuged five times at 15,000 × *g* for 10 min at 20°C, each cycle), and the lysis buffer was replaced with 50 mM ammonium bicarbonate (NH_4_HCO_3_) (Sigma Aldrich, Saint Louis, USA) at pH 8.0. Protein samples were quantified using the Lowry method (Bio-Rad, California, USA). For tryptic digestion, the extract (2 μg/μL) was mixed with 50 mM NH_4_HCO_3_, denatured with 0.1% (w/v) RapiGEST SF surfactant (Waters, Milford, USA) at 80°C for 15 min, reduced using 10 mM DTT for 30 min at 60°C, and alkylated with 10 mM iodoacetamide (Sigma Aldrich, Saint Louis, USA) in a dark chamber at 23–25°C for 30 min. Subsequently, the proteins were enzymatically digested with 5 μg of sequencing-grade modified trypsin (sequencing grade modified trypsin; Promega, Madison, USA) at 37°C for 18 h. The digestion process was stopped by adding 10 μL of 5% (v/v) trifluoroacetic acid (TFA; Sigma Aldrich, Saint Louis, USA) and incubated at 37°C for 90 min. The resulting peptide extracts were centrifuged at 21,900 × g for 30 min at 6°C. Finally, the supernatants were collected, transferred to Waters Total Recovery vials (Waters, Milford, USA), supplemented with 5 μL of 1 N ammonium hydroxide (Sigma Aldrich, Saint Louis, USA), and stored at −70°C until use.

### Mass Spectrometry

LC-MS/MS analysis was performed using a nanoACQUITY ultra-performance liquid chromatography (UPLC) system connected to a Synapt G2-Si HDMS mass spectrometer (Waters, Milford, USA). A nanoACQUITY UPLC M-Class HSS T3 (1.8, 75μm × 150 mm–pH 3) column was used, along with a reversed-phase M-Class BEH C18 (5, 300μm × 50 mm–pH 10) column (Waters, Milford, USA). The analytical column temperature was maintained at 55°C. Bidimensional nanoUPLC tandem nano electrospray high-definition mass spectrometry (nano ESI-HDMS^E^), using multiplexed data-independent acquisition (DIA) experiments, was conducted using a reverse-phase gradient of 7–40% (v/v) acetonitrile (0.1% v/v formic acid) with a simulated 1D analysis and a delivery of 500 nL min^−1^ in a nanoACQUITY UPLC 2D Technology system over 60 min (25).

For all measurements, the mass spectrometer was operated with a typical m/z resolving power of at least 25,000 full width at half maximum (FWHM), an ion mobility cell filled with helium gas, and a cross-section resolving power of at least 40 Ω/Δ Ω (26). All analyses were performed using nano electrospray ionization in the positive ion mode with nanoESI (+) (Waters, Milford, USA) and a NanoLock-Spray (Waters, Milford, USA) ionization source. The lock mass channel was sampled every 30 s. The mass spectrometer was calibrated with an MS/MS spectrum from a solution of human [Glu^1^]-fibrinopeptide B (Glu-Fib) (100 fmol.μL^−1^).

Final instrument calibration was obtained by the double-charged precursor ion [M + 2H]^2+^ = 785.8426 signal. The exact mass signals from multiplexed ion mobility DIA scanning (HDMS^E^) were collected in an alternating low-energy and high-energy acquisition mode. Mass spectrometric analysis of tryptic peptides was performed using a mass spectrometer equipped with a T-Wave-IMS device in MS^E^ and HDMS^E^ modes, as described previously (27). The radio frequency offset (MS profile) was adjusted such that the nanoESI-HDMS^E^ data were effectively acquired from m/z 400 to 2,000 using the MassLynx v.4.1 software (Waters, Milford, USA), ensuring that any mass observed in the high-energy spectra of < m/z 400 arose from dissociations in the collision cell.

### Data Processing, Protein Identification, and Quantification

HDMS^E^ raw data were processed using Progenesis QI for Proteomics (QIP) v.2.0 software (Nonlinear Dynamics, Newcastle, UK) (28). Proteins were identified using the ProteinLynxGlobalServer v.2.4 software (PLGS) search against the *C. pseudotuberculosis* GenBank accession numbers NZ_CP026499 and NZ_CP026500. The reversed sequences were joined to the original sequences using the ProteinLynx Global Server (PLGS) v 3.0.2 (Waters, Milford, USA) database management tool. Reverse sequences were used to calculate the false positive rate during the identification process. The following parameters were used for peptide identification: digest reagent, trypsin; maximum missed cleavage, one; maximum protein mass, 600 kDa; modifications: carbamidomethyl of cysteine (fixed), acetyl N-terminal (variable), phosphoryl (variable), and oxidation of methionine (variable); search tolerance parameters: peptide tolerance, 10 ppm; fragment tolerance, 20 ppm; and maximum false discovery rate (FDR), 1%. Quantitative analyses were performed with relative quantitation using the Hi-N algorithm. Proteins identified with at least two peptides and present in at least two of the three replicates were considered significant (29). Proteins were considered as differentially abundant between the two strains if there was a significant (*p* < 0.05, ANOVA) change in the expression at ≥ 2-fold (log2 ratio ≥ 1.0).

### Bioinformatics Analysis

Proteins identified in the *C. pseudotuberculosis* CAPJ4 and CAP3W strains were analyzed using the following prediction tools: SurfG+ v2.0 (30) to predict subcellular localization. The clusters of orthologous groups (COG) database was used to predict Gene Ontology functional annotations (31). The protein-protein interaction network was predicted using the STRING database (Version 11.0) (32).

## Results

**Figure 1:**
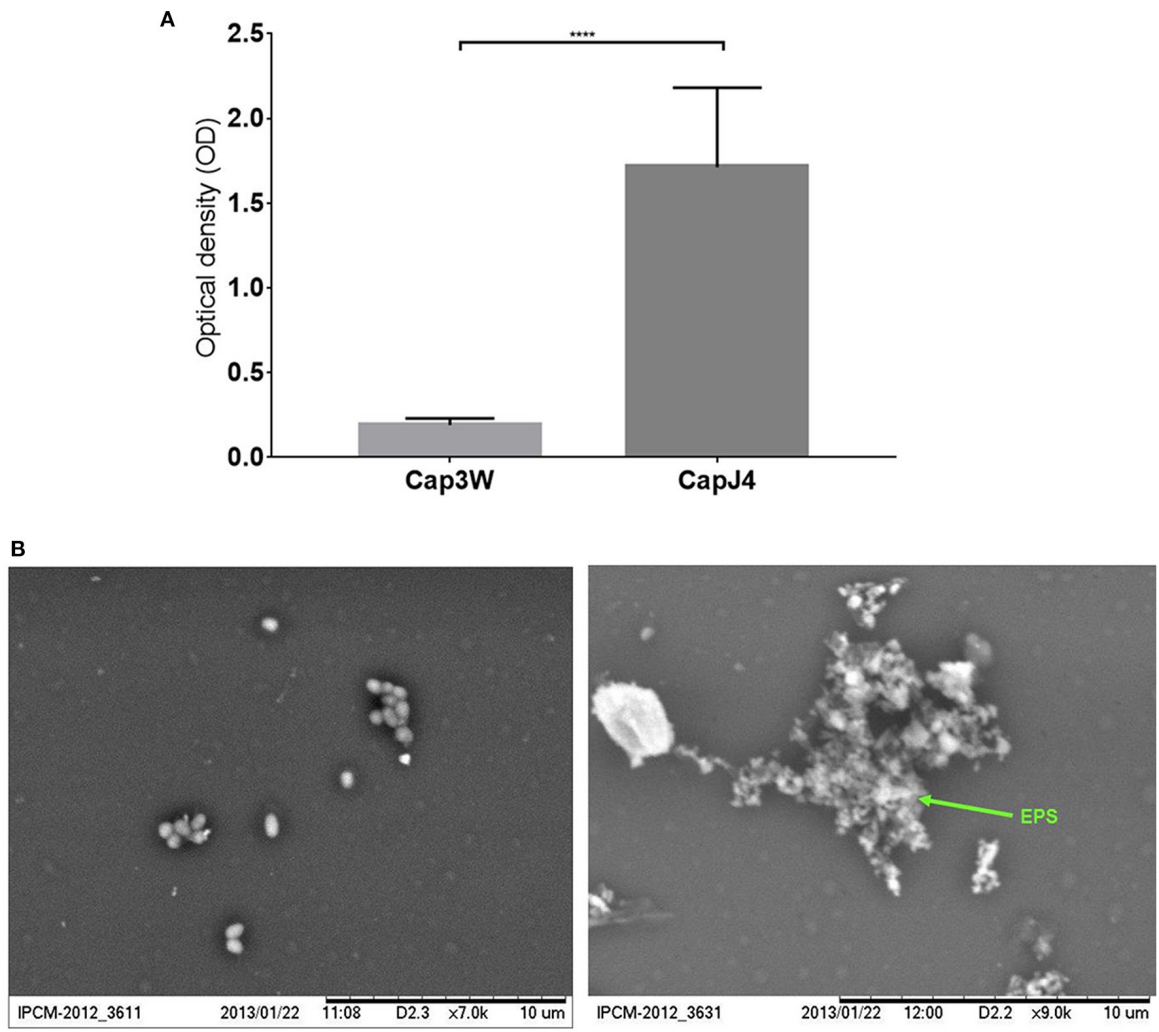
Confirmation of biofilm formation by *C. pseudotuberculosis* strains. **(A)** Optical density obtained from the Gentian violet staining assay performed with the CAPJ4 biofilm-forming and CAP3W non-biofilm-forming *C. pseudotuberculosis* strains. Results are represented as means of three independent assays. ****p < 0.0001, as determined by Student's *t-*test **(B)** Scanning electron microscopy analysis of the non-biofilm-forming CAP3W (left) and biofilm-forming CAPJ4 (right) *C. pseudotuberculosis* strains, respectively. Structures related to EPS of the biofilm-forming strain are shown in the picture on the right and indicated by the arrow.

Gentian violet staining assay which was performed to quantify biofilm formation revealed significant production of biofilms by the *C. pseudotuberculosis* CAPJ4 strain; however, *C. pseudotuberculosis* CAP3W did not present any biofilm formation (Figure 1A). These results were confirmed via SEM analysis (Figure 1B).

**Figure 2:**
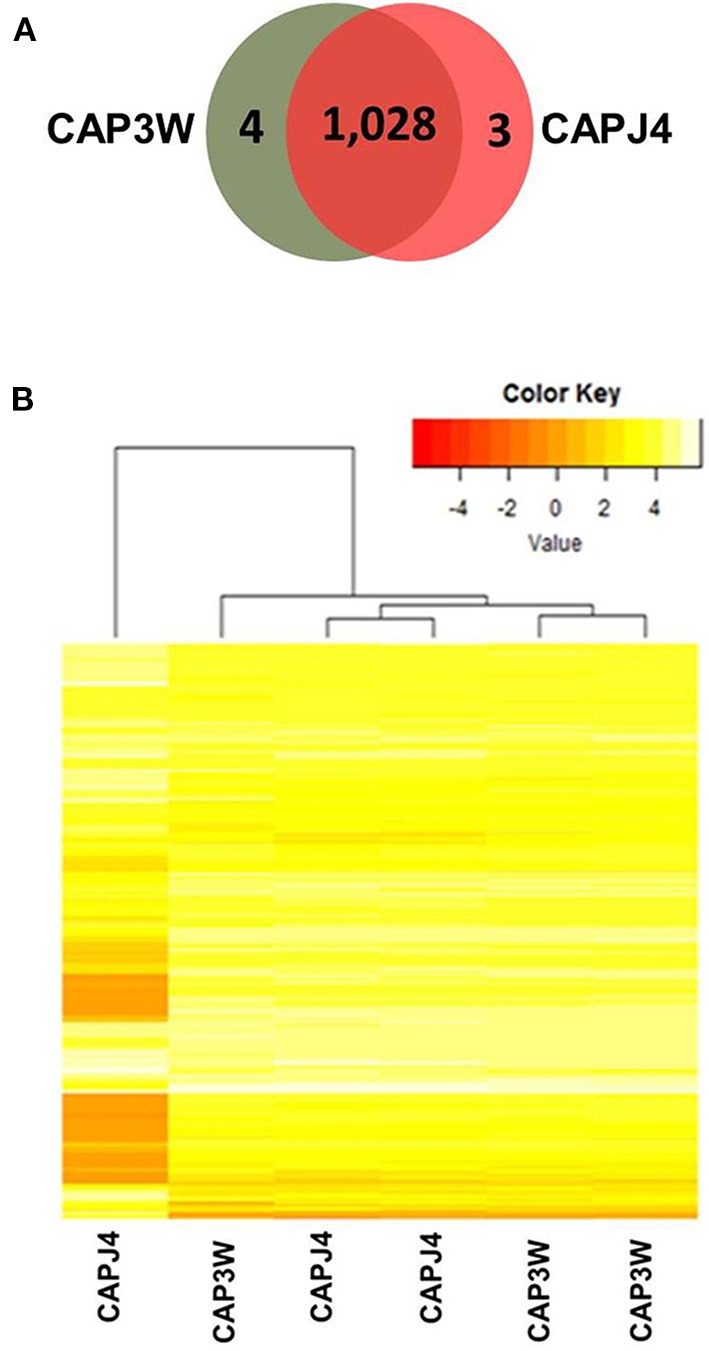
Label-free proteomic analysis of CAPJ4 and CAP3W strains. **(A)** A Venn diagram showing the number of unique and shared proteins between CAPJ4 and CAP3W *C. pseudotuberculosis* strains. **(B)** Heatmap analysis of the CAPJ4 and CAP3W strain proteomes (the columns represent biological replicates of each strain) by LC-MS^E^.

Differences in the functional genomes of CAP3W and CAPJ4 isolates were assessed at the protein level via proteomic analysis which detected 1,032 and 1,031 proteins in CAP3W and CAPJ4, respectively (Figure 2A).

Comparative proteomic analysis revealed a core-proteome composed of 1,028 proteins (Figure 2A, Supplementary File 1). In addition, we detected four and three proteins exclusively in the proteomes of CAP3W and CAPJ4, respectively (Figure 2A, Supplementary File 2). In CAP3W, the following proteins were detected: LPxTG domain-containing protein, methylated DNA-protein cysteine methyltransferase, and two hypothetical proteins. Meanwhile, in the CAPJ4 strain, ATP-dependent Clp protease ATP-binding subunit (ClpX), penicillin-binding protein FtsI (FtsI), and Dyp-type peroxidase family protein were detected.

Using label-free quantification, we evaluated the relative abundance of differentially expressed proteins in the proteomes of the CAPJ4 strain in comparison to that of the CAP3W strain (Figure 2B). In total, 40 differentially abundant proteins were identified; among them, 24 were more and 16 were less abundant in the CAPJ4 strain than in the CAP3W strain (Table 1).

**Table 1:** Statistically differentially abundant proteins between CAPJ4 and CAP3W strains.

**Accession**	**Accession**	**Description**	**Score**	**Log(2)_Ratio CAPJ4:CAP3W**	**Anova (*p*)**	**Subcellular localization^**(^#^)**^**
**CAP3W**	**CAPJ4**					
**Amino acid transport and metabolism**						
AUY56879.1	AUY58965.1	ABC transporter ATP-binding protein	1,327,192	1.39	0.028	CYT
AUY56653.1	AUY58741.1	Shikimate dehydrogenase	830,524	−1.33	0.034	SEC
AUY56909.1	AUY58994.1	D-3-phosphoglycerate dehydrogenase	2,645,591	−3.94	0.040	CYT
**Carbohydrate transport and metabolism**						
AUY56456.1	AUY58545.1	Galactose-1-phosphate uridylyltransferase	134,257	6.04	0.018	CYT
AUY55944.1	AUY58035.1	Glycerol-3-phosphate dehydrogenase	6,992,498	1.03	0.032	CYT
AUY56698.1	AUY58786.1	Glucose-6-phosphate 1-dehydrogenase	2,710,882	−1.23	0.023	CYT
**Coenzyme metabolism**						
AUY55493.1	AUY57582.1	Uroporphyrinogen decarboxylase	472,567	−1.24	0.030	SEC
**Energy production and conversion**						
AUY56941.1	AUY59025.1	Electron transfer flavo protein subunit alpha	1,147,859	1.33	0.015	CYT
AUY56959.1	AUY59043.1	ATP synthase subunit B	1,412,837	−1.72	0.025	MEM
**Lipid transport and metabolism**						
AUY56233.1	AUY58321.1	DegV family protein	105,856	−2.67	0.045	CYT
**Nucleotide metabolism**						
AUY56558.1	AUY58646.1	GTPase HflX	379,464	1.39	0.047	CYT
AUY56328.1	AUY58417.1	Inosine 5-monophosphate dehydrogenase	1,350,043	−1.22	0.010	CYT
AUY55377.1	AUY57467.1	Deoxyribose-phosphate aldolase	2,361,334	−1.62	0.042	CYT
**Intracellular trafficking secretion and vesicular transport**						
AUY55925.1	AUY58018.1	SECretion protein HlyD	5,029,537	1.85	0.002	SEC
AUY56627.1	AUY58715.1	Protein-export MEM protein	824,946	−2.25	0.016	PSE
**Cell wall/MEM and Envelope biogenesis**						
AUY55857.1	AUY57951.1	Trehalose corynomycolyl transferase B	2,992,162	2.79	0.022	SEC
AUY57300.1	AUY59381.1	N-Acetymuramyl-L-Alanine Amidase	2,000,788	1.39	0.015	CYT
AUY56015.1	AUY58105.1	D-alanyl-D-alanine carboxypeptidase	752,578	1.07	0.035	SEC
**RNA processing and modification**						
AUY55871.1	AUY57965.1	tRNA [guanine-N (7)]-methyltransferase	634,772	1.28	0.002	CYT
**Transcription**						
AUY55657.1	AUY57746.1	DtxR family transcriptional regulator	70,599	−1.07	0.046	CYT
**Translation, ribosomal structure and biogenesis**						
AUY56321.1	AUY58410.1	Function unknown	831,855	2.49	0.036	CYT
AUY55386.1	AUY57476.1	Surface antigen	4,196,807	1.16	0.039	CYT
AUY56857.1	AUY58943.1	Phenylalanyl-tRNA synthetase subunit beta	3,602,442	1.03	0.002	CYT
AUY56828.1	AUY58913.1	RNA pseudouridine synthase B	292,033	−1.45	0.037	CYT
**Function unknown**						
AUY56898.1	AUY58983.1	Function unknown	1,900,321	5.73	0.027	SEC
AUY57271.1	AUY59352.1	Function unknown	473,456	5.57	0.004	CYT
AUY56717.1	AUY58806.1	Function unknown	2,197,907	2.61	0.039	CYT
AUY55652.1	AUY57741.1	Function unknown	831,455	2.45	0.044	PSE
AUY55546.1	AUY57635.1	Function unknown	528,257	1.31	0.027	CYT
AUY56155.1	AUY58244.1	Function unknown	274,436	1.17	0.009	MEM
AUY56149.1	AUY58238.1	Function unknown	92,456	−1.03	0.032	CYT
AUY56008.1	AUY58098.1	Function unknown	332,779	−1.71	0.007	CYT
AUY55460.1	AUY57549.1	Function unknown	124,758	−8.89	0.010	MEM
**General function prediction only**						
AUY55918.1	AUY58012.1	Function unknown	126,931	4.33	0.002	SEC
AUY56282.1	AUY58369.1	GTPase Era	920,853	1.75	0.010	CYT
AUY56535.1	AUY58623.1	Surface antigen	2,760,137	1.16	0.024	PSE
AUY56107.1	AUY58196.1	Acetyltransferase	1,165,897	1.14	0.042	CYT
AUY55744.1	AUY57835.1	SEC hydrolase	2,073,888	1.14	0.026	SEC
AUY56713.1	AUY58801.1	ABC transporter ATP-binding protein	67,807	−1.00	0.003	CYT
AUY55874.1	AUY57968.1	Glycosyltransferase	214,642	−2.81	0.019	CYT

# *CYT, cytoplasmic; MEM, membrane; PSE, potentially surface-exposed; SEC, secreted*.

**Figure 3:**
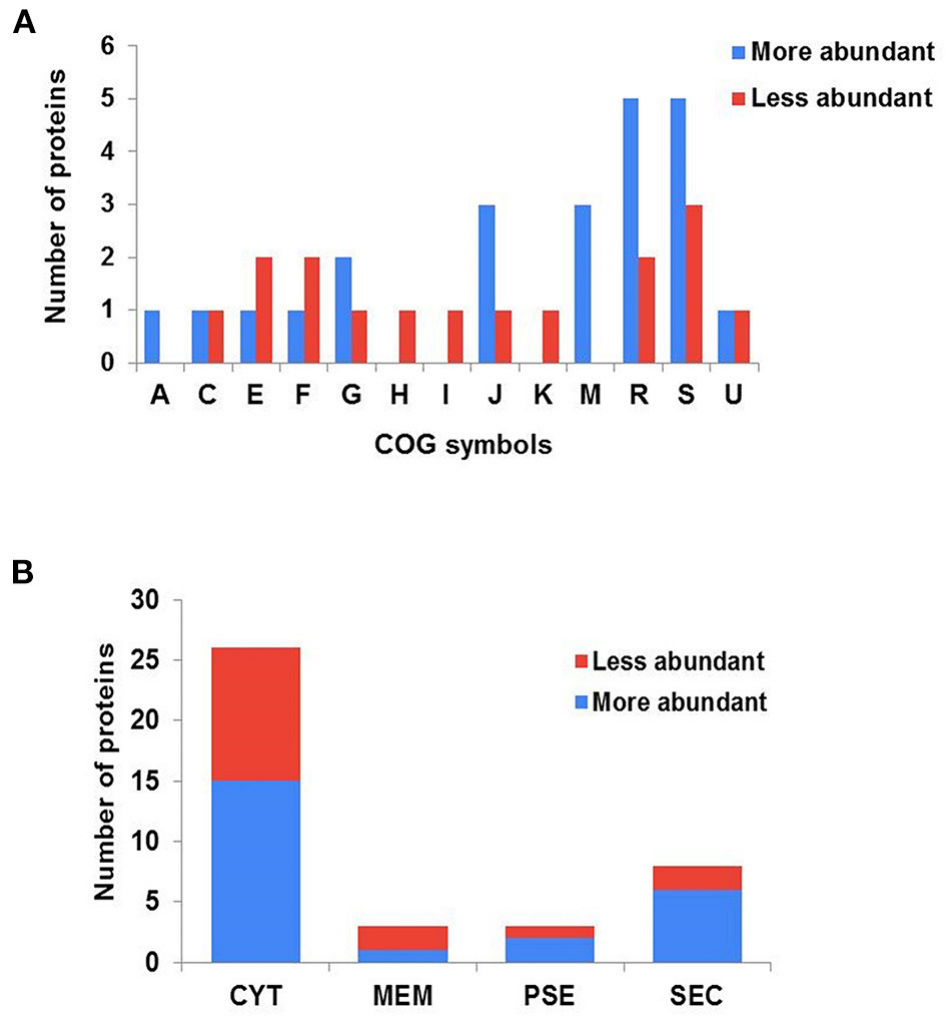
Clusters of orthologous groups (COG) analyses and prediction of the subcellular localization of differentially abundant proteins. **(A)** Categorization of differentially abundant proteins according to biological processes, as assessed by COG. (A) RNA processing and modification; (C) energy production and conversion; (E) amino acid transport and metabolism; (F) nucleotide metabolism; (G) carbohydrate transport and metabolism; (H) coenzyme metabolism; (I) lipid transport and metabolism; (J) translation, ribosomal structure and biogenesis; (K) transcription; (M) cell wall/membrane and envelope biogenesis; (R) general function prediction only; (S) function unknown; (U) intracellular trafficking secretion and vesicular transport. **(B)** Prediction of the subcellular localization of differentially abundant proteins. CYT, cytoplasmic; MEM, membrane; PSE, potentially surface-exposed; and SEC, secreted.

The forty differentially abundant proteins were classified into functional groups using Clusters of Orthologous Groups (COG) analysis. According to this analysis, the proteins were classified into 13 differential functional categories between CAP3W and CAPJ4 (Figure 3A). Twenty-seven proteins amongst the differentially abundant proteins, had known or predicted functions the majority of which are related to cellular metabolism. Interestingly, among these proteins, 50% (11 proteins) and 31% (five proteins) were classified as poorly characterized or of unknown function, respectively. These results encourage further studies to evaluate the role of these proteins in *C. pseudotuberculosis* pathophysiology (Table 1).

The subcellular localization of the differentially abundant proteins was predicted to be cytoplasmic, 36 proteins; membrane, 3; potentially surface-exposed, 3; and secreted, eight proteins (Figure 3B). Furthermore, the clustering analyses showed interesting protein-protein associations among more abundant and exclusive proteins of the CAPJ4 strain. N-acetylmuramoyl-L-alanine amidase (CwlM), penicillin-binding protein (FtsI), and ClpX presented a high edge confidence in the associations between proteins (Supplementary Figure 1).

## Discussion

The ability to form biofilms represents a suitable strategy developed by microorganisms with the objective of surviving in high stress conditions, including exposure to the host's immune response and antibacterial drugs (33). However, studies related to the mechanisms and molecules involved in the formation of biofilms by *C. pseudotuberculosis* are scarce (12). In this study, we evaluated the differences between the proteome of two *C. pseudotuberculosis* isolates from goats, the biofilm-forming CAPJ4, and the non-biofilm-forming CAP3Wstrains.

Through a biofilm formation assay, we confirmed that CAPJ4 is a biofilm-forming strain, while CAP3W is not (Figure 1). This difference may provide an advantage to the CAPJ4 strain to colonize various host tissues and survive in the environment, when compared to the CAP3W strain. Various studies have shown that biofilm-forming strains are more persistent during the pathogenesis process and consequently induce severe clinical symptoms of the associated diseases. This can be directly related to the increased capacity of the pathogens to avoid the host immune response and to resist different stress conditions and antimicrobial drug action (34–37).

Biofilms are heterogeneous in nature, and consist of self-secreted extracellular polymeric substances (EPS) and bacterial cells. EPS are composed of polysaccharides, DNA, proteins, and other macromolecular elements (38). To elucidate the differences between the functional genomes of CAPJ4 and CAP3W, and consequently to identify possible factors that could be related to biofilm formation by CAPJ4, we collected whole bacterial lysates and employed label-free proteomics and bioinformatic analysis to compare the proteomes of both isolates.

The expression of proteins associated with cell wall components like Trehalose corynomycolyl transferase B, N-Acetymuramyl-L-Alanine Amidase (CwlM) and D-alanyl-D-alanine carboxypeptidase was more in CAPJ4. The formation of a bacterial cell wall is required for bacterial biofilm formation and a study showed that inhibition of cell wall components biosynthesis, affects the biofilm formation in Gram-positive bacteria (39). CwlM and D-alanyl-D-alanine carboxypeptidase are involved in the peptidoglycan metabolism. Studies on *Mycobacterium smegmatis* and *Mycobacterium tuberculosis* showed that CwlM regulates biofilm formation by autolysis (40). Furthermore, CwlM expression was also observed in different stages of mycobacterial biofilm formation. In *E*. *coli*, mutant strains that do not encode D-alanyl-D-alanine carboxypeptidase exhibited defects in the biofilm formation (41). Trehalose corynomycolyl transferase B is associated with biosynthesis of the mycolic acid layer (42). In *Mycobacterium*, mycolic acid is involved in the pellicle biofilm formation, a type of biofilm. These pellicles are formed from bacterial growth at the media-air interface and it was shown that mycobacterial cells present in this pellicle are encapsulated in EPS (43).

The proteins found to be exclusively present within the CAPJ4 strain, ClpX and FtsI, as well as an upregulated protein CwlM, were correlating to the analysis of protein-protein interactions from *C. pseudotuberculosis* CAPJ4 strain (Supplementary Figure 1). ClpX belongs to the Clp protease family, which plays key roles in bacterial adaptation to various environmental stresses. In *Streptococcus suis*, the depletion of *clpX* reduces bacterial colonization and consequently mortality in mice (44). Clpx has been proposed to inhibit the FtsZ dynamics, which is essential for bacterial cell division (45, 46). FtsI is a penicillin-binding protein (Pbp) belonging to a cluster of membrane molecules that recruits FtsZ and promotes cytokinesis (47).

Pbps are essential to the bacterial cytoplasmic membrane and are responsible for the synthesis and remodeling of peptidoglycans. Pbps may participate in the elongation, septation, and adhesion of prokaryotic cells (48). Moreover, inactivation of genes encoding Pbps in *Escherichia coli* causes a decreased ability to form biofilms (49).

Another group of proteins exclusive to the *C. pseudotuberculosis* CAPJ4 biofilm-forming strain is Dyp-type peroxidase family proteins (DyPs), which are widely distributed among bacteria and may present functions analogous to those played by peroxidases in the presence of a substrate (50). In *Streptomyces* sp., DvPs are involved in the GlxA-dependent bacterial morphogenesis cascade (51). Although DyPs have functions related to the oxidative degradation of peroxidase substrates (50), their roles in biofilm formation are not yet clear.

Additionally, electron transfer flavo protein subunit alpha (EtfA) was upregulated in *C. pseudotuberculosis* CAPJ4. EtfA has oxidoreductase and redox-related functions influenced by RsaE in *Staphylococcus aureus*. RsaE is a conserved small regulatory RNA (sRNA) that contributes to extracellular eDNA release and biofilm matrix switching toward polysaccharide intercellular adhesin production in *S. epidermidis* (52). *etfA* is also reportedly produced by *Marinobacter hydrocarbonoclasticus* during biofilm formation on dexadecane (53).

The formation of biofilms increases bacterial density, promoting a communication process called quorum sensing (QS). In *Salmonella enteritidis*, several proteins are overexpressed in the presence of inductors related to QS, including the phenylalanyl-tRNA synthetase subunit beta (PheT) (54). PheT was also found to be upregulated in *C. pseudotuberculosis* CAPJ4 in our study.

In this study, we also used COGs for phylogenetic classification of the proteins into functional groups and to predict their subcellular localization. The results from this analysis shows very relevant results in the characterization of proteins that may be related to bacterial mechanisms of biofilm formation. Among the differentially expressed proteins, we have detected proteins related to cellular metabolism (Figure 3). Previous studies in a different prokaryotic have shown that this group of proteins are involved in the biofilm formation (52, 55). A recent study showed that *Pseudomonas aeruginosa* redirects host metabolism to promote biofilm formation through active repression of *zwf* and the likely use of itaconate as the main carbon source, directing glucose to generate the EPS matrix (55).

Proteins related to carbohydrate metabolism, such as galactose-1-phosphate uridylyltransferase (GalT) and glycerol-3-phosphate dehydrogenase (GlpD) were induced in *C. pseudotuberculosis* strain CAPJ4. GalT is an enzyme involved in galactose metabolism via the Leloir pathway, and participates in exopolysaccharide biosynthesis, which is an important component of the biofilm matrix (38, 56). Genes of the Leloir pathway are reportedly required for biofilm formation in *Bacillus subtilis* (57). GlpD is an enzyme that belongs to the *glp* regulon and is involved in glycerol metabolism, which serves as a carbon source for several prokaryotic organisms (58). Interestingly, some studies have shown that glycerol metabolism is involved in biofilm formation by certain bacteria, such as *P. aeruginosa* (59), *Proteus vulgaris* (60), *Listeria monocytogenes* (61)*, and Yersinia pestis* (62).

In this study, a label-free proteomics approach was utilized to quantify and evaluate the differences between the proteome of the CAPJ4 and CAP3W strains. The different proteomic profiles generated enabled the validation of *in silico* data of the CAPJ4 and CAP3W genomes. Moreover, the quantitative changes detected in our proteomic analysis enumerate a set of proteins likely involved in the production of biofilm in prokaryotes. However, further studies are needed to assess the role of these proteins in the biofilm production in *C. pseudotuberculosis*. Altogether, these findings open a new perspective for studying biofilm formation in this pathogen, which could be used as targets to inhibit this process with the objective of minimizing bacterial persistence and hence, generating more effective treatments against CLA.

## Data Availability Statement

The datasets presented in this study can be found in online repositories. The names of the repository/repositories and accession number(s) can be found in the article/Supplementary Material.

## Author Contributions

MS, VA, RP, MC, and RM designed the project. VA, RP, and RM discussed the results of the experiments. MS, SM, and JR performed biofilm tests and protein extraction. HF and CPR performed mass spectrometry experiments. NS, WS, and TC analyzed proteomic data. WS, NS, TC, CCSR, and RM prepared the manuscript. All authors contributed to the article and approved the submitted version.

## Conflict of Interest

The authors declare that the research was conducted in the absence of any commercial or financial relationships that could be construed as a potential conflict of interest.

## Supplementary Material

Supplementary File 1 Total proteins identified in the core-proteome of CAPJ4 and CAP3W *C. pseudotuberculosis* strains.

Supplementary File 2 Proteins identified exclusively in the proteomes of CAP3W or CAPJ4.

Supplementary Figure 1 STRING protein-protein network analysis of additional induced and exclusive proteins detected in the CAPJ4 strain. The thickness of the line indicates the degree of confidence prediction of the interaction. All proteins had 100% identity and a median confidence score ≥ 0.400.

## References

[1] Dorella FA Pacheco LGC Oliveira SC Miyoshi A Azevedo V. *Corynebacterium pseudotuberculosis*: microbiology, biochemical properties, pathogenesis and molecular studies of virulence. Vet Res. (2005) 37:201–18. 10.1051/vetres:2005056 16472520

[2] Peel MM Palmer GG Stacpoole AM Kerr TG. Human lymphadenitis due to *Corynebacterium pseudotuberculosis*: report of ten cases from Australia and review. Clin Infect Dis. (1997) 24:185–91. 10.1093/clinids/24.2.185 9114145

[3] Trost E Ott L Schneider J Schröder J Jaenicke S Goesmann A. The complete genome sequence of *Corynebacterium pseudotuberculosis* FRC41 isolated from a 12-year-old girl with necrotizing lymphadenitis reveals insights into gene-regulatory networks contributing to virulence. BMC Genomics. (2010) 11:728. 10.1186/1471-2164-11-728 21192786 PMC3022926

[4] de Sá MD Rocha Filho JT Rosa DS de Sá Oliveira SA Freire DP Alcantara ME. Linfadenite caseosa em caprinos e ovinos: Revisão. Pubvet. (2018) 12:1–13. 10.31533/pubvet.v12n11a202.1-13

[5] Seyffert N Guimarães AS Pacheco LGC Portela RW Bastos BL Dorella FA. High seroprevalence of caseous lymphadenitis in Brazilian goat herds revealed by *Corynebacterium pseudotuberculosis* secreted proteins-based ELISA. Res Vet Sci. (2010) 88:50–5. 10.1016/j.rvsc.2009.07.002 19665155

[6] Simões M Simões LC Vieira MJ. A review of current and emergent biofilm control strategies. LWT Food Sci Technol. (2010) 43:573–83. 10.1016/j.lwt.2009.12.008

[7] Olson ME Ceri H Morck DW Buret AG Read RR. Biofilm bacteria: formation and comparative susceptibility to antibiotics. Can J Vet Res. (2002) 66:86–92. 11989739 PMC226988

[8] Lappin-Scott HM Bass C. Biofilm formation: attachment, growth, and detachment of microbes from surfaces. Am J Infect Control. (2001) 29:250–1. 10.1067/mic.2001.115674 11486266

[9] Costerton JW Stewart PS Greenberg EP. Bacterial biofilms: a common cause of persistent infections. Science. (1999) 284:1318–22. 10.1126/science.284.5418.1318 10334980

[10] Chen L Wen YM. The role of bacterial biofilm in persistent infections and control strategies. Int J Oral Sci. (2011) 3:66–73. 10.4248/IJOS11022 21485310 PMC3469879

[11] Syal K Bhardwaj N Chatterji D. Vitamin C targets (p)ppGpp synthesis leading to stalling of long-term survival and biofilm formation in *Mycobacterium smegmatis. FEMS Microbiol Lett*. (2017) 364:1–6. 10.1093/femsle/fnw282 27986825

[12] Sá M da CA Veschi JLA Santos GB Amanso ES Oliveira SAS Mota RA. Activity of disinfectants and biofilm production of *Corynebacterium pseudotuberculosis. Pesqui Vet Bras*. (2013) 33:1319–24. 10.1590/S0100-736X2013001100006

[13] Chandra R Puthukkichal DR Suman E Mangalore SK. Diphtheroids-important nosocomial pathogens. J Clin Diagnostic Res. (2016) 10:DC28–31. 10.7860/JCDR/2016/19098.9043 PMC5296432 28208859

[14] Alvarez L William A Castro I Valenzuela F Belchior SE. Survival capacity of *Corynebacterium pseudotuberculosis* biovar ovis in different soil types from Chubut, Argentine Patagonia. Rev Argent Microbiol. (2017) 49:105–9. 10.1016/j.ram.2016.09.004 28063624

[15] Broadbent JA Broszczak DA Tennakoon IUK Huygens F. Pan-proteomics, a concept for unifying quantitative proteome measurements when comparing closely-related bacterial strains. Exp Rev Proteomics. (2016) 13:355–65. 10.1586/14789450.2016.1155986 26889693

[16] Cordwell SJ Nouwens AS Walsh BJ. Comparative proteomics of bacterial pathogens. Proteomics. (2001) 1:461–72. 10.1002/1615-9861(200104)1:4<461::AID-PROT461>3.0.CO;2-S 11681200

[17] Rani A Babu S. Environmental proteomic studies: closer step to understand bacterial biofilms. World J Microbiol Biotechnol. (2018) 34:120. 10.1007/s11274-018-2504-x 30022302

[18] Pacheco LG Slade SE Seyffert N Santos AR Castro TL Silva WM. A combined approach for comparative exoproteome analysis of *Corynebacterium pseudotuberculosis. BMC Microbiol*. (2011) 11:12. 10.1186/1471-2180-11-12 PMC3025830 21241507

[19] Silva WM Seyffert N Ciprandi A Santos AV Castro TLP Pacheco LGC. Differential exoproteome analysis of two *Corynebacterium pseudotuberculosis* biovar ovis strains isolated from goat (1002) and Sheep (C231). Curr Microbiol. (2013) 67:460–5. 10.1007/s00284-013-0388-4 23699973

[20] Rees MA Stinear TP Goode RJA Coppel RL Smith AI Kleifeld O. Changes in protein abundance are observed in bacterial isolates from a natural host. Front Cell Infect Microbiol. (2015) 5:1–15. 10.3389/fcimb.2015.00071 26528441 PMC4604328

[21] Silva WM Folador EL Soares SC Souza GHMF Santos AV Sousa CS. Label-free quantitative proteomics of *Corynebacterium pseudotuberculosis* isolates reveals differences between Biovars ovis and equi strains. BMC Genomics. (2017) 18:1–14. 10.1186/s12864-017-3835-y 28595597 PMC5463331

[22] Merino N Toledo-Arana A Vergara-Irigaray M Valle J Solano C Calvo E. Protein A-mediated multicellular behavior in *Staphylococcus aureus. J Bacteriol*. (2009) 191:832–43. 10.1128/JB.01222-08 PMC2632097 19047354

[23] Freitas VD Sand ST Simonetti AB. Formação *in vitro* de biofilme por *Pseudomonas aeruginosa* e *Staphylococcus aureus* na superfície de canetas odontológicas de alta rotação. Rev Odontol UNESP. (2010) 39:193–200.

[24] Tavares GC Carvalho AF Pereira FL Rezende CP Azevedo VA Leal CA. Transcriptome and proteome of fish-pathogenic *Streptococcus agalactiae* are modulated by temperature. Front Microbiol. (2018) 9:2639. 10.3389/fmicb.2018.02639 30450092 PMC6224512

[25] Gilar M Olivova P Daly AE Gebler JC. Two-dimensional separation of peptides using RP-RP-HPLC system with different pH in first and second separation dimensions. J Sep Sci. (2005) 28:1694–703. 10.1002/jssc.200500116 16224963

[26] Lalli PM Corilo YE Fasciotti M Riccio MF De Sa GF Daroda RJ. Baseline resolution of isomers by traveling wave ion mobility mass spectrometry: investigating the effects of polarizable drift gases and ionic charge distribution. J Mass Spectrom. (2013) 48:989–97. 10.1002/jms.3245 24078238

[27] Distler U Kuharev J Navarro P Levin Y Schild H Tenzer S. Drift time-specific collision energies enable deep-coverage data-independent acquisition proteomics. Nat Methods. (2014) 11:167–70. 10.1038/nmeth.2767 24336358

[28] Kuharev J Navarro P Distler U Jahn O Tenzer S. In-depth evaluation of software tools for data-independent acquisition-based label-free quantification. Proteomics. (2015) 15:314–3151. 10.1002/pmic.201400396 25545627

[29] Silva JC Gorenstein MV Li GZ Vissers JPC Geromanos SJ. Absolute quantification of proteins by LCMSE: a virtue of parallel MS acquisition. Mol Cell Proteomics. (2006) 5:144–56. 10.1074/mcp.M500230-MCP200 16219938

[30] Barinov A Loux V Hammani A Nicolas P Langella P Ehrlichh D. Prediction of surface exposed proteins in *Streptococcus pyogenes*, with a potential application to other Gram-positive bacteria. Proteomics. (2009) 9:61–73. 10.1002/pmic.200800195 19053137

[31] Tatusov RL Natale DA Garkavtsev IV Tatusova TA Shankavaram UT Rao BS. The COG database: new developments in phylogenetic classification of proteins from complete genomes. Nucleic Acids Res. (2001) 29:22–8. 10.1093/nar/29.1.22 11125040 PMC29819

[32] von Mering C Huynen M Jaeggi D Schmidt S Bork P Snel B. STRING: A database of predicted functional associations between proteins. Nucleic Acids Res. (2003) 31:258–61. 10.1093/nar/gkg034 12519996 PMC165481

[33] De la Fuente-Núñez C Reffuveille F Fernández L Hancock REW. Bacterial biofilm development as a multicellular adaptation: antibiotic resistance and new therapeutic strategies. Curr Opin Microbiol. (2013) 16:580–9. 10.1016/j.mib.2013.06.013 23880136

[34] Donlan RM Costerton JW. Biofilms: mechanisms of clinically relevant microorganisms. Clin Microbiol Rev. (2002) 15:167–93. 10.1128/CMR.15.2.167-193.2002 11932229 PMC118068

[35] Gomes F Saavedra MJ Henriques M. Bovine mastitis disease/pathogenicity: evidence of the potential role of microbial biofilms. Pathog Dis. (2016) 74:1–7. 10.1093/femspd/ftw006 26772653

[36] Di Domenico EG Cavallo I Bordignon V Prignano G Sperduti I Gurtner A. Inflammatory cytokines and biofilm production sustain *Staphylococcus aureus* outgrowth and persistence: a pivotal interplay in the pathogenesis of Atopic Dermatitis. Sci Rep. (2018) 8:1–13. 10.1038/s41598-018-27421-1 29955077 PMC6023932

[37] Sempere J de Miguel S González-Camacho F Yuste J Domenech M. Clinical relevance and molecular pathogenesis of the emerging serotypes 22F and 33F of *Streptococcus pneumoniae* in Spain. Front Microbiol. (2020) 11:1–16. 10.3389/fmicb.2020.00309 32174903 PMC7056674

[38] Gilan I Sivan A. Effect of proteases on biofilm formation of the plastic-degrading actinomycete *Rhodococcus ruber* C208. FEMS Microbiol Lett. (2013) 342:18–23. 10.1111/1574-6968.12114 23448092

[39] Bucher T Oppenheimer-Shaanan Y Savidor A Bloom-Ackermann Z Kolodkin-Gal I. Disturbance of the bacterial cell wall specifically interferes with biofilm formation. Environ Microbiol Rep. (2015) 7:990–1004. 10.1111/1758-2229.12346 26472159

[40] Wang C Zhang Q Tang X An Y Li S Xu H. Effects of CwlM on autolysis and biofilm formation in *Mycobacterium tuberculosis* and Mycobacterium smegmatis. Int J Med Microbiol. (2019) 309:73–83. 10.1016/j.ijmm.2018.12.002 30563740

[41] Miyamoto T Katane M Saitoh Y Sekine M Homma H. Involvement of penicillin-binding proteins in the metabolism of a bacterial peptidoglycan containing a non-canonical D-amino acid. Amino Acids. (2020) 52:487–97. 10.1007/s00726-020-02830-7 32108264

[42] Brand S Niehaus K Pühler A Kalinowski J. Identification and functional analysis of six mycolyltransferase genes of *Corynebacterium glutamicum* ATCC 13032: the genes cop1, cmt1, and cmt2 can replace each other in the synthesis of trehalose dicorynomycolate, a component of the mycolic acid layer of the cell envelope. Arch Microbiol. (2003) 180:33–44. 10.1007/s00203-003-0556-1 12740729

[43] Chakraborty P Kumar A. The extracellular matrix of mycobacterial biofilms: could we shorten the treatment of mycobacterial infections? Microb Cell. (2019) 6:105–22. 10.15698/mic2019.02.667 30740456 PMC6364259

[44] Roy S Zhu Y Ma J Roy AC Zhang Y Zhong X. Role of ClpX and ClpP in *Streptococcus suis* serotype 2 stress tolerance and virulence. Microbiol Res. (2019) 223:99–109. 10.1016/j.micres.2019.04.003 31178057

[45] Camberg JL Hoskins JR Wickner S. ClpXP protease degrades the cytoskeletal protein, FtsZ, and modulates FtsZ polymer dynamics. Proc Natl Acad Sci USA. (2009) 106:10614–9. 10.1073/pnas.0904886106 19541655 PMC2705540

[46] Goehring NW Beckwith J. Diverse paths to midcell: assembly of the bacterial cell division machinery. Curr Biol. (2005) 15:514–26. 10.1016/j.cub.2005.06.038 16005287

[47] Hale CA de Boer PAJ. ZipA is required for recruitment of FtsK, FtsQ, FtsL, and FtsN to the septal ring in *Escherichia coli*. J Bacteriol. (2002) 184:2552–6. 10.1128/JB.184.9.2552-2556.2002 11948172 PMC135003

[48] Gallant CV Daniels C Leung JM Ghosh AS Young KD Kotra LP. Common β-lactamases inhibit bacterial biofilm formation. Mol Microbiol. (2005) 58:1012–24. 10.1111/j.1365-2958.2005.04892.x 16262787 PMC3097517

[49] Ghosh AS Chowdhury C Nelson DE. Physiological functions of D-alanine carboxypeptidases in *Escherichia coli*. Trends Microbiol. (2008) 16:309–17. 10.1016/j.tim.2008.04.006 18539032

[50] Sugano Y Sasaki K Shoda M. cDNA cloning and genetic analysis of a novel decolorizing enzyme, peroxidase gene dyp from *Geotrichum candidum* Dec 1. J Biosci Bioeng. (1999) 87:411–7. 10.1016/S1389-1723(99)80087-5 16232492

[51] Petrus MLC Vijgenboom E Chaplin AK Worrall JAR Van Wezel GP Claessen D. The DyP-type peroxidase DtpA is a Tat-substrate required for GlxA maturation and morphogenesis in *Streptomyces*. Open Biol. (2016) 6:150149. 10.1098/rsob.150149 26740586 PMC4736821

[52] Schoenfelder SMK Lange C Prakash SA Marincola G Lerch MF Wencker FDR. The small non-coding RNA rsae influences extracellular matrix composition in *Staphylococcus epidermidis* biofilm communities. PLoS Pathog. (2019) 15:e1007618. 10.1371/journal.ppat.1007618 30870530 PMC6435200

[53] Vaysse PJ Prat L Mangenot S Cruveiller S Goulas P Grimaud R. Proteomic analysis of *Marinobacter hydrocarbonoclasticus* SP17 biofilm formation at the alkane-water interface reveals novel proteins and cellular processes involved in hexadecane assimilation. Res Microbiol. (2009) 160:829–37. 10.1016/j.resmic.2009.09.010 19786096

[54] Almeida FA Pimentel-Filho NJ Carrijo LC Bento CBP Baracat-Pereira MC Pinto UM. Acyl homoserine lactone changes the abundance of proteins and the levels of organic acids associated with stationary phase in *Salmonella Enteritidis*. Microb Pathog. (2017) 102:148–59. 10.1016/j.micpath.2016.11.027 27916690

[55] Riquelme SA Liimatta K Lung TWF Fields B Ahn D Chen D. *Pseudomonas aeruginosa* utilizes host-derived itaconate to redirect its metabolism to promote biofilm formation. Cell Metab. (2020) 31:1091–106.e6. 10.1016/j.cmet.2020.04.017 32428444 PMC7272298

[56] Holden HM Rayment I Thoden JB. Structure and function of enzymes of the Leloir pathway for galactose metabolism. J Biol Chem. (2003) 278:43885–8. 10.1074/jbc.R300025200 12923184

[57] Chai Y Beauregard PB Vlamakis H Losick R Kolter R. Galactose metabolism plays a crucial role in biofilm formation by *Bacillus subtilis*. mBio. (2012) 3:1–10. 10.1128/mBio.00184-12 22893383 PMC3419520

[58] Lin ECC. Glycerol dissimilation and its regulation in bacteria. Annu Rev Microbiol. (1976) 30:535–78. 10.1146/annurev.mi.30.100176.002535 825019

[59] Scoffield J Silo-Suh L. Glycerol metabolism promotes biofilm formation by *Pseudomonas aeruginosa*. Can J Microbiol. (2016) 62:704–10. 10.1139/cjm-2016-0119 27392247

[60] Wu YL Liu KS Yin XT Fei RM. GlpC gene is responsible for biofilm formation and defense against phagocytes and imparts tolerance to pH and organic solvents in *Proteus vulgaris*. Genet Mol Res. (2015) 14:10619–29. 10.4238/2015.September.9.3 26400293

[61] Crespo Tapia N den Besten HMW Abee T. Glycerol metabolism induces *Listeria monocytogenes* biofilm formation at the air-liquid interface. Int J Food Microbiol. (2018) 273:20–7. 10.1016/j.ijfoodmicro.2018.03.009 29558680

[62] Willias SP Chauhan S Motin VL. Functional characterization of *Yersinia pestis* aerobic glycerol metabolism. Microb Pathog. (2014) 76:33–43. 10.1016/j.micpath.2014.08.010 25220241 PMC4250381

