# Supplementary material for: Comparative Proteomic Analyses Between Biofilm-Forming and Non-biofilm-Forming Strains of Corynebacterium pseudotuberculosis Isolated From Goats
Source: Front Vet Sci. 2021 Feb 16;8:614011. doi: 10.3389/fvets.2021.614011 (PMC7921313; doi:10.3389/fvets.2021.614011)
Supplement: Supplementary File 1 — Total proteins identified in the core-proteome of CAPJ4 and CAP3W C. pseudotuberculosis strains. [file Data_Sheet_1.PDF]

Supplementary file 1: Total proteins identified in the core-proteome of CAPJ4 and CAP3W C. pseudotuberculosis strains.

								Normalized abundance						
Accession CAP3W	Acession CAPJ4	Peptide count	Unique peptides	Max fold ch	Confidence score	Anova (p)	Description	CAP3W	CAP3W	CAP3W	CAPJ4	CAPJ4	CAPJ4	Ratio_CAPJ4:CAP3W
AUY55513.1	AUY57602.1	7	4	1,19612	43,4306	0,687670043	2-oxoglutarate decarboxylase/2-succinyl-6-hydroxy-2, 4-cyclohexadiene-1-carboxylate synthase	3319,807	3565,372	3999,988	4627,831	5949,571	2442,563	1,196119919
AUY55687.1	AUY57776.1	70	49	2,016718	632,2497	0,175092677	accBC Acyl coenzyme A carboxylase	74092,7	74644,46	72646	51858,28	49205,23	8710,462	0,495855099
AUY55864.1	AUY57958.1	32	23	1,522558	207,4733	0,751909807	accD Propionyl-CoA carboxylase subunit beta	1151,601	3837,623	4016,794	6670,317	6961,557	80,3069	1,522557626
AUY56303.1	AUY58392.1	109	84	1,033728	936,5843	0,779222511	aceE Pyruvate dehydrogenase E1 component	55698,2	56065,44	62740,93	35975,09	36056,97	108358,3	1,033728419
AUY56362.1	AUY58451.1	44	33	1,552301	462,2797	0,452830922	aceF Dihydrolipoamide acyltransferase	65636,45	53936,84	72703,58	71564,51	52100,94	174806,2	1,552301444
AUY55977.1	AUY58068.1	50	34	1,378777	507,7487	0,421790531	ackA Acetate kinase	75457,1	72825,9	44076,86	68103,22	65765,09	5646,529	0,725280424
AUY56721.1	AUY58810.1	78	61	1,52701	549,717	0,610809182	acnA Aconitate hydratase	17609,01	23820,43	22851,83	33028,12	64888,79	241,221	1,52700996
AUY56720.1	AUY58809.1	9	4	1,590586	67,6672	0,614159559	acnR HTH-type transcriptional repressor AcnR	4625,653	7701,303	5667,732	4880,751	4782,844	18958,5	1,590585965
AUY56302.1	AUY58391.1	3	2	1,864945	21,0482	0,341688321	acp acyl carrier protein	7358,419	6613,632	6144,254	5353,323	5431,985	1,231508	0,536208747
AUY56165.1	AUY58254.1	6	6	1,64581	33,5579	0,266168522	acpS 4'-phosphopantetheinyl transferase	5409,664	3273,997	5489,283	7689,42	11496,48	4140,064	1,64580962
AUY55445.1	AUY57534.1	25	19	1,444107	162,6264	0,380809333	acsA Acetyl-CoA synthetase	19930,14	14861,68	12660,39	17787,31	15071,9	0	0,692469397
AUY55922.1	AUY58016.1	5	5	2,642508	32,416	0,337030213	adhA Alcohol dehydrogenase GroES-like protein	19142,91	5921,407	3712,179	5659,694	5230,15	0	0,378428353
AUY55600.1	AUY57688.1	17	13	1,662434	152,5287	0,344779845	adk Adenylate kinase	38463,52	47329,98	65561,62	56210,78	34196,96	636,5305	0,601527524
AUY56413.1	AUY58502.1	56	42	1,423551	513,1064	0,392592609	ag84 Antigen 84	145135,5	207991,4	170567,5	185150,6	179860,3	2868,126	0,70246886
AUY55723.1	AUY57814.1	26	20	1,199058	180,7625	0,393658079	ahcY S-adenosyl-L-homocysteine hydrolase	18866,84	13352,77	18686,61	12713,86	29741,3	0	0,83398776
AUY56575.1	AUY58664.1	31	25	1,506083	385,366	0,368178303	ahpC Alkyl hydroperoxide reductase subunit C	230924,8	250356,1	212874,3	224864,4	229276,2	6760,504	0,663974063
AUY56576.1	AUY58665.1	21	16	2,457478	212,7518	0,21209252	ahpD Alkyl hydroperoxide reductase AhpD	121078,7	150684,3	162146,6	76872,16	95960,07	3734,849	0,406921305
AUY56650.1	AUY58738.1	66	44	2,938942	456,3401	0,628571981	alaS alanyl-tRNA synthetase	14450,02	10827,04	9013,488	7739,648	7652,224	85386,08	2,938942288
AUY57017.1	AUY59101.1	4	2	2,047851	24,7287	0,332453253	alsD Alpha-acetolactate decarboxylase	2817,376	4515,129	3620,735	2825,227	2523,424	0	0,488316781
AUY56455.1	AUY58544.1	12	6	4,16387	99,1092	0,475273971	amiB Metal- dependentamidase/aminoacylase/carboxypeptidase	720,9254	5565,217	1077,094	1836,901	1293,537	27529,12	4,163869675
AUY55279.1	AUY57369.1	34	22	2,377745	236,7363	0,191989512	amn AMP nucleosidase	22606,7	32570,03	23577,69	18579,41	13288,45	1253,614	0,420566579
AUY56629.1	AUY58717.1	4	4	1,203411	28,6058	0,40890595	apt Adenine phosphoribosyltransferase	3530,004	2312,395	1709,43	544,5588	622,1052	5108,691	0,83097146
AUY56218.1	AUY58306.1	42	33	2,129392	392,0145	0,129350912	arcB1 Ornithine cyclodeaminase	47861,48	37052,01	30690,64	26807,62	21435,58	6046,537	0,469617636
AUY56852.1	AUY58938.1	2	1	1,131156	11,6819	0,422264896	argF ornithine carbamoyltransferase	3475,426	2746,119	1832,696	3171,287	3949,073	0	0,884050998
AUY56850.1	AUY58936.1	21	20	3,215809	155,9117	0,250693185	argG Argininosuccinate synthase	41527,38	35431,04	36157,48	18608,79	16544,07	22,07923	0,310963774
AUY56849.1	AUY58935.1	29	18	2,939366	190,0092	0,341589563	argH Argininosuccinase lyase	6113,446	3374,01	4283,23	3890,044	6075,717	30511,32	2,939365637
AUY56855.1	AUY58941.1	2	1	4,312946	15,8945	0,251002972	argJ Glutamate N-acetyltransferase	14630,83	9284,835	7784,416	3591,305	3758,679	0	0,231860097
AUY56851.1	AUY58937.1	8	5	1,492285	69,0026	0,957130707	argR Arginine repressor	3493,461	4269,737	3175,918	2664,77	1641,217	12018,29	1,492284881
AUY56973.1	AUY59057.1	70	56	1,97852	530,9594	0,283086267	argS arginyl-tRNA synthetase	15808,45	11511,56	14141,21	22680,26	11443,41	47908,17	1,978519951
AUY56657.1	AUY58745.1	4	4	115,5745	25,0445	0,066100604	aroB 3-dehydroquinate synthase	23,02792	49,95129	62,05786	178,6362	665,0042	14763,2	115,5744993
AUY56655.1	AUY58743.1	27	22	7,028937	194,0856	0,063591272	aroC Chorismate synthase	4854,402	5612,033	2544,353	13777,8	9928,301	67745,91	7,028936992
AUY57003.1	AUY59087.1	7	6	2,050876	63,5495	0,808733386	aroE Shikimate 5-dehydrogenase	2431,501	2223,567	2011,723	1142,654	1504,794	11025,32	2,050876453
AUY56653.1	AUY58741.1	10	7	2,510345	83,0524	0,033805998	aroE1 Shikimate dehydrogenase	11666,85	11654,26	11288,38	6488,643	5043,68	2254,422	0,398351577
AUY56386.1	AUY58475.1	22	18	1,826	144,2677	0,349856211	aroG Phospho-2-dehydro-3-deoxyheptonate aldolase	10520,92	10044,39	7452,236	7196,89	8146,779	0	0,547644992
AUY57040.1	AUY59124.1	5	4	1,092269	55,9252	0,448643006	arsC Arsenate reductase	9694,281	11992,35	13040,05	17483,25	14269,98	39,92191	0,915525192
AUY55387.1	AUY57477.1	44	33	1,343979	333,3809	0,163564956	asd Aspartate-semialdehyde dehydrogenase	33398,04	26361,93	20611,05	21063,11	22246,05	16491,65	0,744059304
AUY56371.1	AUY58460.1	44	30	1,23219	275,5155	0,628384098	asnB Asparagine synthetase	5008,741	12627,35	6311,602	8527,3	8007,092	2900,674	0,811563269
AUY56757.1	AUY58845.1	16	12	1,958225	120,5806	0,053026338	aspA Aspartate ammonia-lyase	4156,863	4569,626	3534,832	5996,964	6247,519	11765,94	1,958224521
AUY55359.1	AUY57449.1	33	24	1,048144	300,0772	0,846565647	aspB aspartate transaminase	23591,54	17071,73	11710,74	11880,22	13167,98	24920,16	0,954067711
AUY55890.1	AUY57984.1	16	13	1,959397	124,92	0,304839022	aspC Aminotransferase AlaT	15014,26	16411,93	9764,173	9357,773	11501,18	163,0017	0,510361002
AUY56647.1	AUY58735.1	56	40	1,378599	435,3759	0,564549481	aspS Aspartyl-tRNA synthetase	41497,1	39808,02	31238,28	84864,55	70070,78	216,8603	1,378598782
AUY56957.1	AUY59041.1	43	32	1,884579	427,7949	0,32766162	atpA ATP synthase subunit alpha	125373,3	143901,6	119928,4	97457,71	108797,9	264,4298	0,530622582
AUY56954.1	AUY59038.1	4	3	5,390143	27,4499	0,325216096	atpC ATP synthase subunit epsilon	1243,129	2500,89	3596,384	3040,128	2304,233	34221,46	5,390143173
AUY56955.1	AUY59039.1	56	45	7,314235	556,0246	0,154458305	atpD ATP synthase subunit beta	13291,82	26726,07	38449,28	47209,22	47773,86	478944,3	7,314235491
AUY56960.1	AUY59044.1	3	2	1,899874	26,237	0,350318482	atpE ATP synthase subunit C	12137,16	23543,46	19460,85	14441,41	14582,34	0	0,52635068
AUY56959.1	AUY59043.1	20	13	3,286574	141,2837	0,025496645	atpF ATP synthase subunit B	23899,72	15215,22	43374,21	9948,913	8038,777	7111,133	0,304268176
AUY56956.1	AUY59040.1	40	31	1,102564	335,4738	0,586913646	atpG ATP synthase subunit gamma	27905,75	35711,86	40637,4	47989,62	39068,79	7498,432	0,906976548
AUY56958.1	AUY59042.1	35	26	3,214672	312,915	0,270657704	atpH ATP synthase subunit delta	11093,83	9567,156	15539,01	22473,76	10005,43	83891,92	3,214671966
AUY56879.1	AUY58965.1	22	12	2,622009	132,7192	0,028054354	atrC ABC transporter ATP-binding protein	9087,194	11515,02	12536,66	24218,77	18780,87	43890,78	2,622008932
AUY56043.1	AUY58133.1	5	3	1,687016	33,2113	0,360987504	bca Beta family carbonic anhydrase family protein	1651,212	944,037	873,5521	868,9536	1187,222	0	0,592762477
AUY56167.1	AUY58256.1	14	8	1,883563	103,5254	0,345338778	bcp Bacterioferritin comigratory protein	11446,21	15308,92	14340,77	9267,821	12550,36	0	0,530908835
AUY56027.1	AUY58117.1	2	1	2,076928	10,0038	0,329952442	betA Choline dehydrogenase	2431,448	2972,922	1395,506	1258,616	2015,391	0	0,481480394
AUY57156.1	AUY59241.1	30	26	2,713428	208,8058	0,37956367	Bifunctional phosphoribosylaminoimidazolecarboxamide formyltransferase/IMP cyclohydrolase	239,8694	211,8805	654,3101	108,1333	152,3368	2740,744	0,738165634
AUY56833.1	AUY58918.1	3	3	3,932547	17,3924	0,929712724	bioD Dethiobiotin synthetase	1789,636	1515,78	1193,058	653,922	489,9864	0	2,713427831
AUY56616.1	AUY58704.1	2	2	1,374649	10,5622	0,232131634	bioF 8-amino-7-oxononanoate synthase	1852,082	1987,857	2020,418	2332,638	1930,529	0	0,254288115
AUY55699.1	AUY57790.1	4	3	1,316602	26,7241	0,387072653	birA Biotin-(acetyl-CoA carboxylase) ligase	5961,125	6047,371	5573,176	7487,33	8280,67	7380,057	0,727458565
AUY56668.1	AUY58756.1	18	13	3,157939	110,0376	0,003399614	carA Carbamoyl-phosphate synthase small chain	125905	14480,97	18778,9	25149,34	25252,17	0	1,31660155
AUY56669.1	AUY58757.1	67	52	1,063399	521,2428	0,352299147	carB Carbamoyl-phosphate synthase large chain	14037,18	8016,326	6474,229	6457,929	5749,034	14619,98	0,316662207
AUY56048.1	AUY58139.1	17	14	1,94156	145,4696	0,8057862	carD Transcriptional regulator	48814,09	74192,01	69778,04	57633,16	40936,7	723,5542	0,940381064
AUY56100.1	AUY58191.1	63	43	1,179088	521,9685	0,303390087	cat1 Succinyl-CoA Coenzyme A transferase	5005,047	17622,36	18924,01	15019,72	11302,54	8918,045	0,515049664
AUY57292.1	AUY59373.1	9	7	2,876984	57,3638	0,946730762	cca tRNA nucleotidyltransferase	5045,448	6194,379	5303,696	2638,816	3111,485	0	0,84811298
AUY55307.1	AUY57397.1	3	2	1,362056	19,6245	0,287721133	cfp30B Antigen Cfp30B	4010,198	4531,699	3333,121	1774,977	1648,459	5295,016	0,347586281
AUY56916.1	AUY59001.1	2	2	1,145285	24,6444	0,3061224	cfp6 Low molecular weight protein antigen 6	2063,005	5763,181	3858,15	3269,887	3077,975	3854,256	0,734184331
AUY57253.1	AUY59335.1	11	6	2,373571	69,8482	0,864151944	chrA ChrA, CheY/winged-helix DNA-binding domain-containing	9743,694	7450,812	7722,213	5587,143	4896,259	14,16561	0,873144916
AUY57151.1	AUY59236.1	8	5	1,149627	54,3519	0,286187198	citE Citrate lyase subunit beta	21779,91	21165,56	21613,94	35970,81	37386,63	861,7668	0,42130619
AUY56821.1	AUY58906.1	14	11	1,89568	84,3414	0,59696942	ciuA Iron ABC transporter substrate-binding protein	307,1479	494,3683	1042,98	717,7893	2778,785	0	1,149626553
AUY56818.1	AUY58903.1	3	1	45,74847	20,6746	0,565603131	ciuD Iron ABC transporter ATP-binding protein	518,9616	499,3986	537,743	1171,892	600,7228	69416,73	1,895679827
AUY56542.1	AUY58630.1	2	1	1,507505	9,3074	0,25862698	clgR Transcriptional regulator ClgR	46835,14	42002,26	41718,01	52749,22	108165,3	35898,38	45,74847332
AUY55936.1	AUY58027.1	109	76	2,620494	830,7572	0,401814626	clpB ATP-dependent chaperone protein ClpB	15857,96	17825,5	14384,8	15201,22	18209,12	92552,28	1,507504592
AUY56039.1	AUY58129.1	111	73	1,356738	807,0456	0,348124702	clpC ATP-dependent Clp protease ATP-binding subunit	51362,27	68784,04	72706,49	82799,33	59345,18	0	2,620494257
AUY56210.1	AUY58298.1	13	12	1,483444	121,7563	0,38494805	clpP1 ATP-dependent Clp protease proteolytic subunit 1	23613,8	48886,66	39214,25	34698,86	38244,72	92778,88	0,737062235
AUY56209.1	AUY58297.1	15	9	3,738534	126,9325	0,429534948	clpP2 ATP-dependent Clp protease proteolytic subunit 2	4620,959	3321,566	2593,937	2333,963	2000,908	35056,05	1,483443708
AUY56827.1	AUY58912.1	8	7	6,897073	51,1798	0,644071577	cmk Cytidylate kinase	14421,95	10697,88	11377,19	62601,45	31697,12	157424	3,738533741
AUY55857.1	AUY57951.1	27	21	1,555203	299,2162	0,021779686	cmtB Trehalose corynomycolyl transferase B	100802,3	82108,01	137649,7	120811,1	74924,88	10385,02	6,897072627
AUY55859.1	AUY57953.1	78	58	1,539376	728,1296	0,338667932	cmtC Trehalose corynomycolyl transferase C	844,5031	1830,039	1495,214	1454,151	1254,581	0	0,643003033
AUY57076.1	AUY59161.1	4	3	2,809959	23,6764	0,379114016	coaA Pantothenate kinase	2152,835	2880,008	3095,802	1782,151	1110,648	0	0,649614046
AUY56675.1	AUY58763.1	2	1	1,160836	11,7981	0,282638243	coaBC Coenzyme A biosynthesis bifunctional protein CoaBC	5516,719	5447,647	5135,762	6112,936	6366,839	6209,825	0,355877138
AUY56871.1	AUY58957.1	14	8	2,753939	85,9398	0,003952265	coaE Dephospho-CoA kinase	411,2992	632,7033	661,4726	415,0295	365,7948	3915,95	1,160835528
AUY56510.1	AUY58598.1	6	5	1,04945	34,1884	0,626435957	cobB cobyrinic acid A,C-diamide synthase	42142,62	38873,03	39984,72	68424,97	46578,14	295,7317	2,753938963
AUY56780.1	AUY58868.1	33	24	1,081425	295,9472	0,465479069	cobG Precorrin-3B synthase	19591,97	13109,87	12136,77	14282,92	34205,6	1,084277	0,952880081
AUY56779.1	AUY58867.1	21	16	1,206221	165,7791	0,433808553	cobH Precorrin-8X methylmutase	21972,33	14314,31	11811,67	20241,41	12928,21	24847,55	1,081425403
AUY56778.1	AUY58866.1	31	23	1,265544	204,0573	0,522002843	cobJ Precorrin-3B C(17)-methyltransferase	3693,785	4600,924	4922,295	3950,191	6493,545	0	1,206220503
AUY56777.1	AUY58865.1	18	11	1,52737	112,9502	0,395518316	cobK precorrin-6A reductase	6529,336	4276,835	4549,131	4028,214	6022,978	2,23565	0,790174226
AUY56775.1	AUY58863.1	2	1	1,857423	11,7463	0,371503472	cobL Precorrin-6Y C5,15-methyltransferase	2039,528	4428,544	3491,008	2679,418	2395,707	286,6486	0,654720269
AUY56776.1	AUY58864.1	6	3	2,686684	43,5859	0,282012933	cobM Precorrin-4 C11-methyltransferase	2210,557	2713,455	2809,708	1555,19	1323,347	0	0,538380455
AUY56781.1	AUY58869.1	9	4	1,062306	55,0748	0,290116474	cobN Cobaltochelatase	22363,36	17337,3	18504,32	15791,9	19753,63	19245,63	0,37220599
AUY56509.1	AUY58597.1	32	24	1,351896	263,8958	0,598641937	cobO Cob (I) yrinic acid a,c-diamide adenosyltransferase	4352,482	4196,74	4084,429	2317,159	1515,642	5512,335	0,941348386

AUY55370.1	AUY57460.1	10	5	1,308511	74,9542	0,302602012	cobQ1 Cobyric acid synthase	9593,91	12636,27	11179,68	10321,91	15129,52	81,30727	0,73970194
AUY56501.1	AUY58589.1	4	2	1,265226	18,3861	0,40752499	cobQ2 Cobyric acid synthase	5336,839	5801,961	5299,135	6072,292	6919,801	0	0,764227595
AUY56367.1	AUY58456.1	11	7	1,218083	69,1971	0,397545228	cobT Nicotinate-nucleotide--dimethylbenzimidazole phosphoribosyltransferase	36221,53	31880,28	27442,68	31263,51	31090,74	54026,9	0,790372556
AUY55222.1	AUY57313.1	12	9	4,562707	94,4598	0,447386032	CpCAP3W_0006 Hypothetical protein	1623,476	12532,13	16689,95	61700,17	70809,38	8229,657	1,218083195
AUY55230.1	AUY57321.1	21	12	1,753182	154,6854	0,199059783	CpCAP3W_0014 Periplasmic binding protein/LacI transcriptional regulator	33071,1	26053,47	23380,2	23383,92	22843,13	832,9465	4,562707405
AUY55233.1	AUY57324.1	21	18	2,022483	182,2256	0,317005166	CpCAP3W_0017 Hypothetical protein	5222,472	7485,542	6543,297	4638,644	4880,011	0	0,570391285
AUY55252.1	AUY57343.1	4	3	1,505827	34,4405	0,335505107	CpCAP3W_0036 FHA domain-containing protein	8148,091	7830,781	9918,951	7558,949	2566,573	28872,12	0,494441852
AUY55253.1	AUY57344.1	8	5	4,079149	51,7716	0,956655603	CpCAP3W_0037 Hypothetical protein	1703,595	5612,057	8707,845	7615,125	3697,796	54049,31	1,505826974
AUY55274.1	AUY57364.1	5	5	1,281293	40,2081	0,360046475	CpCAP3W_0058 Hypothetical protein	2731,198	2753,755	2730,948	3138,384	3021,389	4367,2	4,079148923
AUY55275.1	AUY57365.1	8	7	35,8976	40,478	0,116462101	CpCAP3W_0059 Peptidase family M20/M25/M40	92,35565	87,81165	91,31476	460,9047	179,5915	9105,058	1,281292538
AUY55280.1	AUY57370.1	3	3	1,060897	15,3184	0,122853114	CpCAP3W_0064 Hypothetical protein	39062,39	45452,72	60128,63	62798,51	66973,62	6568,895	35,89760068
AUY55281.1	AUY57371.1	18	11	1,253388	182,4255	0,591640061	CpCAP3W_0065 Lysozyme M1	10725,14	66519,67	77522,09	85493,46	108489,6	0	0,942598889
AUY55288.1	AUY57378.1	16	13	9030,852	153,7112	0,488526313	CpCAP3W_0072 Transcription factor Rok	2,096733	4,141395	6,710666	4,466847	4,479668	116929,7	1,253388469
AUY55292.1	AUY57382.1	24	15	3,328573	125,0171	0,359530826	CpCAP3W_0076 Hypothetical protein	2169,051	2446,165	3174,834	4855,882	3672,029	17401,84	9030,851537
AUY55297.1	AUY57387.1	8	6	2,270927	47,489	0,119583708	CpCAP3W_0081 Hypothetical protein	9346,924	5223,31	6908,394	5784,207	3673,881	0	3,328573249
AUY55305.1	AUY57395.1	11	8	1,520505	64,5074	0,320650628	CpCAP3W_0089 Hypothetical protein	16871,58	11078,07	21115,87	18417,29	5295,296	8556,645	0,440348799
AUY55306.1	AUY57396.1	9	7	2,949185	55,0939	0,274383221	CpCAP3W_0090 Hypothetical protein	2373,131	1342,974	1321,119	931,9966	776,0087	0	0,657676305
AUY55309.1	AUY57399.1	2	2	1,125925	10,8088	0,277955131	CpCAP3W_0093 Hypothetical protein	252,8381	1711,007	1416,404	2082,199	1723,71	0	0,33907671
AUY55313.1	AUY57403.1	2	2	1,350851	13,0542	0,527548128	CpCAP3W_0097 Alpha/beta hydrolase family protein	168,8327	28,74546	170,2798	204,5595	67,75623	0	1,125925496
AUY55315.1	AUY57405.1	4	1	1,159778	26,5637	0,457600703	CpCAP3W_0099 Hypothetical protein	12730,31	8128,375	6330,52	4420,154	4322,278	22791	0,740273965
AUY55320.1	AUY57410.1	15	11	4,5864	98,0764	0,828044541	CpCAP3W_0104 Decaprenylphosphoryl-D-2-keto erythropentose reductase	13742,81	5114,148	12815,31	4763,477	2131,191	11,02497	1,159777611
AUY55323.1	AUY57413.1	4	2	3,647133	34,4496	0,195195819	CpCAP3W_0107 Hypothetical protein	2396,587	3463,103	3558,579	1605,731	975,5109	1,133443	0,218035929
AUY55324.1	AUY57414.1	5	5	1,624922	34,233	0,236632281	CpCAP3W_0108 Hypothetical protein	23035,33	19882,76	17656,75	18949,87	17542,9	785,8511	0,27418794
AUY55342.1	AUY57432.1	12	10	1,318988	100,8124	0,337553527	CpCAP3W_0127 Glyoxalase/Bleomycin resistance protein/Dihydroxybiphenyl dioxygenase	63503,41	50939,06	72087,57	148412,4	97422,63	195,8945	0,61541424
AUY55344.1	AUY57434.1	17	13	3,536359	157,1804	0,530869367	CpCAP3W_0129 Hypothetical protein	969,2263	1366,019	1169,7	1160,774	1403,349	9830,625	1,318988295
AUY55349.1	AUY57439.1	4	2	2,234866	29,2021	0,322124717	CpCAP3W_0134 Hypothetical protein	10854,08	7084,657	16323,83	23508,59	26650,33	26413,32	3,536359429
AUY55386.1	AUY57476.1	51	33	33,24907	276,0137	0,024488809	CpCAP3W_0173 Surface antigen	4252,009	3402,991	2764,382	2461,207	3582,293	340391,3	2,234865591
AUY55390.1	AUY57480.1	3	3	3,640931	19,7161	0,416321107	CpCAP3W_0177 Hypothetical protein	1449,437	360,3864	597,3076	733,858	419,919	7610,419	33,24907391
AUY55407.1	AUY57496.1	9	6	2,554304	51,3387	0,528851686	CpCAP3W_0194 Hypothetical protein	10809,78	7991,547	10214,78	9207,532	16743,97	48164,45	3,640930585
AUY55408.1	AUY57497.1	27	19	1,212606	221,9762	0,222873192	CpCAP3W_0195 Hypothetical protein	6987,43	4405,508	5072,365	9036,751	10929,18	0	2,554304141
AUY55418.1	AUY57507.1	13	7	1,744044	79,5186	0,461606553	CpCAP3W_0206 Metallo-beta-lactamase superfamily protein	53872,85	44794,94	37483,46	43864,29	34185,84	16,27313	1,212606415
AUY55426.1	AUY57515.1	15	11	1,482439	128,4882	0,350627699	CpCAP3W_0214 Hypothetical protein	207318,5	82169,04	114147,6	159966,6	112302,7	8,38474	0,573379953
AUY55438.1	AUY57527.1	7	6	1,519004	78,1276	0,385210293	CpCAP3W_0226 Cold shock protein CspA	8848,096	19229,18	22454,09	12026,77	19349,8	45381,86	0,67456386
AUY55446.1	AUY57535.1	18	15	1,338024	130,3095	0,522049627	CpCAP3W_0234 Secreted hydrolase	6516,506	6665,966	4352,261	6863,587	6241,36	0	1,519004451
AUY55451.1	AUY57540.1	3	1	2,641253	15,9157	0,392549582	CpCAP3W_0239 Serine proteases of the peptidase family S9A	6776,654	5278,049	4282,079	3312,148	2873,091	0	0,747370808
AUY55456.1	AUY57545.1	11	9	473,3824	78,554	0,300574179	CpCAP3W_0244 HTH-type transcriptional regulator	8599,559	1025,121	489,1973	0,905972	20,45916	0	0,378608196
AUY55460.1	AUY57549.1	2	2	1,221473	12,4758	0,009706153	CpCAP3W_0248 Hypothetical protein	17275,68	11243,6	14381,42	23179,89	8059,269	21162,91	0,002112457
AUY55466.1	AUY57555.1	9	7	1,520215	90,0181	0,763976703	CpCAP3W_0254 Pyruvate formate-lyase	12601,95	24669,21	22530,24	20111,4	19226,07	0	1,221473407
AUY55469.1	AUY57558.1	6	5	2,771939	59,2325	0,378009859	CpCAP3W_0257 Hypothetical protein	19990,69	4339,281	4494,62	5966,081	4432,631	0	0,657801854
AUY55478.1	AUY57567.1	28	21	1,597652	187,1699	0,333472685	CpCAP3W_0266 Hypothetical protein	2816,641	2281,159	2262,913	2080,512	2520,855	7158,491	0,360758355
AUY55480.1	AUY57569.1	4	3	2,742547	26,8022	0,463552644	CpCAP3W_0268 Putative DNA-binding (excisionase) protein	3635,114	75,54459	166,5159	178,4688	102,0656	10352,8	1,597651942
AUY55486.1	AUY57575.1	6	5	1,912789	43,2656	0,813539637	CpCAP3W_0274 Hypothetical protein	1235,115	2899,528	2876,73	1954,063	1711,461	0	2,7425469
AUY55503.1	AUY57592.1	5	3	1,4671	34,2538	0,34979634	CpCAP3W_0291 ABC transporter	1695,07	3952,083	2997,433	3083,64	2808,656	0	0,522796848
AUY55506.1	AUY57595.1	2	1	2,101056	11,3309	0,387463865	CpCAP3W_0294 Hypothetical protein	25355,75	46190,27	42546,29	33999,94	20302,43	0	0,681616885
AUY55515.1	AUY57604.1	3	1	2,478795	26,32	0,336740865	CpCAP3W_0303 Glycosyl transferase group 1	9471,436	14149,19	18315,81	48362,98	27459,57	28130,6	0,475951242
AUY55546.1	AUY57635.1	4	4	25,10956	52,8257	0,027105125	CpCAP3W_0335 Hypothetical protein	3120,406	2877,844	1924,85	246,633	68,90809	0	2,478795195
AUY55553.1	AUY57642.1	2	1	11,41507	20,2321	0,063108112	CpCAP3W_0342 Hypothetical protein	489,0816	65,77844	284,9906	12,99606	60,5778	0	0,039825463
AUY55554.1	AUY57643.1	3	1	1,463353	21,5976	0,094537073	CpCAP3W_0343 Hypothetical protein	45438,87	45782,16	40074,24	45513,89	44208,34	0	0,087603499
AUY55558.1	AUY57647.1	11	9	2,21634	118,5244	0,376929208	CpCAP3W_0347 Hypothetical protein	58359,88	70908,82	54622,65	79399,04	81131,21	247035,5	0,683362214
AUY55559.1	AUY57648.1	18	16	1,036992	180,056	0,164551005	CpCAP3W_0348 SSU ribosomal protein S10p (S20e)	45921,65	45089,32	41067,66	52066,8	35909,9	48987,81	2,216339812
AUY55568.1	AUY57657.1	13	11	2,382194	144,0488	0,846702023	CpCAP3W_0357 LSU ribosomal protein L29p (L35e)	15469,44	16668,76	16632,44	18860,68	18284,84	79035,6	1,036992202
AUY55588.1	AUY57676.1	11	9	1,061891	133,8355	0,271336536	CpCAP3W_0378 LSU ribosomal protein L30p (L7e)	130131,8	64999,21	76985,69	83057,01	63904,35	109295,3	2,382194011
AUY55602.1	AUY57691.1	13	9	1,64964	138,8603	0,889436904	CpCAP3W_0393 L,D-transpeptidase catalytic domain, region YkuD	865,5087	982,1366	920,3611	917,1913	760,754	0	0,941715808
AUY55623.1	AUY57712.1	2	1	1,343891	16,2369	0,357355165	CpCAP3W_0414 Hypothetical protein	6564,01	7527,157	6013,627	11771,71	15217,92	29,02758	0,606192716
AUY55624.1	AUY57713.1	15	11	1,447932	100,0453	0,546030598	CpCAP3W_0415 Abhydrolase domain-containing protein 5	281206	354282	439405,8	629243,8	558714,7	368415	1,343890952
AUY55631.1	AUY57720.1	23	20	1,805392	267,9106	0,15595972	CpCAP3W_0423 Heat shock protein 60 family co-chaperone GroES	1948,701	1178,905	1435,936	1420,557	1107,171	0	1,447932343
AUY55634.1	AUY57723.1	6	6	1,18682	32,0944	0,347443947	CpCAP3W_0426 Hypothetical protein	1652,249	976,1317	1024,036	747,1318	1202,431	2385,198	0,553896241
CpCAP3W_00427	CpCAPJ4_00425	2	2	1,599351	9,6432	0,828978998	CpCAP3W_0427 Hypothetical protein	4236,095	4743,693	4391,668	4849,142	6356,504	10180	1,186819665
AUY55639.1	AUY57728.1	5	3	2,058525	40,5684	0,125992963	CpCAP3W_0432 Hypothetical protein	30146,37	22802	23045,5	20481,83	16429,82	5,004618	1,599350592
AUY55643.1	AUY57732.1	11	8	1,025636	81,5741	0,327681577	CpCAP3W_0436 Hypothetical protein	1668,329	3700,275	7168,629	7404,559	4821,663	632,4101	0,485784641
AUY55644.1	AUY57733.1	3	2	5,463406	12,198	0,808823799	CpCAP3W_0437 Sucrase ferredoxin-like protein	2284,349	1582,368	3207,874	9500,742	4690,649	24459,97	1,025635562
AUY55652.1	AUY57741.1	9	8	1,508918	83,1455	0,043599772	CpCAP3W_0445 Hypothetical protein	15873,34	27414,69	13642,52	19076,54	18438,33	214,4997	5,46340572
AUY55653.1	AUY57742.1	16	11	2,390089	140,8615	0,388449053	CpCAP3W_0446 Manganese ABC transporter substrate-binding protein	7416,383	6357,685	5425,626	4369,974	3605,336	57,73626	0,662726378
AUY55671.1	AUY57760.1	9	5	7,303986	51,1201	0,254510306	CpCAP3W_0466 Endonuclease/exonuclease/phosphatase family protein	6274,66	8287,146	7298,567	7456,728	6815,598	145395,5	0,418394484
AUY55677.1	AUY57766.1	18	10	1,365522	122,4383	0,382070683	CpCAP3W_0472 Hypothetical protein	5170,986	7220,312	6065,313	6524,072	5634,115	1357,973	7,303986245
AUY55691.1	AUY57782.1	10	8	2,566301	67,4074	0,378683168	CpCAP3W_0488 Hypothetical protein	3479,963	17185,26	16819,83	19412,64	17585,4	59199,89	0,732320845
AUY55695.1	AUY57786.1	6	5	1,830015	56,7716	0,202379388	CpCAP3W_0492 Hypothetical protein	3369,622	2789,154	2638,811	2180,45	2594,612	32,32399	2,566300689
AUY55709.1	AUY57800.1	7	5	1,309396	41,2442	0,317902275	CpCAP3W_0506 Hypothetical protein	911,7673	2177,526	1896,389	1663,465	2144,156	0	0,5464436
AUY55716.1	AUY57806.1	3	3	1,33535	19,9311	0,407510305	CpCAP3W_0513 Hypothetical protein	42166,95	34824,19	56084,16	83451,67	48731,89	45518,56	0,763711008
AUY55722.1	AUY57813.1	6	4	1,223503	92,2223	0,317282716	CpCAP3W_0519 Hypothetical protein	86955,74	161401,8	139768,4	153382,4	162930,4	912,3628	1,335350025
AUY55729.1	AUY57820.1	27	23	1,11271	256,3359	0,438733068	CpCAP3W_0526 Ribosomal protein S30AE family protein	10786,48	8697,331	6594,783	15059,41	12134,99	1823,498	0,81732537
AUY55732.1	AUY57823.1	11	9	19,55624	90,1153	0,778542164	CpCAP3W_0529 Haloacid dehalogenase-like hydrolase	845,6445	1966,677	160,6294	1898,141	1482,074	54759,53	1,112709544
AUY55733.1	AUY57824.1	7	7	11,98715	56,1083	0,19870723	CpCAP3W_0530 Hypothetical protein	175,3198	49,60911	92,36576	188,7203	577,6207	3037,116	19,55624
AUY55734.1	AUY57825.1	6	4	2,197069	30,8693	0,086067674	CpCAP3W_0531 Stearoyl-CoA 9-desaturase electron transfer partner	33882,61	18166,4	26989,17	74399,65	46047,66	53205,03	11,98714629
AUY55744.1	AUY57835.1	22	18	2,822931	207,3888	0,02589848	CpCAP3W_0541 Secreted hydrolase	55338,41	24882,19	43565,53	82984,29	54818,51	211636,9	2,1970692
AUY55747.1	AUY57838.1	18	14	1,895172	179,9227	0,117178687	CpCAP3W_0544 Hypothetical protein	3302,934	5265,386	3114,411	2312,287	319,4243	19509,07	2,822930562
AUY55757.1	AUY57848.1	10	7	1,075271	66,0554	0,731417404	CpCAP3W_0554 Hydrolase	1578,883	536,5936	455,93	901,6108	1712,233	151,1146	1,895171584
AUY55759.1	AUY57850.1	2	2	4,182549	12,3932	0,848360564	CpCAP3W_0556 Hypothetical protein	2969,811	1133,71	6665,075	1222,749	1213,845	138,0549	1,075270736
AUY55760.1	AUY57851.1	3	2	1,377735	19,997	0,15281069	CpCAP3W_0557 Hypothetical protein	9452,268	8657,515	8995,761	9589,719	9754,249	330,0245	0,239088642
AUY55761.1	AUY57852.1	34	22	1,473033	200,042	0,401022745	CpCAP3W_0558 Hypothetical protein	551,2351	475,2487	419,1075	290,7407	317,1352	1521,528	0,725829081
AUY55764.1	AUY57855.1	3	2	1,339992	11,8664	0,88780033	CpCAP3W_0561 Hypothetical protein	76886,59	71104,05	66807,92	79187,49	81110,87	0	1,473033139
AUY55777.1	AUY57868.1	51	38	1,125553	448,0459	0,386393217	CpCAP3W_0575 Methylmalonyl-CoA carboxyltransferase 5S subunit	77699,33	49831,63	17242,85	52237,81	40476,97	35909,81	0,746272969
AUY55778.1	AUY57869.1	59	42	2,336272	484,139	0,930213795	CpCAP3W_0576 Methylmalonyl-CoA carboxyltransferase 12S subunit	5756,037	11633,73	11255,15	14294,53	15232,9	37394,89	0,888452003
AUY55779.1	AUY57870.1	10	5	1,882837	81,259	0,109017589	CpCAP3W_0577 Clusters with Methylmalonyl-CoA carboxyltransferase	103129	55477,02	64222,49	163861,4	173622,3	82066,19	2,336272048
AUY55780.1	AUY57871.1	6	5	1,193588	41,6606	0,11328468	CpCAP3W_0578 Methylmalonyl-CoA carboxyltransferase 1.3S subunit	6840,666	5610,453	17219,81	17131,95	7686,947	39,70205	1,882837436
AUY55781.1	AUY57872.1	23	20	1,303513	188,3638	0,451161497	CpCAP3W_0579 Hypothetical protein	267,0969	319,9325	1545,851	2119,03	661,2093	0	0,837809777
AUY55796.1	AUY57888.1	3	1	1,275054	19,5909	0,548803232	CpCAP3W_0594 Hypothetical protein	32720,25	22041,59	31067,7	9200,617	7799,476	50314,32	1,303513445
AUY55807.1	AUY57899.1	5	4	1,304226	57,1297	0,373938509	CpCAP3W_0605 LSR2-like protein	12289,64	10941,65	7446,194	7700,863	6798,378	25511,14	0,784280256
AUY55818.1	AUY57909.1	7	6	1,23369	54,6803	0,840732915	CpCAP3W_0616 Flavin reductase	3198,765	12337,11	11464,46	7421,656	7333,141	18555,26	1,304226034

AUY55827.1	AUY57919.1	16	13	149,4559	112,4553	0,643360559	CpCAP3W_0625	SEC-C domain-containing protein	61,01594	6,979356	19,87732	69,91699	57,80059	13005,36	1,233690353
AUY55828.1	AUY57920.1	6	3	2,002204	30,1758	0,196874694	CpCAP3W_0627	Hypothetical protein	6777,447	2068,254	1901,838	1464,666	1877,308	2025,88	149,4558962
AUY55836.1	AUY57930.1	18	11	2,111934	129,5637	0,283703894	CpCAP3W_0636	GntR family transcriptional regulator	1918,65	2224,657	1879,448	1030,365	1488,25	333,1585	0,499449635
AUY55839.1	AUY57933.1	6	3	2,115256	33,3099	0,112930312	CpCAP3W_0639	HAD-family hydrolase	9669,812	18899,16	10526,63	29149,59	11535,73	42011,87	0,47349968
AUY55840.1	AUY57934.1	44	33	2,35633	337,5843	0,204254252	CpCAP3W_0640	Peptidoglycan recognition protein	649,5243	1180,17	955,9094	658,3232	523,8558	0	2,115255782
AUY55856.1	AUY57950.1	6	5	6,924821	30,8325	0,304014634	CpCAP3W_0656	Hypothetical protein	2721,255	3012,406	2583,253	2712,324	2685,306	52195,51	0,424388804
AUY55860.1	AUY57954.1	7	6	4,304529	44,7883	0,385281574	CpCAP3W_0660	Hypothetical protein	728,7593	1688,535	1799,183	1413,669	1496,584	15239,7	6,92482059
AUY55861.1	AUY57955.1	9	8	1,906408	60,7362	0,34551445	CpCAP3W_0661	Cutinase	9267,571	6463,635	5116,215	4516,612	4388,293	2030,541	4,304529421
AUY55870.1	AUY57964.1	7	5	7,020265	55,6133	0,097193202	CpCAP3W_0670	Hypothetical protein	11744,86	7605,987	7735,096	425,9134	607,4051	2824,933	0,524546729
AUY55874.1	AUY57968.1	3	3	3,689775	21,4642	0,01876566	CpCAP3W_0674	Glycosyltransferase	3793,041	5588,655	4728,837	4057,609	3806,426	44200,65	0,142444761
AUY55881.1	AUY57975.1	19	15	1,826654	115,5673	0,476367632	CpCAP3W_0681	VanW family protein	19277,91	24475,44	23994,56	17659,02	19408,95	20,54905	3,689774827
AUY55882.1	AUY57976.1	30	24	1,566644	225,0242	0,33917071	CpCAP3W_0682	Aldehyde dehydrogenase	16708,21	14922,42	15580	17518	12544,3	72,58233	0,547448913
AUY55904.1	AUY57997.1	16	13	1,419615	102,7427	0,36063579	CpCAP3W_0704	Hypothetical protein	96,19669	379,2887	378,0426	278,7266	322,5125	0	0,638307159
AUY55906.1	AUY57999.1	4	2	3,464943	22,5199	0,43084622	CpCAP3W_0706	Oxidoreductase	2243,21	2344,95	2262,864	1955,621	2071,905	19710,88	0,704416378
AUY55913.1	AUY58007.1	10	8	20,07938	69,3747	0,451202014	CpCAP3W_0714	Hypothetical protein	972,9129	1738,881	2728,261	32471,85	19305,65	57455,43	3,464942709
AUY55918.1	AUY58012.1	21	17	102,0534	126,931	0,002332124	CpCAP3W_0720	Hypothetical protein	335,6305	117,6749	101,1097	78,77004	127,1102	56374,08	20,07938103
AUY55919.1	AUY58013.1	6	5	1,307297	39,5466	0,485357356	CpCAP3W_0721	Membrane protein	1317,225	894,8835	1332,264	1457,3	1253,138	0,783098	102,0534177
AUY55924.1	CpCAPJ4_00727	3	2	1,626241	15,4795	0,402483828	CpCAP3W_0726	Amidohydrolase	3142,742	1724,764	1043,735	955,9763	2678,934	0	0,764936978
AUY55926.1	AUY58019.1	3	1	1,277715	16,9761	0,362649853	CpCAP3W_0728	ATP-bindingABC transporter domain-containing protein	2896,629	2253,442	2044,372	4162,897	5021,688	7,860406	0,614915045
CpCAP3W_00730	CpCAPJ4_00731	5	4	1,052969	31,8727	0,528439675	CpCAP3W_0730	Hypothetical protein	1870,77	4619,87	4000,582	3450,206	6513,261	0	1,277714648
AUY55930.1	AUY58022.1	4	3	14158,29	28,8662	0,432482496	CpCAP3W_0733	Oxidoreductase	4,68942	2,24015	1,651324	1,072823	0,817188	121488,9	0,94969574
AUY55933.1	AUY58024.1	11	9	1,006118	92,5829	0,480672284	CpCAP3W_0736	Hypothetical protein	18836,05	22195,19	19723,44	13801,69	14220,06	32363,5	14158,29261
AUY55935.1	AUY58026.1	14	10	4,550474	121,8788	0,774249537	CpCAP3W_0738	ABC transporter ATP-binding protein	4675,363	2747,811	1869,084	1059,3	982,7413	0	0,993919325
AUY55946.1	AUY58037.1	9	6	1,025867	59,1509	0,233695298	CpCAP3W_0749	Hypothetical protein	10130,94	12770,51	10394,98	8744,513	8750,89	14961,47	0,219757316
AUY55949.1	AUY58040.1	11	6	4,335956	64,8858	0,795076144	CpCAP3W_0752	Glycoside hydrolase family 76 protein	40359,05	7878,325	14976,69	32835,53	32520,96	208736,9	0,974785145
AUY55957.1	AUY58048.1	50	31	4,417878	450,1275	0,175458039	CpCAP3W_0761	Hypothetical protein	92702,75	54932,28	43769,85	176881,5	130890,3	537831,5	4,335956061
AUY55967.1	AUY58058.1	5	4	2,064452	55,6636	0,050239241	CpCAP3W_0771	Hypothetical protein	6945,139	16105,49	35773,5	43050,66	23658,67	54730,27	4,417877616
AUY55968.1	AUY58059.1	6	6	1,627477	56,1042	0,176054211	CpCAP3W_0772	Hypothetical protein	21982,39	17677,6	23425,78	33798,61	22810,61	46061,45	2,064452334
AUY55969.1	AUY58060.1	7	7	1,375407	80,7549	0,108711284	CpCAP3W_0773	Hypothetical protein	1034,908	317,3125	813,6939	1473,488	1475,975	29,55125	1,627477498
AUY55971.1	AUY58062.1	2	2	2,412022	9,4249	0,743171008	CpCAP3W_0776	Purine phosphoribosyltransferase	2729,07	3828,943	2711,253	1808,545	1932,581	101,8177	1,37540731
AUY55974.1	AUY58065.1	6	3	2,152247	31,5905	0,209608053	CpCAP3W_0779	Acetyltransferase	46121,95	45282,28	54653,8	88644,29	225708,6	0	0,414589854
AUY55975.1	AUY58066.1	49	37	2,018643	379,062	0,498957203	CpCAP3W_0780	Ferredoxin/ferredoxin-NADP reductase	26571,9	29942,09	35167,53	23849,48	21232,49	335,4313	2,152246563
AUY55982.1	AUY58073.1	12	12	4,741019	79,2871	0,292480832	CpCAP3W_0787	ABC transporter ATP-binding protein	6225,003	4398,909	3617,466	1718,736	1285,122	0,00685	0,495382326
AUY55986.1	AUY58077.1	12	6	2,152802	75,912	0,227775217	CpCAP3W_0791	Acetyltransferase	12812,91	10285,18	13523,68	9600,037	7411,174	0	0,21092514
AUY55989.1	AUY58080.1	4	2	5,180017	42,5981	0,328784161	CpCAP3W_0794	Hypothetical protein	1346,028	4839,262	4238,618	3863,295	4496,82	45635,9	0,464510906
AUY56005.1	AUY58095.1	4	3	3,725349	38,0497	0,279895617	CpCAP3W_0810	Cell wall channel	36133,84	25306,96	59778,82	21253,79	10194,69	1090,651	5,180016723
AUY56006.1	AUY58096.1	2	2	1,221584	27,24	0,121958543	CpCAP3W_0811	Hypothetical protein	9300,437	10226,86	12230,12	22634,94	14632,85	1526,57	0,268431251
AUY56007.1	AUY58097.1	8	5	3,270264	48,343	0,757380019	CpCAP3W_0812	Polyphosphate kinase 2	1228,627	1975,464	1643,72	683,0107	348,1274	451,2534	1,221583792
AUY56008.1	AUY58098.1	4	2	3,775851	33,2779	0,007382175	CpCAP3W_0813	Hypothetical protein	2824,549	132,31	88,78974	48,99036	137,4546	11313,47	0,305785729
AUY56013.1	AUY58103.1	7	7	1,36201	57,2535	0,896246955	CpCAP3W_0818	Rhodanese-related sulfurtransferase	5423,972	7601,807	8897,791	6895,977	9195,548	4,953306	3,775850817
AUY56024.1	AUY58114.1	17	12	1,493363	109,1079	0,393909114	CpCAP3W_0829	Hypothetical protein	5143,063	5854,182	2981,321	2534,549	2781,739	4044,175	0,734208838
AUY56030.1	AUY58120.1	6	4	2,014854	44,1068	0,202823176	CpCAP3W_0835	Na+/H+-dicarboxylate symporter	1283,532	744,9884	964,2574	823,5556	1426,175	3780,278	0,669629679
AUY56034.1	AUY58124.1	4	3	1,516459	35,935	0,329649216	CpCAP3W_0839	Caax amino protease family protein	8248,176	6289,668	7241,71	5613,403	8746,298	2,410899	2,014853518
AUY56044.1	AUY58134.1	5	5	6,320827	45,3027	0,369851351	CpCAP3W_0849	Hypothetical protein	2231,761	1665,652	1431,644	2116,357	1471,096	30096,59	0,659430962
CpCAP3W_00851	AUY58136.1	5	3	1,73063	43,3977	0,375746307	CpCAP3W_0851	Hypothetical protein	10452,83	4564,64	3924,076	2233,527	1112,77	29434,51	6,320826993
AUY56052.1	AUY58143.1	33	26	1,427548	226,1179	0,778613793	CpCAP3W_0858	SpoU rRNA methylase family protein	1113,178	2962,009	1630,478	1968,935	2027,893	0	1,730629793
AUY56054.1	AUY58145.1	3	3	1,579742	16,2185	0,397090771	CpCAP3W_0860	LacI family transcriptional regulator	571,8782	1957,615	1540,343	2358,888	4070,404	0	0,70050169
AUY56056.1	AUY58147.1	2	2	2,571995	15,9957	0,541100786	CpCAP3W_0862	Hypothetical protein	59686,73	6717,78	5715,947	8415,386	15545,3	4079,98	1,579742467
AUY56058.1	AUY58149.1	8	6	1,282713	55,1852	0,598072301	CpCAP3W_0864	Hypothetical protein	52658,41	43327,26	39867,49	57128,01	43088,19	74044,43	0,388803175
AUY56064.1	AUY58155.1	39	29	1,441667	347,6551	0,260131482	CpCAP3W_0870	Corynomycolyl transferase	12565,48	15060,83	5508,316	12371,8	14443,58	20953,71	1,282713092
AUY56069.1	AUY58160.1	10	7	1,99362	69,2683	0,286401284	CpCAP3W_0876	HIT family protein	33833,09	26602,19	46608,38	36077,38	17570,51	45,21503	1,441667241
AUY56075.1	AUY58166.1	8	5	1,051153	76,3612	0,316455062	CpCAP3W_0882	Hypothetical protein	17817,88	10114,48	8374,233	16463,19	13728,75	7971,849	0,50159998
AUY56077.1	AUY58168.1	13	10	1,444679	102,1521	0,860177207	CpCAP3W_0884	Glutathione peroxidase	7088,476	4767,008	4105,736	4992,225	6056,058	0	1,051153071
AUY56084.1	AUY58175.1	4	1	8,540189	22,7536	0,382986129	CpCAP3W_0891	Hypothetical protein	919,275	322,3167	329,871	379,859	702,0878	12338,64	0,692195414
AUY56087.1	AUY58178.1	8	4	1,096642	63,2805	0,356996671	CpCAP3W_0894	Hypothetical protein	11341,88	12734,37	16109	13631,64	20945,67	2066,59	8,540188952
AUY56088.1	AUY58179.1	14	11	1,965831	91,6242	0,559192738	CpCAP3W_0895	Glycine cleavage T protein	23634,79	17322,29	17705,14	10325,92	14442,29	5072,707	0,911874412
AUY56090.1	AUY58181.1	21	12	1,966528	169,8272	0,080879905	CpCAP3W_0897	Hypothetical protein	6445,111	12541,37	11044,53	7502,945	7766,935	1,202621	0,508690702
AUY56097.1	AUY58188.1	6	5	1,447263	32,4139	0,341657734	CpCAP3W_0905	Hypothetical protein	752,4958	895,9531	1191,113	1073,348	888,6741	0	0,508510429
AUY56099.1	AUY58190.1	6	1	2,202063	32,2165	0,383097531	CpCAP3W_0907	TIM-barrel containing enzyme	4531,655	9083,116	11651,76	20645,32	18840,74	16152,41	0,690959522
AUY56107.1	AUY58196.1	13	11	133,4176	116,5897	0,042318077	CpCAP3W_0915	Acetyltransferase	27,88416	191,6334	403,7526	1627,954	2148,139	79379,11	2,202062525
AUY56111.1	AUY58200.1	17	15	2,891246	120,6649	0,057397046	CpCAP3W_0919	LuxR family transcriptional regulator	84212,49	28476,55	26866,19	20957,89	24542,79	2767,514	133,4175764
AUY56115.1	AUY58204.1	18	10	2,27122	152,8465	0,185097152	CpCAP3W_0923	ABC transporter ATP-binding protein	6713,711	7843,276	5859,702	3632,746	5501,762	37236,28	0,345871601
AUY56116.1	AUY58205.1	24	19	1,344595	162,3559	0,705048067	CpCAP3W_0924	Antimicrobial peptide ABC transporter	8822,166	2703,151	5509,983	12343,28	10528,96	33,3336	2,271219636
AUY56120.1	AUY58209.1	7	3	1,922875	51,9606	0,595656619	CpCAP3W_0928	DoxX family membrane protein	6810,36	15743,43	12904,31	6717,198	11722,95	0	1,344594787
AUY56130.1	AUY58220.1	2	1	1,672194	13,3818	0,34675959	CpCAP3W_0939	Pyridoxamine 5'-phosphate oxidase	4029,827	3731,893	5369,406	6084,387	4561,084	11312,31	0,520054505
AUY56140.1	AUY58229.1	4	4	3,176489	19,5335	0,19355561	CpCAP3W_0949	Hypothetical protein	1987,903	2696,507	4297,094	2087,967	2485,41	23956,27	1,672193597
AUY56145.1	AUY58234.1	4	2	2,039822	23,4	0,529488815	CpCAP3W_0954	Ribosomal-protein-alanine acetyltransferase	3728,677	4591,078	3046,121	1964,13	2377,101	1230,764	3,176489068
AUY56149.1	AUY58238.1	15	8	3,332839	92,456	0,032406664	CpCAP3W_0958	Hypothetical protein	500,0125	519,6089	419,9475	187,8693	244,0654	0	0,490238865
AUY56154.1	AUY58243.1	3	2	2,257387	19,9217	0,235300431	CpCAP3W_0963	Deoxyribonucleotide triphosphate pyrophosphatase	1024,91	1294,4	1313,849	2426,205	3569,937	2205,305	0,300044484
AUY56155.1	AUY58244.1	5	3	1,58737	27,4436	0,008927996	CpCAP3W_0964	Hypothetical protein	43186,06	7660,514	8358,583	8845,116	7291,171	21161,35	2,257387082
AUY56168.1	AUY58257.1	11	9	2,631502	96,5529	0,735961898	CpCAP3W_0977	POSSIBLE CONSERVED MEMBRANE PROTEIN	22933,72	3342,111	1858,98	1944,783	3460,787	68631,22	0,629972827
AUY56169.1	AUY58258.1	10	6	1,27595	92,6319	0,784398256	CpCAP3W_0978	Antibiotic biosynthesis monooxygenase	32570,94	15593,14	26840,23	22729,78	54110,45	18861,51	2,631501562
AUY56171.1	AUY58260.1	23	16	2,135987	158,341	0,674994473	CpCAP3W_0980	Oxidoreductase	31778,84	19293,7	21124,57	18335,05	15465,29	0	1,275949904
AUY56175.1	AUY58264.1	14	10	1,326934	109,6011	0,335201385	CpCAP3W_0984	short-chain dehydrogenase	4024,114	4088,464	4310,986	3032,711	5125,196	8327,34	0,468167593
AUY56177.1	AUY58266.1	7	4	22,71688	48,2128	0,530561446	CpCAP3W_0986	Peptidase, S8A (subtilisin) family protein	9,567214	173,9474	112,2476	118,4569	73,28802	6527,05	1,32693381
AUY56181.1	AUY58269.1	5	3	2,598944	29,5321	0,321601496	CpCAP3W_0990	ABC transporter ATP-binding protein	79595,73	7606,302	7544,562	9055,341	5418,341	21982,12	22,71687612
AUY56193.1	AUY58281.1	64	47	2,475493	466,8802	0,615296303	CpCAP3W_1002	ABC transporter ATP-binding protein	629,4512	1820,984	1614,39	1429,196	1873,356	6759,894	0,384771621
AUY56195.1	AUY58283.1	3	3	2,603455	25,9647	0,263794037	CpCAP3W_1004	Thioesterase	196,1141	135,7924	120,1764	51,44433	24,70323	1100,83	2,475492762
AUY56196.1	AUY58284.1	3	2	1,452457	20,7369	0,825581733	CpCAP3W_1005	Hypothetical protein	17373,43	10137,65	10020,84	11722,29	14116,99	1,018526	2,603454826
AUY56201.1	AUY58289.1	5	4	2,492042	31,5271	0,383484018	CpCAP3W_1011	Hypothetical protein	7633,928	6964,915	5433,01	8732,146	11024,14	30163,93	0,688488573
AUY56202.1	AUY58290.1	18	15	3,96684	137,9579	0,122405187	CpCAP3W_1012	DSBA oxidoreductase	15120,79	15918,63	12422,13	5625,475	5315,756	14,98347	2,492042222
AUY56205.1	AUY58293.1	8	5	1,442777	72,8216	0,210096897	CpCAP3W_1015	Hypothetical protein	5329,557	5173,735	6097,531	7538,978	3967,18	0	0,25208984
AUY56206.1	AUY58294.1	9	7	4,859327	71,3413	0,373454578	CpCAP3W_1016	Hypothetical protein	7773,73	4871,177	3331,488	1583,416	1704,363	0	0,693107698
AUY56213.1	AUY58301.1	17	11	1,188455	111,7603	0,231984407	CpCAP3W_1023	Transcriptional regulator TetR	53531,15	58513,72	48947,89	59076,28	66003,34	384,2634	0,20578981
AUY56223.1	AUY58311.1	15	13	10,36147	163,7755	0,435966154	CpCAP3W_1033	LSU ribosomal protein L27p	3186,078	4424,043	2520,734	3355,971	1878,154	99736,38	0,841428445
AUY56228.1	AUY58316.1	13	6	1,245936	85,5432	0,486095939	CpCAP3W_1038	Phosphoglycerate dehydrogenase	21542,64	19809,83	26237,05	20336,81	14687,18	19223,99	10,36146557
AUY56231.1	AUY58319.1	10	9	6,36966	98,4251	0,163167147	CpCAP3W_1041	Protein yqeL	1164,109	1515,495	332,383	316,588	86,86329	69,41352	0,802609435
AUY56233.1	AUY58321.1	2	2	2,658117	10,5856	0,045494376	CpCAP3W_1043	DegV family protein	13356,81	11847,18	12813,26	19259,76	17012,15	64782,4	0,156994263
AUY56237.1	AUY58325.1	10	8	4,735	84,5567	0,141380412	CpCAP3W_1047	Ankyrin domain-containing protein	2089,087	743,41	914,1774	701,7702	474,1612	16564,57	2,658116738

CpCAP3W_01056	CpCAPJ4_01054	9	5	1,201343	56,8248	0,718459968	CpCAP3W_1056 ABC transporter substrate-binding protein	761,1679	5573,439	4672,89	2743,182	2198,401	4221,076	4,735000394
AUY56250.1	AUY58337.1	3	3	2,409162	21,2887	0,906233177	CpCAP3W_1061 Pyridoxal phosphate enzyme, YggS family	12044,29	8742,509	13827,82	11882,63	13466,12	58043,46	0,83240167
AUY56264.1	AUY58351.1	6	3	1,113756	36,8359	0,30617737	CpCAP3W_1076 Hypothetical protein	1604,45	330,3609	252,8187	111,3128	138,2026	2186,97	2,409161788
AUY56280.1	AUY58367.1	9	4	2,832059	50,1061	0,701584902	CpCAP3W_1093 Hypothetical protein	5610,325	4098,071	3080,6	2874,875	1639,982	0,93829	1,113756279
AUY56289.1	AUY58376.1	5	4	1,047253	29,2416	0,279111894	CpCAP3W_1102 Hypothetical protein	15879,34	13279,3	14786	14380,81	27581,02	0	0,353100033
AUY56291.1	AUY58378.1	20	18	1,045164	125,8572	0,413900667	CpCAP3W_1104 Chromosome segregation ATPase	235,7266	1034,183	602,3503	802,4053	988,9502	0	0,954879172
CpCAP3W_01107	AUY58381.1	4	3	2,060644	22,2097	0,479887209	CpCAP3W_1107 Guanyl-specific ribonuclease Sa3	4564,31	4489,18	3947,879	4313,765	6126,497	16350,93	0,956788051
AUY56301.1	AUY58390.1	8	7	3,134135	53,572	0,236464752	CpCAP3W_1115 N-acetyl glucosamine related protein	9623,602	8598,977	6988,728	7303,207	8695,814	63016,6	2,06064395
AUY56304.1	AUY58393.1	9	6	1,582976	69,1858	0,407536985	CpCAP3W_1119 Hypothetical protein	15207,95	12855,64	11691,78	17214,8	34102,06	11614,94	3,134134525
AUY56310.1	AUY58399.1	26	20	1,169935	192,9478	0,323634672	CpCAP3W_1125 NIF3 (NGG1p interacting factor 3)	13698,49	15148,48	18167,66	15528,54	8137,649	16519,49	1,582975611
AUY56311.1	AUY58400.1	21	16	3,151598	147,6504	0,459523269	CpCAP3W_1126 Zn-ribbon protein	4815,071	2442,336	1676,003	1319,555	1599,301	25235,66	0,854748351
AUY56312.1	AUY58401.1	7	6	1,659822	50,8665	0,757485876	CpCAP3W_1127 Bifunctional RNase H/acid phosphatase	39601,11	36529,8	106629,6	77820,1	32288,45	0	3,151597923
AUY56315.1	AUY58404.1	5	4	1,543063	34,7276	0,369061622	CpCAP3W_1131 Hypothetical protein	3390,603	2893,068	3469,251	3006,811	3269,063	44,62199	0,602474387
AUY56316.1	AUY58405.1	9	6	5,624191	54,4669	0,364412634	CpCAP3W_1132 Chad domain-containing protein	6635,68	1284,544	4310,05	12177,21	19717,09	36891,1	0,648061738
AUY56321.1	AUY58410.1	10	9	1,717864	83,1855	0,035696303	CpCAP3W_1137 Hypothetical protein	22498,9	4533,828	10981,96	15784,6	47585,11	1934,366	5,62419083
AUY56322.1	AUY58411.1	4	3	3,995686	38,6409	0,938016212	CpCAP3W_1138 Hypothetical protein	4283,633	5124,66	16911,53	3475,168	3111,892	0	1,717864265
AUY56325.1	AUY58414.1	5	4	1,319696	35,5643	0,278373226	CpCAP3W_1141 MutT/NUDIX family protein	14455,28	14610,93	19066,6	18019,92	8602,695	36898,05	0,250269919
AUY56327.1	AUY58416.1	10	9	2,332822	97,7611	0,799047343	CpCAP3W_1143 Hypothetical protein	15747,71	18526,75	14104,45	8323,555	4763,946	7650,858	1,319695693
AUY56282.1	AUY58417.1	19	15	2,122219	135,0043	0,010391754	CpCAP3W_1144 Inosine 5-monophosphate dehydrogenase	276,8355	214,2333	649,0004	390,1155	161,5952	1867,766	0,428665324
AUY56331.1	AUY58420.1	3	2	1,698855	16,4054	0,66174198	CpCAP3W_1147 Hypothetical protein	158,2359	172,3764	396,7505	911,72	323,9636	0	2,122219053
AUY56334.1	AUY58422.1	3	1	7555,101	21,6015	0,578080533	CpCAP3W_1150 Hypothetical protein	1,624693	0,898616	0	1,543081	0	19062,31	1,698854665
CpCAP3W_01161	CpCAPJ4_1159	8	8	1,885289	33,8054	0,389510181	CpCAP3W_1161 Hypothetical protein	7955,036	8609,174	5725,929	6527,783	5295,409	0	7555,101171
AUY56370.1	AUY58459.1	7	7	4,676601	40,7117	0,345259067	CpCAP3W_1190 Iron-sulfur cluster insertion protein erpA	4023,542	5044,235	8804,545	17043,45	11988,67	54549,6	0,530422558
AUY56380.1	AUY58469.1	25	17	1,139291	186,3463	0,055075633	CpCAP3W_1200 Cell-wall peptidase NlpC/P60 protein	15082,85	14751,1	12712,25	10879,85	12844,2	24748,43	4,676601049
AUY56388.1	AUY58477.1	3	3	1,918031	43,5806	0,806771589	CpCAP3W_1208 Transcription regulator	6262,481	31601,29	26529,71	18088,72	15483,99	0	1,139290736
AUY56410.1	AUY58499.1	4	1	1,419666	34,7606	0,373919954	CpCAP3W_1230 Copper oxidase	3940,09	5399,406	4507,865	3883,193	2127,499	3743,268	0,521368098
AUY56418.1	AUY58507.1	5	3	1,68634	59,4921	0,154282191	CpCAP3W_1238 Hypothetical protein	7992,758	9346,76	12944,75	18221,23	32125,33	723,0032	0,70439122
AUY56419.1	AUY58508.1	18	12	1,020396	106,2877	0,828312039	CpCAP3W_1239 Hypothetical protein	33238,5	23435,36	31252,85	50703,24	35458,49	7,432533	1,686339503
AUY56435.1	AUY58524.1	10	7	1,330779	96,4323	0,434955442	CpCAP3W_1255 Hypothetical protein	1857,176	3559,664	2810,117	3231,641	2910,206	40,21251	0,980011229
AUY56452.1	AUY58541.1	4	3	65,89777	34,3921	0,423455127	CpCAP3W_1272 Hypothetical protein	17,00574	17,57013	30,79037	162,3145	613,666	3531,508	0,751439337
AUY56456.1	AUY58545.1	3	3	1,4112	13,4257	0,018237097	CpCAP3W_1276 Galactose-1-phosphate uridylyltransferase	11828,49	9726,335	6921,861	10859,89	9319,161	0	65,89776869
AUY56457.1	AUY58546.1	2	1	1,577765	18,1114	0,385197418	CpCAP3W_1277 Hypothetical protein	43178,82	32404,06	28696,48	32212,8	30426,59	3453,683	0,708616571
AUY56461.1	AUY58550.1	29	23	4,913735	273,3899	0,330472128	CpCAP3W_1282 Hypothetical protein	4077,763	4911,056	5065,759	6558,426	5131,882	57370,17	0,633807838
AUY56462.1	AUY58551.1	7	6	1,565444	73,8344	0,271234356	CpCAP3W_1283 Metal-binding, possibly nucleic acid-binding protein	3581,391	1117,76	2776,919	2588,283	2158,034	29,37062	4,913735053
AUY56473.1	AUY58562.1	2	2	3,255189	15,7823	0,403466036	CpCAP3W_1295 Cupin	18506,14	17431,8	12776,16	8148,312	6816,751	0	0,638796438
AUY56482.1	AUY58571.1	9	7	1,354442	74,8151	0,283345379	CpCAP3W_1304 Hypothetical protein	2084,192	2753,425	2211,94	3281,505	2734,987	3531,721	0,307201843
AUY56484.1	AUY58573.1	5	2	1,569402	23,6718	0,055158715	CpCAP3W_1306 Rossmann-fold nucleotide-binding protein/SMF	14795,64	12739,98	11170,66	10672,18	13990,89	0	1,35444165
AUY56493.1	AUY58581.1	6	6	3,206357	49,4807	0,368095424	CpCAP3W_1315 Hypothetical protein	6941,062	6679,066	4385,07	2427,666	3187,803	0	0,637185501
AUY56494.1	AUY58582.1	5	4	1,362837	49,2491	0,27674501	CpCAP3W_1317 Hypothetical protein	13676,97	18361,35	13493,79	15845,34	14506,66	31700,84	0,311880437
AUY56503.1	AUY58591.1	31	21	6,072495	220,2117	0,393009964	CpCAP3W_1326 Mycothione/glutathione reductase	2997,99	415,7638	210,2244	3,853637	36,49132	556,4408	1,362836797
AUY56515.1	AUY58603.1	3	2	1,108366	18,8287	0,174992296	CpCAP3W_1338 Hypothetical protein	45197,94	36069,4	35277,49	31387,99	25101,79	48660,35	0,16467695
AUY56530.1	AUY58618.1	13	9	2,237162	123,7364	0,554675165	CpCAP3W_1353 SSU ribosomal protein S15p (S13e)	121979,6	62895	68952,6	169798,3	142152,3	255901,9	0,902229079
AUY56535.1	AUY58623.1	54	41	3,054633	419,6807	0,038884225	CpCAP3W_1358 Ribonuclease j Rv2752c	1200,82	2116,757	1607,154	898,3831	713,8337	0	2,237162136
AUY56536.1	AUY58624.1	7	5	2,715401	38,7625	0,270453618	CpCAP3W_1359 Hypothetical protein	6485,295	17101,21	18305,74	34079,49	23202,06	56472,65	0,327371533
AUY56539.1	AUY58627.1	8	6	74,16714	69,9512	0,071399108	CpCAP3W_1362 Hypothetical protein	13,48455	238,9958	124,3319	48,95419	116,2553	27781,88	2,715400567
AUY56547.1	AUY58635.1	2	2	5,969366	19,2988	0,410031674	CpCAP3W_1370 Hypothetical protein	5546,543	11895,51	7172,578	18566,74	26729,27	101637,7	74,16713559
AUY56552.1	AUY58640.1	48	41	1,088839	388,4654	0,050695373	CpCAP3W_1376 Hypothetical protein	1010,729	982,073	726,7119	1483,783	1013,843	0	5,969365724
AUY56553.1	AUY58641.1	4	3	1,573762	27,1622	0,429437037	CpCAP3W_1377 Hypothetical protein	6328,567	5741,356	5932,535	5604,067	5559,39	275,6696	0,918409231
AUY56557.1	AUY58645.1	6	5	1,165155	32,8045	0,344508743	CpCAP3W_1381 Hypothetical protein	40828,6	40802,93	55102,06	61991	42113,41	13247,82	0,635420272
AUY56562.1	AUY58650.1	5	5	2,049899	46,4678	0,53036122	CpCAP3W_1386 Phosphocarrier protein of PTS system	12864,71	5349,613	3281,407	2631,597	2801,459	5053,18	0,858254643
AUY56585.1	AUY58674.1	2	2	1,89851	12,0779	0,253268903	CpCAP3W_1410 Hypothetical protein	3798,447	5237,117	4680,363	4929,343	2295,231	0	0,487828897
AUY56586.1	AUY58675.1	6	5	23,93018	41,6317	0,333915756	CpCAP3W_1411 Hypothetical protein	551,1516	307,3072	266,5502	279,1163	160,857	26481,7	0,526728791
AUY56587.1	AUY58676.1	4	3	48,3978	22,12	0,540752984	CpCAP3W_1412 Type III restriction endonuclease, res subunit	62,78182	412,2702	212,4963	1251,777	855,5065	31168,54	23,93018216
AUY56591.1	AUY58680.1	5	3	2,302181	38,6233	0,083624044	CpCAP3W_1417 Hypothetical protein	898,6536	1222,527	783,6318	596,4878	665,2777	0	48,39779531
AUY56593.1	AUY58682.1	2	2	1,71867	10,4904	0,306476915	CpCAP3W_1419 Hypothetical protein	18860,04	32693,78	26332,05	29784,68	23954,42	80120,97	0,434370772
AUY56595.1	AUY58684.1	21	19	1,38076	152,7354	0,358226405	CpCAP3W_1421 Hypothetical protein	1172,714	3138,134	2552,421	2786,935	2183,712	0	1,7186695
AUY56600.1	AUY58689.1	4	3	2,283096	22,1071	0,400743883	CpCAP3W_1426 Hypothetical protein	84643,3	92316,83	85261,81	49458,23	65310,77	84,66296	0,724239066
AUY56601.1	AUY58690.1	28	19	1,730794	278,2888	0,297359079	CpCAP3W_1427 Chlorite dismutase	83779,73	84244,04	48837,74	67153,61	56778,86	1363,502	0,438001718
AUY56608.1	AUY58696.1	32	24	1,613787	292,8545	0,337670877	CpCAP3W_1434 Lipoprotein	1540,383	741,1746	763,7658	923,0655	828,2653	3163,173	0,577769525
AUY56611.1	AUY58699.1	6	3	1,449194	32,4518	0,527300005	CpCAP3W_1437 Hit (histidine triad) family protein	16327,51	16690,95	15255,08	18147,4	15031,67	131,5428	1,613787446
AUY56621.1	AUY58709.1	18	14	1,145153	155,8163	0,381407847	CpCAP3W_1447 Transcriptional regulator	127,3338	106,182	195,6571	191,6688	299,7997	0	0,69003878
AUY56641.1	AUY58729.1	3	2	1,12004	15,7887	0,50669558	CpCAP3W_1467 NADPH-dependent FMN reductase	2973,737	3426,87	3550,138	3832,261	5041,376	10,64211	1,145152705
AUY56643.1	AUY58731.1	4	3	3,301691	20,8106	0,439598029	CpCAP3W_1469 Hypothetical protein	502,0088	199,885	284,9447	505,51	696,4455	2056,28	0,89282544
AUY56644.1	AUY58732.1	3	3	1,795636	13,5711	0,098494447	CpCAP3W_1470 Hypothetical protein	1148,716	618,962	558,1869	522,7618	772,5256	0	3,301690859
AUY56645.1	AUY58733.1	3	2	1,371937	15,2158	0,350887969	CpCAP3W_1471 SNF2 family DNA/RNA helicase	11876,93	5726,913	6808,266	7690,583	10103,31	0	0,556905798
AUY56648.1	AUY58736.1	17	11	711,4641	103,8043	0,392531823	CpCAP3W_1474 Phosphotransferase enzyme family protein	20,90396	4,094321	0	0	3,92355	17781,46	0,728896264
AUY56651.1	AUY58739.1	2	1	2,147929	9,89	0,544774907	CpCAP3W_1477 Holliday junction resolvase	41142,29	48912,76	50674,2	32926,72	31653,03	938,8391	711,4641289
AUY56659.1	AUY58747.1	37	25	1,383195	321,5989	0,259926757	CpCAP3W_1485 Peptidase YqhT	970,8463	387,2397	1193,811	2346,579	1183,193	0	0,465564805
AUY56664.1	AUY58752.1	7	2	1,096889	55,6589	0,519819023	CpCAP3W_1490 Epimerase family protein yfcH	18883,8	12193,86	17397,62	32706,36	20113,3	352,3526	1,383195424
AUY56688.1	AUY58776.1	35	23	1,433135	289,4725	0,546135672	CpCAP3W_1514 P-loop ATPase protein family	18705,26	21406,06	17763,72	18368,9	22014,62	0	1,096889047
AUY56697.1	AUY58785.1	22	18	11,25715	172,4484	0,379458	CpCAP3W_1523 OxPP cycle protein opcA	719,9749	1509,264	1783,74	5483,348	3572,801	36118,55	0,697770986
AUY56712.1	AUY58800.1	4	3	1,998042	39,8289	0,061556071	CpCAP3W_1538 Hypothetical protein	6986,118	7606,438	6367,999	4191,678	3250,394	3048,475	11,25714818
AUY56713.1	AUY58801.1	11	6	6,097354	67,807	0,003133907	CpCAP3W_1539 ABC transporter ATP-binding protein	66743,63	26653,94	60135,69	146139,4	168020,4	621986,8	0,50048994
AUY56717.1	AUY58806.1	28	22	9,387522	219,7907	0,038607921	CpCAP3W_1543 Hypothetical protein	1209,952	2416,71	2861,688	6256,011	4956,266	49697,25	6,097353786
AUY56718.1	AUY58807.1	3	2	1,239686	35,3965	0,089314924	CpCAP3W_1544 ACT domain protein	25121,35	14672,45	18277,46	23479,58	22590,91	773,0531	9,387521951
AUY56723.1	AUY58812.1	37	28	1,096689	249,3198	0,459855523	CpCAP3W_1549 Invasion-associated protein p60	1172,067	960,0141	964,6967	767,672	794,933	1833,597	0,806656127
AUY56726.1	AUY58815.1	11	5	1,004875	70,6419	0,974011654	CpCAP3W_1552 Hypothetical protein	8597,416	21138,47	19684,35	25090,75	23150,46	1419,929	1,096689216
AUY56728.1	AUY58817.1	20	15	1,113829	128,2318	0,646323332	CpCAP3W_1554 Hypothetical protein	9275,065	9831,529	12861,16	16250,62	12450,17	0	1,004874816
AUY56733.1	AUY58822.1	22	15	2,601311	126,3067	0,413432948	CpCAP3W_1559 Arginine/ornithine transport system ATPase	13815,74	13439,76	14096,5	7099,345	8208,344	588,9133	0,897804237
AUY56742.1	AUY58831.1	5	4	1,297164	31,838	0,166396975	CpCAP3W_1568 Phosphatidylethanolamine-binding protein	5059,114	4199,192	4868,59	5421,979	5468,618	0	0,384421534
AUY56747.1	AUY58836.1	4	3	1,785547	21,3979	0,39503717	CpCAP3W_1573 Aldo/keto reductase family oxidoreductase	3992,562	3419,91	2995,053	3097,256	2731,506	0	0,77091227
AUY56750.1	AUY58839.1	3	3	1,081879	27,4719	0,349685268	CpCAP3W_1576 Hypothetical protein	3698,186	1041,1	1330,498	1311,825	761,927	4493,018	0,560052603
AUY56753.1	AUY58842.1	8	6	1,084583	71,1252	0,94963858	CpCAP3W_1579 Phosphoribosyl-ATP pyrophosphatase	30269,29	14721,89	15690,74	12861,31	13489,83	29598,39	1,081878727
AUY56785.1	AUY58873.1	8	5	1,240437	54,4649	0,786415964	CpCAP3W_1611 Hypothetical protein	16801,1	28570,24	23473,74	25195,53	23024,94	7280,197	0,922012996
AUY56794.1	AUY58882.1	18	12	1,16599	140,2081	0,48794847	CpCAP3W_1620 Thioesterase	18398,43	16535,96	19862,23	41703,57	21904,36	284,3604	0,806167664
AUY56800.1	AUY58888.1	14	9	3,464896	99,9724	0,539980952	CpCAP3W_1626 Hypothetical protein	12076,86	13708,37	9666,435	5509,415	4673,279	48,97228	1,165989656
AUY56807.1	AUY58895.1	21	13	2,579491	142,5204	0,199378197	CpCAP3W_1633 Hydrolase alpha/beta superfamily	489,7467	3512,289	2232,414	1641,08	775,8493	0	0,288608925
AUY56810.1	AUY58897.1	3	1	1,7521	15,9785	0,335170131	CpCAP3W_1636 Hypothetical protein	30384,52	27161,05	22641,6	20390,24	25376,09	0	0,38767332
AUY56838.1	AUY58923.1	28	22	1,823969	212,9012	0,355985372	CpCAP3W_1664 Hypothetical protein	13091,93	15081,65	13353,75	9747,003	8478,48	4542,078	0,570743776

AUY56839.1	AUY58924.1	13	11	4,215688	102,256	0,052984971	CpCAP3W_1665 Thiamin pyrophosphokinase, catalytic domain-containing protein	12985,47	14613,51	11492,34	4657,949	4585,805	29,06736	0,548254863
AUY56845.1	AUY58930.1	19	14	1,400462	132,1262	0,182838068	CpCAP3W_1671 Hypothetical protein	7913,829	3371,492	5009,62	10668,05	5405,217	6747,174	0,237209201
AUY56848.1	AUY58934.1	6	4	1,743585	63,0451	0,324862463	CpCAP3W_1674 Protein YcaR in KDO2-Lipid A biosynthesis cluster	13984,51	10231,65	6342,154	7456,734	6201,564	3867,842	1,400461686
AUY56860.1	AUY58946.1	8	5	1,736003	74,7463	0,147298591	CpCAP3W_1686 Hypothetical protein	3979,716	7061,505	5419,811	6145,092	3337,051	0	0,573531017
AUY56865.1	AUY58951.1	4	3	1,379691	39,2828	0,35256812	CpCAP3W_1691 Metallo-beta-lactamase superfamily protein	3024,45	5846,455	7208,274	5944,115	5710,075	0	0,576035801
AUY56874.1	AUY58960.1	3	2	3,154244	22,3945	0,396016025	CpCAP3W_1700 SAM-dependent methyltransferase	3134,18	1541,725	3473,747	1758,115	1185,534	22762,35	0,724800041
AUY56884.1	AUY58969.1	4	4	3,003668	28,4237	0,736736174	CpCAP3W_1710 Pyruvate carboxylase, C-terminal domain/subunit	5927,598	4828,447	4313,088	2539,362	2477,548	0	3,154244266
AUY56889.1	AUY58974.1	5	3	53,03564	34,7845	0,282497299	CpCAP3W_1715 Hypothetical protein	1005,194	944,6958	76,00999	13230,5	6972,789	87241,57	0,332926276
AUY56898.1	AUY58983.1	25	21	1,042658	190,0321	0,027286444	CpCAP3W_1724 Conserved hypothetical exported protein	5203,345	6818,475	6555,272	7001,245	9230,411	1585,393	53,03563724
AUY56911.1	AUY58996.1	42	27	2,290896	251,1215	0,647380882	CpCAP3W_1737 Hypothetical protein	13773,1	11154,58	8055,734	6646,555	7751,05	0	0,959087121
AUY56917.1	AUY59002.1	24	17	1416,54	173,3333	0,323618331	CpCAP3W_1745 Hypothetical protein	3,676089	2,311656	2,981171	10,74625	0,399351	12693,68	0,436510478
AUY56918.1	AUY59003.1	4	4	1,377109	19,7312	0,400961356	CpCAP3W_1746 Hypothetical protein	28714,6	24616,12	27843,43	43160,43	24558,03	44067,16	1416,540108
AUY56933.1	AUY59017.1	5	5	2,672028	62,0454	0,217864126	CpCAP3W_1761 Aspartyl-tRNA(Asn) amidotransferase subunit C	23175,54	7219,566	10312,7	19486,19	73986,04	15300,16	1,377108721
AUY56934.1	AUY59018.1	5	5	1,457588	49,7836	0,229373525	CpCAP3W_1762 Hypothetical protein	575,8978	14706,59	10503,15	16592,77	19429,69	1562,369	2,672028069
AUY56937.1	AUY59021.1	5	4	1,955532	35,973	0,682419554	CpCAP3W_1765 Hypothetical protein	3532,176	4873,454	5077,074	10366,26	9270,649	6728,946	1,457587992
AUY56949.1	AUY59033.1	8	7	1,151116	72,8623	0,01821162	CpCAP3W_1777 Hypothetical protein	110339,3	101920	83781,66	86133,57	82689,02	88354,75	1,955531563
AUY56950.1	AUY59034.1	12	10	1,432508	181,5567	0,18479002	CpCAP3W_1778 Methylmalonyl-CoA epimerase	3117,901	5048,09	4149,722	1503,566	1355,954	14782,84	0,868722301
AUY56952.1	AUY59036.1	2	2	1,02977	10,457	0,761149537	CpCAP3W_1780 Hypothetical protein	2549,567	2262,714	2414,542	2468,288	3773,746	1199,928	1,432507975
AUY56969.1	AUY59053.1	7	4	1,251035	41,03	0,837912986	CpCAP3W_1797 Hypothetical protein	20830,51	30560,91	34148,9	37235,02	30480,64	659,9824	1,029769544
AUY56977.1	AUY59061.1	14	10	1,868383	130,5692	0,44317156	CpCAP3W_1805 Hypothetical protein	2319,99	4309,039	8014,916	7099,431	5752,399	14508,67	0,799338217
AUY56991.1	AUY59075.1	12	10	14,23077	89,5004	0,216077568	CpCAP3W_1820 Glyoxalase/Bleomycin resistance protein/Dihydroxybiphenyl dioxygenase	7334,744	6434,313	7782,324	17843,96	7258,453	281590,3	1,868338029
AUY57000.1	AUY59084.1	13	9	4,078899	101,3022	0,236651236	CpCAP3W_1829 Hypothetical protein	2132,169	2816,884	2657,019	3767,872	3484,642	23771,88	14,23076719
AUY57004.1	AUY59088.1	19	12	1,105967	116,3389	0,192532669	CpCAP3W_1833 Hypothetical protein	5606,556	5345,592	4794,43	3856,773	4369,768	6011,293	4,078898938
AUY57009.1	AUY59093.1	4	4	2,223974	23,828	0,452189608	CpCAP3W_1838 Hypothetical protein	1613,051	12415,13	5537,661	6461,597	25859,11	11193,23	0,904185932
AUY57014.1	AUY59098.1	7	5	1,810231	64,5571	0,259534135	CpCAP3W_1843 O-methyltransferase	21884,28	31115,93	45667,62	55883,44	36428,99	86299,15	2,223974475
AUY57020.1	AUY59104.1	15	10	6,084761	152,4937	0,152570486	CpCAP3W_1849 Hypothetical protein	11512,48	4890,06	4859,604	1667,746	1826,582	0	1,81023123
AUY57021.1	AUY59105.1	4	2	1,867996	35,0146	0,214280393	CpCAP3W_1850 Hypothetical protein	6316,695	5122,25	5608,067	2606,671	1866,826	4652,332	0,164344981
AUY57024.1	AUY59108.1	11	8	4,328467	71,9056	0,064254298	CpCAP3W_1853 Lysine decarboxylase	14505,51	10044,43	13034,06	30683,93	18624,56	113372,6	0,535333027
AUY57033.1	AUY59117.1	5	3	6,692304	58,999	0,098931052	CpCAP3W_1862 4Fe-4S ferredoxin, iron-sulfur binding	4625,396	0,980472	16778,95	21708,19	36893,93	151434,6	4,328467326
AUY57038.1	AUY59122.1	10	9	8,19238	108,7481	0,07111034	CpCAP3W_1867 Hypothetical protein	376,3445	2224,249	1332,507	2071,463	1109,521	29040,48	6,69230396
AUY57039.1	AUY59123.1	12	9	1,521814	75,0819	0,294421974	CpCAP3W_1868 Hypothetical protein	7234,279	7218,68	6243,662	6387,916	6963,713	248,3385	8,192380332
AUY57057.1	AUY59141.1	3	2	4,641174	14,3353	0,362295237	CpCAP3W_1886 Hypothetical protein	1795,145	3253,604	4303,337	8965,588	4856,853	29582,21	0,657110521
AUY57063.1	AUY59148.1	4	4	1,843552	41,2343	0,090121793	CpCAP3W_1892 Exodeoxyribonuclease VII small subunit	1702,142	1918,45	4955,89	4207,307	9362,83	2241,048	4,64117393
AUY57064.1	AUY59149.1	7	6	1,105784	38,4235	0,349528325	CpCAP3W_1893 Hypothetical protein	3087,524	3881,049	3426,313	5781,264	5713,237	0	1,843551507
AUY57070.1	AUY59155.1	7	6	1,825705	62,1695	0,450163542	CpCAP3W_1899 Hypothetical protein	712,415	3240,71	1897,361	2759,326	2404,441	5517,496	1,105784217
AUY57072.1	AUY59157.1	6	5	2,356049	42,2994	0,239549864	CpCAP3W_1901 Hypothetical protein	9244,605	5381,118	2427,557	8957,015	8239,058	22982,28	1,825705164
AUY57087.1	AUY59172.1	16	11	13,3969	105,8162	0,158558787	CpCAP3W_1916 Hypothetical protein	4292,713	5574,405	4089,791	4177,698	2731,711	180069,9	2,356048771
AUY57106.1	AUY59191.1	22	16	1,00477	161,4504	0,489336326	CpCAP3W_1935 Short-chain dehydrogenase	1818,75	2562,767	1510,025	2309,361	2285,414	1268,796	13,39689989
AUY57112.1	AUY59198.1	3	3	1,84106	16,8938	0,950534213	CpCAP3W_1941 Hypothetical protein	81527,12	70467,18	83810,22	95860,45	63629,87	274640	0,995252354
AUY57117.1	AUY59203.1	15	12	1,231889	169,3971	0,397245055	CpCAP3W_1947 antibiotic biosynthesis monooxygenase	1276,539	927,7894	1635,902	1531,518	2993,534	205,6844	1,841060051
AUY57135.1	AUY59221.1	3	2	1,222649	17,2056	0,784573706	CpCAP3W_1965 5-formyltetrahydrofolate cyclo-ligase	25687,36	29142,91	26293,54	37248,36	29075,29	27,19896	1,231888876
AUY57136.1	AUY59222.1	2	1	1,235439	16,0398	0,411581025	CpCAP3W_1966 Hypothetical protein	21823,19	55891,25	31958,25	48886,07	48556,06	38051,79	0,817896127
AUY57144.1	AUY59229.1	3	3	2,69502	47,7649	0,381698895	CpCAP3W_1974 LSU ribosomal protein L31p	9898,374	13124,05	11472,41	11091,78	8779,791	73092,69	1,235439077
AUY57145.1	AUY59230.1	12	11	1,688158	124,2213	0,483539724	CpCAP3W_1975 LSU ribosomal protein L28p	2283,788	6153,909	2927,28	3469,972	2555,29	706,9155	2,695019797
AUY57147.1	AUY59232.1	4	4	1,135053	40,6569	0,333843863	CpCAP3W_1977 SSU ribosomal protein S14p (S29e)	19118,58	17754,96	13965,37	13590,84	13868,16	17330,9	0,592361784
AUY57148.1	AUY59233.1	5	4	66,46122	48,0458	0,368849387	CpCAP3W_1978 SSU ribosomal protein S18p	438,8953	704,7855	550,7687	870,7769	873,9867	110870,4	0,881016017
AUY57149.1	AUY59234.1	16	13	1,020696	99,8589	0,270954684	CpCAP3W_1979 Nucleotidyltransferase substrate binding domain protein	1911,278	5557,897	8087,13	9425,829	5815,054	0	66,46122224
AUY57172.1	Absent	7	5	1,649804	49,2332	0,453197	CpCAP3W_2002 Glutaredoxin	11347,46	24754,1	16845,91	45707,92	23052,12	18592,92	0,979723808
AUY57188.1	AUY59271.1	9	7	1,652365	84,0146	0,250265036	CpCAP3W_2018 Hypothetical protein	24631,86	13653,43	17349,84	11095,73	22060,09	514,1684	1,64980427
AUY57200.1	AUY59282.1	19	11	5,297328	123,2532	0,335157981	CpCAP3W_2031 Hypothetical protein	188,9318	252,171	217,6966	333,7575	462,6735	2693,446	0,605193073
AUY57212.1	AUY59294.1	2	2	151,8256	8,4808	0,132988923	CpCAP3W_2043 Hypothetical protein	1220,602	904,0042	42,24641	422,2228	360,3178	328201,1	5,29732803
AUY57215.1	AUY59298.1	2	2	1,400034	18,0956	0,403115671	CpCAP3W_2047 GntR family transcriptional regulator	4463,996	3360,781	2917,784	3803,984	3868,615	0,473679	151,8255718
AUY57216.1	AUY59299.1	8	6	17,65171	49,1267	0,387380268	CpCAP3W_2048 Hypothetical protein	903,4796	2176,644	4014,262	3930,781	785,9651	120511,3	0,7142684
AUY57220.1	AUY59303.1	3	3	1,408213	34,3129	0,452683938	CpCAP3W_2052 Copper chaperone	6314,625	13876,31	8638,12	8334,41	6623,801	5513,879	17,6517055
AUY57238.1	AUY59320.1	19	10	1,218466	104,45	0,30477915	CpCAP3W_2070 Hypothetical protein	30471,91	19940,11	23074,63	36938,33	22041,85	1330,593	0,710119944
AUY57244.1	AUY59326.1	48	31	12,79675	341,8092	0,457971734	CpCAP3W_2076 ABC transporter	922,3378	1692,217	1612,291	3241,14	3763,755	47084,98	0,820703768
AUY57248.1	AUY59330.1	4	3	1,33561	22,7651	0,11164337	CpCAP3W_2080 antimicrobial peptide ABC transporter ATPase	527,5044	1300,323	1870,218	1224,325	1544,481	0	12,79674551
AUY57260.1	AUY59342.1	7	2	47,57339	36,6448	0,418350138	CpCAP3W_2092 GntR family transcriptional regulator	122,8742	21,3032	26,12652	1345,002	1856,656	4900,274	0,748721651
AUY57271.1	AUY59352.1	8	5	1,043111	47,3456	0,003968702	CpCAP3W_2103 Hypothetical protein	542608	39926,69	48436,15	148177,7	451067,2	5648,452	47,57338952
AUY57272.1	AUY59353.1	42	29	4,962474	265,7585	0,837743446	CpCAP3W_2104 Hypothetical protein	1585,611	174,9218	106,8034	156,3074	219,9839	0	0,958670823
AUY57273.1	AUY59354.1	2	1	1,180461	23,2611	0,310606211	CpCAP3W_2105 Hypothetical protein	5400,773	3061,53	5472,156	10624,41	5824,677	0	0,201512387
CpCAP3W_02106	CpCAPJ4_2097	3	2	1,686313	20,3617	0,45663021	CpCAP3W_2106 Hypothetical protein	1134,423	3276,542	2801,98	2126,856	2437,088	7599,338	1,180461251
AUY57274.1	AUY59355.1	6	4	2,051104	38,3848	0,443293288	CpCAP3W_2107 UDP-N-acetylglucosamine diphosphorylase	7898,339	4871,35	7836,442	4521,534	5504,596	20,23032	1,686312893
AUY57291.1	AUY59372.1	9	5	3,853685	48,5451	0,311964442	CpCAP3W_2124 Hypothetical protein	7241,156	742,7429	864,2177	1219,338	1076,676	0	0,487542324
AUY57689.1	AUY57689.1	4	2	11,84853	18,9194	0,332377348	CpCAPJ4_0389 Hypothetical protein	2964,493	1856,42	941,5105	318,7122	167,6287	0	0,259491872
AUY55621.1	AUY57710.1	3	1	1,610446	16,0558	0,122868037	CpCAPJ4_0410 Hypothetical protein	11911,28	12392,87	10550,1	12132,17	9510,44	0	0,084398672
AUY57913.1	AUY57913.1	4	1	5934,335	22,5468	0,364325592	CpCAPJ4_0619 Hypothetical protein	0	2,543786	2,688804	0	1,134383	31050,81	0,62094613
CpCAP3W_00775	CpCAPJ4_00776	8	5	8,31982	51,9438	0,465770274	CpCAPJ4_0776 Disulfide bond formation protein B	30208,01	12633,05	9446,23	2864,054	3420,613	0	5934,334826
AUY56393.1	AUY58482.1	2	1	3,11459	18,6398	0,199793582	CpCAPJ4_1211 Hypothetical protein	609,5539	182,2235	98,99568	1654,161	1120,232	0	0,12019491
AUY56716.1	AUY58805.1	2	2	1,214744	11,4077	0,774667225	CpCAPJ4_1540 Hypothetical protein	16987,38	18716,06	51042,71	56581,15	14829,88	0	3,11458981
AUY57174.1	AUY59257.1	6	4	4,220696	57,3954	0,394343871	CpCAPJ4_1997 Hypothetical protein	175,871	376,9009	465,6253	1392,719	512,4787	2393,147	0,823218446
AUY57294.1	AUY59375.1	6	2	4,994703	37,4992	0,068264184	CpCAPJ4_2118 Hypothetical protein	11610,04	12018,69	10192,81	9670,96	7648,009	151609,6	4,220695544
AUY56710.1	AUY58798.1	27	17	1,384823	164,9377	0,513119714	csdA Cysteine desulfurase (SufS)-like protein	4804,992	4121,652	4196,077	4351,238	3985,293	9836,118	4,994703012
AUY57161.1	AUY59246.1	4	4	3,57767	45,764	0,457367412	csm Chorismate mutase	3126,895	3048,685	2154,725	2439,914	1868,527	25494,64	1,384823242
AUY57207.1	AUY59289.1	6	4	1,655707	40,3491	0,528878482	cspB Cold-shock protein	13060,07	8855,719	10534,44	8663,098	6918,827	38146,14	3,577669815
AUY56372.1	AUY58461.1	11	9	3,193521	99,0625	0,7208933	ctaC Cytochrome c oxidase subunit II	6358,967	25087,21	20452,32	6906,335	9344,849	0	1,655706927
AUY56139.1	AUY58228.1	4	1	2,618735	39,2052	0,303663524	ctaD Cytochrome C oxidase polypeptide I	4583,95	2205,032	2446,753	7294,39	7331,996	9559,555	0,313133966
AUY57300.1	AUY59381.1	31	22	2,100299	200,0788	0,014684101	cwlM N-Acetymuramyl-L-Alanine Amidase	1410,494	868,0301	961,2501	1253,01	1169,474	4382,008	2,618735048
AUY55790.1	AUY57881.1	15	8	1,718062	94,0191	0,280845281	cynT carbonic anhydrase	39702,37	27841,49	25327,9	26802,54	34654,76	98102,2	2,100298511
AUY55688.1	AUY57777.1	35	23	1,050995	297,5665	0,401599162	cysA Thiosulfate sulfurtransferase	56761,34	72371,56	51950,28	98522,29	91153,4	641,9245	1,718062486
AUY56109.1	AUY58198.1	38	32	1,443932	388,8595	0,510408403	cysK Cysteine synthase	31653,71	33202,71	28611,45	27167,78	24787,54	12776,16	1,050995494
AUY57169.1	AUY59253.1	17	11	6,618031	139,4731	0,159466948	cysQ 3'-phosphoadenosine 5'-phosphate phosphatase	500,1697	1232,129	1022,778	1925,706	1433,37	14874,11	0,692553241
AUY56051.1	AUY58142.1	10	7	2,322653	61,7052	0,150933244	cysS cysteinyl-tRNA synthetase	39672,84	37260,63	29870,46	19481,06	26502,15	0,381197	6,618031236
AUY56534.1	AUY58622.1	17	14	2,15642	168,7874	0,322652918	dapA Dihydrodipicolinate synthase	3629,115	3129,263	2636,473	2170,86	2185,828	0	0,430542053
AUY56173.1	AUY58262.1	4	2	19,57171	31,2055	0,32303368	dapA1 Dihydrodipicolinate synthase	2509,318	2426,227	1771,655	2076,581	2216,856	126977,5	0,463731504
AUY56532.1	AUY58620.1	15	11	1,725082	98,0431	0,384724446	dapB Dihydrodipicolinate reductase	10739,53	9091,201	9631,588	5436,051	7834,442	37554,44	19,57170924
AUY57032.1	AUY59116.1	20	15	1,573869	136,6762	0,780807298	dapC N-succinyldiaminopimelate aminotransferase	41318,24	26589,11	24060,06	17651,77	18865,6	21916,61	1,725082432
AUY57028.1	AUY59112.1	30	21	4,15052	230,0942	0,07357577	dapD 2,3,4,5-tetrahydropyridine-2,6-dicarboxylate N-succinyltransferase	1887,908	1498,009	1233,562	537,1466	565,226	10,61536	0,635377063
AUY57026.1	AUY59110.1	4	4	1,041032	30,3847	0,152905608	dapD1 2,3,4,5-tetrahydropyridine-2,6-dicarboxylate N-succinyltransferase	2088,586	3510,561	3645,879	3974,897	3705,721	1200,019	0,240933646

AUY57025.1	AUY59109.1	8	5	1,157582	47,8635	0,763291186	dapE Succinyl-diaminopimelate desuccinylase	3876,92	6405,348	5491,738	5232,281	5186,352	7841,069	0,960585418
AUY56555.1	AUY58643.1	15	11	1,840622	107,3509	0,502838208	dapF Diaminopimelate epimerase	11214,65	11832,89	7727,274	5091,539	4350,453	47202,82	1,1575818
AUY55883.1	AUY57977.1	7	7	1,137734	67,5948	0,993776282	dcd Deoxycytidine triphosphate deaminase	1787,469	2356,16	1988,422	3233,493	2156,21	0	1,840622319
AUY56267.1	AUY58354.1	12	5	1,812398	73,2776	0,415711157	dcp Peptidyl-dipeptidase	3384,376	7170,04	5685,067	4519,819	4427,381	13,02011	0,878939754
AUY56755.1	AUY58844.1	7	6	1,047812	57,3966	0,346025249	dcuA Anaerobic C4-dicarboxylate transporter	14067,29	19461,13	18664,04	19480,24	19269,82	11060,84	0,551755246
AUY56890.1	AUY58975.1	24	21	2,632011	151,843	0,761504913	ddl D-alanyl-alanine synthetase A	17483,25	22438,51	49153,91	18277,43	14937,03	628,7328	0,954369565
AUY56993.1	AUY59077.1	57	42	1,091766	438,6044	0,237733898	deaD1 DEAD/DEAH box helicase	31746,44	17098,77	30385,94	22064,7	64435,72	1,458751	0,379937606
AUY56678.1	AUY58766.1	10	8	1,152863	77,8154	0,429735157	def1 Peptide deformylase 1	8649,12	13843,19	12466,36	6452,976	6614,664	17255,72	1,091765982
AUY55823.1	AUY57915.1	35	29	3,06653	261,4146	0,54218594	deoA Thymidine phosphorylase	65820,2	82532,88	82861,34	40834,92	24944,75	9619,686	0,867406032
AUY55377.1	AUY57467.1	24	15	4,02122	236,1334	0,04158188	deoC1 Deoxyribose-phosphate aldolase	2646,768	2473,7	2025,799	879,7288	897,41	0	0,326101445
AUY55463.1	AUY57552.1	5	4	2,348697	37,2713	0,236289587	deoC2 Deoxyribose-phosphate aldolase	7178,352	39524,35	46047,27	35114,33	4352,457	23,1834	0,248680733
AUY55375.1	AUY57465.1	9	6	1,408454	87,51	0,292083688	deoD Purine nucleoside phosphorylase	5736,247	4518,588	4017,299	4739,248	5393,945	0	0,425767985
AUY55374.1	AUY57464.1	13	9	1,378375	83,0029	0,384361933	deoR Deoxyribonucleoside regulator	5085,821	7867,307	3787,517	3280,79	5069,766	14724,32	0,709998482
AUY55735.1	AUY57826.1	29	21	14,12156	193,2199	0,762236856	desA3 Stearoyl-CoA 9-desaturase	668,3043	436,8367	432,6727	82,81768	79,78836	21553,72	1,378374561
AUY55366.1	AUY57456.1	4	3	1,625283	27,6644	0,983906396	dgoR Galactonate operon transcriptional repressor	121,2758	35,07151	22196,92	81,54904	171,2279	36077,6	14,12155533
AUY56738.1	AUY58827.1	9	9	2,71727	81,3696	0,849328638	dhaK Dihydroxyacetone kinase subununit	58787,26	32751,37	36713,04	20723,92	26374,15	100,6524	1,625282556
AUY56739.1	AUY58828.1	28	17	1,65326	250,8833	0,261382277	dhaL Dihydroxyacetone kinase family protein	3473,094	2362,753	2370,858	2709,038	2253,035	1,879533	0,368016417
AUY56416.1	AUY58505.1	10	4	1,467441	55,2677	0,359532441	dinB DNA polymerase IV	67505,44	40060,94	35953,02	41834,35	40112	15856,12	0,604865499
AUY57193.1	AUY59275.1	34	29	1,821287	327,8056	0,309664276	dkgA 2,5-diketo-D-gluconic acid reductase A	15013,9	21349,17	49841,2	31614,5	15717,02	0	0,681458211
AUY56424.1	AUY58513.1	4	4	3,353482	14,2056	0,36226442	dnaE DNA polymerase III subunit alpha	2379,164	2674,408	2348,604	354,4624	339,6649	24128,94	0,549062407
AUY55916.1	AUY58010.1	16	11	6,29117	98,2373	0,719196252	dnaJ Chaperone protein cofactor 1	4391,467	4677,439	3591,991	3304,135	2512,199	73835,52	3,353482202
AUY56276.1	AUY58363.1	22	16	1,895218	180,4987	0,551611615	dnaJ1 Chaperone protein dnaJ 1	384183,5	215540,1	249556,1	235571,3	211285,9	1259,922	6,291169605
AUY55914.1	AUY58008.1	93	64	2,327902	1017,0972	0,32762977	dnaK Chaperone protein DnaK	20074,89	23106,57	17108,7	14039,87	11828,91	30,15066	0,527643755
AUY55218.1	AUY57309.1	19	15	2,287623	140,8315	0,291227558	dnaN DNA polymerase III subunit beta	2131,93	2482,698	2103,872	1510,467	1426,424	0	0,429571418
AUY55372.1	AUY57462.1	4	2	2,05573	21,5049	0,311517093	dnaQ1 DNA polymerase III subunit epsilon	872,8894	248,4561	221,1846	79,19634	126,2759	447,5952	0,437135034
AUY56936.1	AUY59020.1	5	4	1,599354	24,8888	0,308917057	dnaQ2 DNA polymerase III subunit epsilon	24251,63	26028,7	18067,9	24867,9	17451,46	415,5323	0,486445239
AUY56866.1	AUY58952.1	20	15	2,886969	170,6091	0,352038855	doxX DoxX family protein	4630,863	8541,12	9498,706	9296,372	6914,8	49238,41	0,62525236
AUY55321.1	AUY57411.1	44	31	1,634123	384,0729	0,336322552	dprE1 Decaprenylphosphoryl-beta-D-ribose oxidase	108613,4	83246,49	62924,72	70318,14	85597,06	0	2,886969286
AUY57240.1	AUY59322.1	20	19	3,807041	238,5374	0,367243604	dps DNA protection during starvation protein	7589,483	4744,841	5009,002	8175,977	16948,12	40902,65	0,611949062
AUY55483.1	AUY57572.1	21	17	1,505222	140,589	0,078319169	dsbA Thiol:disulfide interchange protein DsbA	14483,94	16199,76	23620,11	24211,8	43272,39	14255,08	3,80704089
AUY56121.1	AUY58210.1	18	12	1,883597	141,8762	0,402382909	dsbG DsbG protein	90729,55	62594,11	57661,22	56769,54	52576,72	2665,437	1,505221797
AUY56581.1	AUY58670.1	40	26	1,39445	380,7486	0,289840825	dtxR Diphtheria toxin repressor	21514,45	19299,8	15102,45	15891,45	15244,64	8963,393	0,530899169
AUY56594.1	AUY58683.1	11	11	2,56636	88,6367	0,170714438	dut Deoxyuridine 5-triphosphate nucleotidohydrolase	9269,511	22888,51	30763,04	30564,07	46835,33	84078,71	0,717128842
AUY56598.1	AUY58687.1	28	24	1,489564	164,1421	0,104915123	dxs 1-deoxy-D-xylulose-5-phosphate synthase	19575,83	50843,05	56968,5	50872,69	34647,24	0	2,566360271
AUY56660.1	AUY58748.1	25	21	1,045917	175,4769	0,386001882	efp Elongation factor P	6400,854	5810,298	4473,758	7606,55	7272,048	2572,432	0,671337567
AUY56826.1	AUY58911.1	23	17	1,630216	127,7282	0,89474577	engA GTP-binding protein EngA	388233,5	398706,5	303020,1	331315,6	332386,8	4896,236	1,045916915
AUY57090.1	AUY59175.1	44	35	1,303704	516,2066	0,350059355	eno Enolase	774,026	2492,699	1457,305	1291,669	915,0364	1416,841	0,613415624
AUY56526.1	AUY58614.1	5	3	3,352916	26,4461	0,659180312	entD 4-phosphopantetheinyl transferase	2223,671	2920,208	1997,602	5372,059	6888,945	11683,78	0,767045548
AUY56282.1	AUY58369.1	14	8	2,510811	92,0853	0,010216716	era GTPase Era	66922,77	57025,48	48650,34	98643,67	139097,5	195621,3	3,352915723
AUY56941.1	AUY59025.1	11	8	1,247665	114,7859	0,015051892	etfA Electron transfer flavo protein subunit alpha	16261,18	19521,62	18356,96	16110,29	19204,63	8077,942	2,510811442
AUY56942.1	AUY59026.1	23	19	1,710155	179,2014	0,354475434	etfB Electron transfer flavo protein subunit beta	29875,74	48218,62	45877,42	33314,2	39177,35	0	0,801497194
AUY55976.1	AUY58067.1	45	34	1,136052	386,3474	0,361528617	eutD Phosphotransacetylase	12927	13874,01	17103,5	21237,16	16827,25	582,1392	0,584742409
AUY56271.1	AUY58358.1	52	32	1,247831	351,4051	0,484331486	fadD15 Long-chain-fatty-acid-CoA ligase	239151,8	191540	176834,1	150866,5	162011,5	173987,7	0,880241164
AUY55862.1	AUY57956.1	49	40	1,896945	579,5062	0,096438046	fadD32 Long-chain-fatty-acid--AMP ligase FadD32	14404,07	32343,48	28966,53	22059,7	17843,79	10,1978	0,801390716
AUY55245.1	AUY57336.1	25	17	2,225441	175,3357	0,346261344	fagD Iron siderophore binding protein	87850,71	75036,95	68180,36	50077,55	53422,21	330,4648	0,527163282
AUY56164.1	AUY58253.1	226	175	2,189459	1913,0063	0,285461017	fas Fatty acid synthase	298266,6	129465,6	109306,3	128363,9	102594,9	14324,77	0,449349153
AUY55950.1	AUY58041.1	28	24	8,212441	319,556	0,245875801	fbaA Fructose-bisphosphate aldolase class 2	247,9781	103,5998	517,6087	523,2157	445,6151	6169,312	0,456733786
AUY55257.1	AUY57348.1	2	2	1,18512	10,874	0,182526856	fecB1 Zinc ABC transporter substrate-binding protein	7177,778	10477,6	11411,75	11485,53	10271,51	2769,702	8,212440755
AUY55791.1	AUY57882.1	24	16	2,415733	149,4403	0,537871387	fecB2 Periplasmic binding protein	11324,2	7879,907	8965,009	5279,038	5790,766	590,8866	0,843796395
AUY56945.1	AUY59029.1	16	11	2,220791	127,5594	0,168803283	fepC1 Ferric enterobactin transport ATP-binding protein FepC	48826,15	60440,71	46263,58	36550,91	33482,36	0,519774	0,413952976
AUY56471.1	AUY58560.1	40	33	1,303204	267,621	0,329805759	ffh Signal recognition particle protein	11134,35	13259,31	4969,577	9068,441	7981,698	21216,14	0,45028993
AUY56758.1	AUY58846.1	27	19	4,160598	167,3443	0,598128845	fhs Formate--tetrahydrofolate ligase	81865,38	13430,1	10454,56	12574,26	12744,65	98,12442	1,303203502
AUY56925.1	AUY59009.1	33	23	1,442309	212,8884	0,276249159	fhuD Iron(3+)-hydroxamate-binding protein	23943,41	26585,88	14803,69	26400,4	18439,57	457,5371	0,240350055
AUY57197.1	AUY59279.1	2	2	1,429905	34,4791	0,394124675	fkbP Peptidyl-prolyl cis-trans isomerase	8888,775	9360,093	8370,719	6958,454	10163,24	1494,635	0,693332883
AUY56679.1	AUY58767.1	22	17	1,067738	165,9883	0,344646675	fmt Methionyl-tRNA formyltransferase	5308,173	8590,434	7681,038	9551,276	10623,54	35,78996	0,699346979
AUY56680.1	AUY58768.1	28	21	2,976225	166,6922	0,464615739	fmu Ribosomal RNA small subunit methyltransferase B	7715,61	4268,797	3005,009	2681,267	2348,598	6,519791	0,936558926
AUY57171.1	AUY59255.1	4	3	2,307506	25,6077	0,264747762	folA Dihydrofolate reductase	2905,192	3363,214	2715,219	1562,232	1360,566	17806,97	0,335996055
AUY56216.1	AUY58304.1	18	13	6,443823	128,2068	0,894373046	folC bifunctional folylpolyglutamate synthase/dihydrofolate synthase FolC	11681,16	8272,881	5348,63	6902,232	7171,636	148972,1	2,30750556
AUY55659.1	AUY57748.1	20	17	2,120086	147,5229	0,443256367	folD Bifunctional protein	1758,594	3770,56	3509,712	3465,221	1741,953	13956	6,443822861
AUY56019.1	AUY58109.1	7	4	1,201526	39,0689	0,550519672	folE GTP cyclohydrolase 1	2859,65	1835,919	1800,163	1779,356	1456,542	4568,893	2,120085908
AUY57023.1	AUY59107.1	3	3	12,94598	22,8955	0,854183804	folP Dihydropteroate synthase	934,9172	311,9337	207,3462	95,63203	16,69607	0	1,201526051
AUY56020.1	AUY58110.1	4	2	1,538455	27,666	0,080481848	folP1 Dihydropteroate synthase	50687,77	58538,35	63091,54	37949,28	35179,82	38877,86	0,077244069
AUY56490.1	AUY58579.1	18	14	1,29762	160,9509	0,003817353	frr Ribosome-recycling factor (RRF)	11398,46	8344,009	6657,763	9467,341	9999,713	14790,42	0,650002786
AUY56564.1	AUY58652.1	18	16	1,042164	138,6624	0,275980326	fruK 1-phosphofructokinase	138597,8	99837,04	84091,76	135019,4	143260	31198,54	1,297620178
AUY56136.1	AUY58226.1	21	15	1,971815	236,5389	0,695263853	ftn Ferritin-like protein	12100,51	12455	12030,21	11549,15	12237,99	48353,12	0,959542169
AUY55787.1	AUY57878.1	20	16	1,452176	129,2469	0,398195998	ftsE Cell division ATP-binding protein FtsE	21471,62	11691,37	13317,93	16251,49	15689,62	66,6676	1,971815121
AUY56018.1	AUY58108.1	70	46	1,930987	533,2944	0,391814825	ftsH Cell division protein	1823,612	2754,37	2880,193	3958,378	3392,056	7051,206	0,688621974
AUY56408.1	AUY58497.1	7	5	1,363416	37,9208	0,078159915	ftsQ Cell division protein FtsQ	9702,786	10648,72	7696,138	5420,736	5262,679	9888,184	1,930987288
AUY55788.1	AUY57879.1	14	11	1,045465	112,8097	0,202756129	ftsX Cell division protein	17984,23	13627,29	12412,86	13119,88	13834,23	15155,73	0,733452003
AUY56468.1	AUY58557.1	40	23	1,777975	272,7887	0,792053682	ftsY cell division protein FtsY	148888,1	41359,95	36483,61	39496,46	35352,61	52673,31	0,956511742
AUY56409.1	AUY58498.1	55	42	2,315764	524,8047	0,468077158	ftsZ Cell division protein	95954,64	57424,42	49210,43	39971,99	45811,71	1699,073	0,562437468
AUY57066.1	AUY59151.1	62	54	1,349738	593,8819	0,245397688	fumC Class II fumarate hydratase	4255,225	9604,327	10699,53	9879,17	10659,58	12609,58	0,431822875
AUY56286.1	AUY58373.1	16	7	1,978312	150,3404	0,284438389	furB Ferric uptake regulatory protein	144948,4	123374,1	120116,6	86316,16	89844,01	20188,61	1,349738211
AUY55549.1	AUY57638.1	84	57	1,465651	797,6687	0,152336057	fusA Elongation factor G	5927,652	15926,95	12510,69	9700,109	11862,88	28804,54	0,505481492
AUY57162.1	AUY59247.1	37	30	2,414409	329,8662	0,485025814	gabD Succinate-semialdehyde dehydrogenase	27747,93	26743,47	22442,62	16366,78	14898,38	599,3813	1,465651417
AUY56580.1	AUY58669.1	29	24	1,677063	246,1017	0,220777429	galE UDP-glucose 4-epimerase	5429,93	3269,092	2791,897	2083,336	2140,435	2628,039	0,414180096
AUY56994.1	AUY59078.1	20	12	1,317577	110,4097	0,087678588	galK Galactokinase	13822,58	11967,87	18875,17	17956,64	40873,03	20,73054	0,596280458
AUY56995.1	AUY59079.1	9	7	1,043225	50,6141	0,501908614	galT Galactose-1-phosphate uridylyltransferase	16997,49	20589,16	28095,96	19824,19	22659,52	26038,04	1,317577283
AUY57134.1	AUY59220.1	39	28	1,155925	308,3145	0,745347955	galU UTP--glucose-1-phosphate uridylyltransferase	498558,5	431897,5	456042,8	537997,4	476634	184839,5	1,043224922
AUY56691.1	AUY58779.1	32	29	8,640454	381,0801	0,514998128	gap Glyceraldehyde-3-phosphate dehydrogenase	3445,647	4204,193	1017,356	4056,48	4062,488	66769,54	0,865107842
AUY56932.1	AUY59016.1	39	28	1,414049	287,3937	0,23655881	gatA Glutamyl-tRNA(Gln) amidotransferase subunit A	20596,29	24114,29	31086,59	54825,84	52319,41	35,6453	8,640453972
AUY56922.1	AUY59007.1	52	37	1,635112	366,944	0,530945739	gatB Glutamyl-tRNA(Gln) amidotransferase subunit B	13759,44	16809,98	12734,87	13600,87	12856,07	27,04788	1,41404878
AUY56567.1	AUY58655.1	19	14	1,540492	161,6471	0,357796924	gatR Galactitol utilization operon repressor	1152,726	4101,699	3519,115	2478,202	3128,589	88,49379	0,611578799
AUY56029.1	AUY58119.1	12	7	4,275859	63,0082	0,432514893	gbsA Betaine aldehyde dehydrogenase	13,86702	3011,693	1152,214	1273,108	1076,534	15513,93	0,649143372
AUY55630.1	AUY57719.1	7	4	2,965299	40,1045	0,338194856	gcp tRNA threonylcarbamoyladenosine biosynthesis protein Gcp	41445,48	6374,806	6647,723	12130,38	7533,838	141849,7	4,27585903
AUY56359.1	AUY58448.1	6	6	1,29238	67,5886	0,577101373	gcvH Glycine cleavage system H protein	67945,48	60813,05	58886,02	97858,04	144526,2	123,7516	2,965299047
AUY56361.1	AUY58450.1	58	40	1,591762	407,2044	0,512735034	gcvP Glycine cleavage system P protein	17627,34	16807,19	19229,45	35590,99	41707,31	8121,994	1,292379855
AUY56360.1	AUY58449.1	56	37	2,109325	397,0523	0,65740736	gcvT Glycine cleavage system T protein	46221,27	29121,03	41433,08	105867,8	129255	11194,39	1,591762025
AUY56459.1	AUY58548.1	53	36	1,067677	405,5083	0,694179975	gdh NADP-specific glutamate dehydrogenase	5866,312	6146,845	6031,74	5489,581	6533,899	7242,641	2,109324657

AUY57304.1	AUY59385.1	16	9	2,27698	100,2422	0,51021062	gidB Glucose-inhibited division protein B	12375,76	20982,06	19603,82	38312,9	74244,83	8034,88	1,06767703
AUY55846.1	AUY57940.1	27	18	1,320851	212,9789	0,502302743	glf UDP-galactopyranose mutase	140894,5	79342,3	76216,55	57024,66	52024,29	115392,2	2,276980026
AUY55852.1	AUY57946.1	65	48	1,936765	468,7527	0,396630646	glfT2 Glycosyltransferase	12903,45	11270,59	10135,76	10956,49	10560,41	44933,13	0,757087419
AUY57016.1	AUY59100.1	18	14	1,039996	138,8179	0,432366021	glgA Glycosyl transferase	7398,549	7795,128	7946,505	11235,57	11014,7	0	1,936765363
AUY56947.1	AUY59031.1	18	15	22,5313	111,2756	0,423712071	glgB Glycogen branching enzyme	340,7213	2088,827	4825,744	8179,947	7576,899	147714,3	0,961542604
AUY57015.1	AUY59099.1	16	15	1,999642	108,9957	0,103638328	glgC Glucose-1-phosphate adenylyltransferase	64672,75	32640,69	38005,96	29798,03	34208,88	3664,903	22,53129586
AUY56946.1	AUY59030.1	17	14	1,940613	116,5564	0,245484843	glgE Glucanase GlgE	23739,28	33917,13	29014,59	20001,88	23974,63	685,1535	0,500089564
AUY56453.1	AUY58542.1	37	27	1,356143	247,5632	0,287891188	glgP Glycogen phosphorylase	20912,5	15011,5	15630,58	39050,74	29180,42	1684,234	0,515301091
AUY56433.1	AUY58522.1	45	38	1,146066	299,9535	0,771794145	glgX2 Glycogen debranching protein	21551,13	22686,69	23749,72	31928,14	27394,39	0	1,356143188
AUY55620.1	AUY57709.1	22	16	1,063305	120,2242	0,406391353	glmM Phosphoglucosamine mutase	17146,97	18458,6	15198,23	15595,04	13947,62	24477,29	0,872550125
AUY56294.1	AUY58383.1	53	37	1,354662	323,5161	0,860858483	glmS glucosamine--fructose-6-phosphate aminotransferase	3747,116	5302,36	6930,999	8293,261	13251,25	103,6317	1,063305248
AUY57098.1	AUY59183.1	23	16	2,060914	180,0569	0,621460362	glmU N-acetyl glucosamine-1-phosphate uridyl transferase	26699,64	23100,53	7299,336	11645,09	14562,81	1498,013	1,354661852
AUY56353.1	AUY58442.1	28	21	1,043436	226,086	0,313545321	glnA Glutamine synthetase 1	2653,355	2682,616	2924,07	3177,267	2573,932	2867,627	0,485221536
AUY56318.1	AUY58407.1	8	7	1,230808	54,8426	0,590556891	glnA2 Glutamine synthetase II	39105,26	25537,35	24980,04	39817,36	32985,23	13,52688	1,043436192
AUY56469.1	AUY58558.1	6	5	22,37335	80,7433	0,411246401	glnB Nitrogen regulatory protein P-II	20,86856	158,8814	287,8408	595,1446	387,5357	9478,894	0,812474639
AUY55979.1	AUY58070.1	5	4	2,049084	29,5049	0,11388904	glnH Glutamine ABC transporter substrate-binding protein GlnH	85296,59	66526,81	85765,96	150934,4	222477	113429,3	22,37335497
AUY55944.1	AUY58035.1	72	56	1,385781	699,2498	0,03201961	glpD Glycerol-3-phosphate dehydrogenase	198082,7	56973,33	90481,75	202397,1	259671	16771,42	2,049084497
AUY55311.1	AUY57401.1	61	51	2,470486	559,7345	0,961201527	glpK Glycerol kinase	1806,478	1078,017	1872,938	6099,755	4565,455	1087,958	1,385780638
AUY55826.1	AUY57918.1	5	3	2,115976	27,1899	0,277605262	glpQ1 Glycerophosphoryl diester phosphodiesterase	10580,62	26373,74	28158,49	33867,18	28883,83	75026,24	2,470485562
AUY55848.1	AUY57942.1	40	26	3,642056	282,0408	0,160109514	glpQ2 Glycerophosphoryl diester phosphodiesterase	2312,032	1909,029	2479,196	789,4355	985,9624	64,29289	2,11597644
CpCAP3W_01807	CpCAPJ4_01801	4	4	1,816836	21,8341	0,11050969	glpR2 Glycerol-3-phosphate regulon repressor	7,051918	134,5363	45,89933	301,4617	39,17234	0	0,274570178
AUY55847.1	AUY57941.1	3	1	1,253295	13,7614	0,763700941	glpT1 Glycerol-3-phosphate transporter	24466,21	22864,86	23121,64	31702,38	24511,62	0	1,816835741
AUY57065.1	AUY59150.1	15	9	1,305719	126,4411	0,39473459	glpX Fructose-1,6-bisphosphatase class 2	65804,67	39165,13	59274,83	96579,11	65845,09	52033,07	0,797897056
AUY57198.1	AUY59280.1	32	21	1,533141	241,5902	0,343468955	gltA Citrate synthase	46086,5	73002,88	78482,42	146792	152540,3	3573,063	1,305718659
AUY56901.1	AUY58986.1	38	28	1,994129	332,2247	0,767196907	gltX1 Glutamyl--tRNA synthetase	12121,29	8278,371	8572,927	7478,202	6504,42	546,3264	1,533140985
AUY57154.1	AUY59239.1	9	6	1,461363	63,1363	0,248332752	gluB Glutamate-binding protein GluB	114506,6	62875,73	79210	96206,48	79262,5	115,244	0,50147218
AUY55419.1	AUY57508.1	29	23	1,202346	267,9774	0,385525872	glxR CRP/FNR family transcriptional regulator	19059,13	28987,72	29845,61	36188,26	23060,85	5534,632	0,684292631
AUY57075.1	AUY59160.1	58	41	1,079717	496,7875	0,507990248	glyA Serine hydroxymethyltransferase	13453,21	20167,81	18151,93	16992,78	15756,69	23150,65	0,831707456
AUY56288.1	AUY58375.1	29	20	2,479071	241,592	0,673615891	glyS Glycyl-tRNA synthetase	11075,17	6947,679	11127,19	4477,455	7174,538	106,4574	1,07971686
AUY56673.1	AUY58761.1	3	2	1,812331	18,2771	0,241749805	gmk Guanylate kinase	60495,72	74699,87	56957,99	53137,21	52842,71	45,72379	0,403376872
AUY56795.1	AUY58883.1	31	27	2,044401	286,7458	0,34209134	gnd 6-phosphogluconate dehydrogenase	297063,7	206947,5	215673,1	177229,9	163338,8	11458,13	0,551775537
AUY55473.1	AUY57562.1	31	24	4,444741	370,9699	0,24668722	gpmA Phosphoglyceromutase	1822,997	1092,584	4889,868	858,8312	896,3294	0,948341	0,489140735
AUY55497.1	AUY57586.1	5	3	5,605872	28,0984	0,240911639	gpmB1 Probable phosphoglycerate mutase	482,9081	723,8037	1199,516	1822,343	1071,533	10595,13	0,224985011
AUY55477.1	AUY57566.1	10	5	2,010046	64,4175	0,154220414	gppA1 Ppx/GppA phosphatase family protein	1448,902	1451,654	2049,627	1087,14	1375,581	0	5,605872002
AUY57086.1	AUY59171.1	8	3	1,026932	39,8527	0,329827707	gppA2 Ppx/GppA phosphatase family protein	52187,6	27695,73	32141,91	43921,41	58705,63	12415,25	0,497501055
AUY56891.1	AUY58976.1	11	10	1,574859	56,5714	0,820978479	gpsA Glycerol-3-phosphate dehydrogenase	35491,06	25053,04	27237,15	21944,26	24358,39	9436,454	1,026931916
AUY57082.1	AUY59167.1	22	20	1,341663	201,3142	0,17545477	greA Transcription elongation factor GreA	130478,8	423895,3	357846,6	359194,6	291319,8	29403,4	0,634977388
AUY55632.1	AUY57721.1	65	53	1,257977	691,1083	0,520536659	groEL Chaperonin	127003,4	241366,3	251565,4	196656,5	179290,4	403917,1	0,745343494
AUY56003.1	AUY58094.1	61	48	1,625839	702,5506	0,578814163	groEL1 Chaperonin GroEL	64228,09	62778,24	94358,72	86806,3	48096,98	1251,034	1,25797693
AUY55915.1	AUY58009.1	11	8	1,879759	119,0208	0,343763946	grpE Molecular chaperone GrpE	17360,26	16398,9	13204,83	18511,98	20115,04	49654	0,615066872
AUY55638.1	AUY57727.1	47	29	1,166645	351,7347	0,179485492	guaA GMP synthase	21406,56	26248,66	23719,92	32371,4	27441,57	23456,51	1,879759416
AUY55635.1	AUY57724.1	40	29	1,344183	266,5922	0,247383645	guaB Inosine-5'-monophosphate dehydrogenase	58034,87	49196,65	39495,15	33098,02	45062,41	30996,35	1,166645281
AUY55636.1	AUY57725.1	31	22	1,2315	251,7968	0,137541672	guaB2 Inosine-5'-monophosphate dehydrogenase	20959,39	20693,43	18348,81	22774,81	24816,58	1131,007	0,743946384
AUY55225.1	AUY57316.1	71	48	2,085316	495,8814	0,454693748	gyrA DNA gyrase subunit A	31404,27	33121	39519,02	26747,95	23145,15	0,6821	0,812018016
AUY55221.1	AUY57312.1	58	37	5,624701	456,7874	0,334584942	gyrB DNA gyrase subunit B	621,2068	1198,788	807,8919	367,1288	234,6062	14179,34	0,479543634
AUY55487.1	AUY57576.1	12	5	1,232952	93,6305	0,866091943	hemA Glutamyl-tRNA reductase	3034,31	4428,84	3413,183	3916,383	4904,992	0	5,624701128
AUY55490.1	AUY57579.1	6	4	3,905788	38,2081	0,403981568	hemB Delta-aminolevulinic acid dehydratase	12883,45	8538,153	7440,934	6896,258	7521,237	98313,47	0,811061463
AUY55488.1	AUY57577.1	25	19	1,212904	157,4802	0,529608295	hemC Porphobilinogen deaminase	8864,655	4180,426	6163,267	6143,734	9440,264	252,6588	3,905788391
AUY55489.1	AUY57578.1	8	3	2,359638	54,1379	0,475032269	hemD Uroporphyrinogen-III synthase	8919,396	6286,845	6514,507	4018,252	3407,419	1779,447	0,824467403
AUY55493.1	AUY57582.1	6	4	1,982052	47,2567	0,029543383	hemE Uroporphyrinogen decarboxylase	26808,58	24077,98	14549,23	21876,82	22854,4	84965,92	0,423793819
AUY55496.1	AUY57585.1	60	41	2,349995	496,3813	0,356760501	hemL Glutamate-1-semialdehyde 2,1-aminomutase	8742,223	2718,903	4493,773	2568,335	1716,207	2504,792	1,982052371
AUY55494.1	AUY57583.1	15	9	2,618564	106,9224	0,105208267	hemY Protoporphyrinogen oxidase	1335,451	2291,536	3965,537	4825,874	6142,998	8912,637	0,425532836
AUY56558.1	AUY58646.1	8	7	1,677552	37,9464	0,046772196	hflX GTPase HflX	25724,51	15461,91	17006,88	35121,46	44113,42	18387,39	2,618563801
AUY56444.1	AUY58533.1	27	23	1,120536	213,7645	0,191361117	hisA Phosphoribosylformimino-5-aminoimidazole carboxamide	3626,608	4321,142	4513,017	5552,355	5019,242	548,7714	1,67755184
AUY56439.1	AUY58528.1	13	5	1,279911	86,1533	0,537650126	hisC Histidinol-phosphate aminotransferase	27146,19	14489,89	15869,66	32693,43	40793,31	115,4971	0,892430424
AUY56438.1	AUY58527.1	14	10	2,581592	94,7186	0,557330244	hisD Histidinol dehydrogenase	9006,432	5911,442	4974,536	5628,479	4201,876	41523,72	1,279911093
AUY56446.1	AUY58535.1	12	9	1,079675	85,9228	0,587255226	hisF Imidazole glycerol phosphate synthase subunit HisF	15463,42	13649,28	11110,16	25981,33	8272,989	3000,273	2,581591593
AUY56754.1	AUY58843.1	22	16	1,112994	159,8062	0,532670093	hisG ATP phosphoribosyl transferase	10505,43	11228,73	18131,36	17745,41	17538,26	534,6014	0,926204289
AUY56443.1	AUY58532.1	9	9	12,78886	65,7289	0,509942182	hisH Imidazole glycerol phosphate synthase subunit	5413,197	1932,814	2033,228	368,2399	365,1516	0	0,898477297
AUY55782.1	AUY57873.1	3	1	1,162147	16,3469	0,13058878	hisN Histidinol-phosphatase	26251,91	33275,83	31503,98	38682,53	63840,04	3269,682	0,078193063
AUY56635.1	AUY58723.1	51	33	3,602971	335,7288	0,680236623	hisS histidyl-tRNA synthetase	14306,53	19770,88	18319,9	48027,45	59294,37	81464,16	1,162147068
AUY55925.1	AUY58018.1	60	48	1,621065	502,9537	0,002235811	hlyD Secretion protein HlyD	7621,551	12046,38	16924,66	18003,18	37247,26	4068,535	3,602970886
AUY55664.1	AUY57753.1	7	5	1,34823	41,0882	0,799537094	hmuT Hemin-binding periplasmic protein	29728,42	12055,21	18960,75	15218,48	29799,7	36,72856	1,621065188
AUY55666.1	AUY57755.1	9	2	4498,028	45,4104	0,403244494	hmuV Hemin import ATP-binding protein HmuV	0,98862	0	1,476125	0,195601	0,587116	11085,71	0,741713348
AUY56017.1	AUY58107.1	11	9	1,122633	92,867	0,420011878	hpt Hypoxanthine-guanine phosphoribosyltransferase	87374,69	59118,8	71945,07	116725,3	77534,5	317,1861	4498,028126
AUY55917.1	AUY58011.1	7	3	1,400468	45,4867	0,44342416	hspR Heat shock protein HspR	17496,79	21974,21	19479,59	22609,65	24647,51	35301,25	0,890762884
AUY55668.1	AUY57757.1	88	64	1,63928	722,5245	0,100747663	icd Isocitrate dehydrogenase	344,3386	374,7338	376,0473	141,9879	239,2423	1413,977	1,400467929
AUY55356.1	AUY57446.1	2	2	1,092281	9,3434	0,996350301	idnD Zinc-binding alcohol dehydrogenase	7100,754	2815,326	5470,161	4188,309	3965,5	8652,297	1,639279526
AUY56390.1	AUY58479.1	17	14	6,413569	114,0339	0,81911086	idsA Geranylgeranyl pyrophosphate synthase	3700,258	4175,141	3573,484	3480,688	3563,606	66383,91	1,092281489
AUY56415.1	AUY58504.1	38	28	18,91021	236,196	0,410008179	ileS isoleucyl-tRNA synthetase	178,3962	354,65	251,3982	197,1603	116,6983	14520,15	6,413568744
AUY56425.1	AUY58514.1	6	5	2,73504	28,5519	0,546087089	ilvA Threonine dehydratase	46447,77	40295,24	39676,99	19995,98	22647,13	3579,255	18,91021211
AUY56061.1	AUY58152.1	47	35	5,299623	386,4486	0,09975569	ilvB1 Acetolactate synthase large subunit IlvB1	3077,389	5549,644	3406,237	5392,485	8066,73	50312,57	0,365625395
AUY56913.1	AUY58998.1	24	17	1,745528	147,9297	0,1641712	ilvC Ketol-acid reductoisomerase	31011,62	38335,92	31412,99	29983,91	29009,54	116886,9	5,299622707
AUY56365.1	AUY58454.1	23	17	1,772535	194,3655	0,511827233	ilvE Branched-chain amino acid aminotransferase	8566,341	9622,538	9649,78	7919,392	6913,541	872,6305	1,745527915
AUY55783.1	AUY57874.1	8	6	2,420255	49,7137	0,259853721	impA1 Inositol-1-monophosphatase ImpA	1562,236	1469,452	1068,348	1014,538	678,6956	0,817579	0,564163764
AUY56445.1	AUY58534.1	4	2	6,566384	25,5438	0,291136041	impA2 Inositol monophosphate phosphatase	1488,105	1895,345	1860,52	3533,613	3317,69	27582,62	0,413179641
AUY55603.1	AUY57692.1	10	6	1,201713	81,9976	0,121234085	infA Translation initiation factor IF-1	39222,31	39246,42	38652,39	45520,19	22771,45	72454,35	6,566384153
AUY56521.1	AUY58609.1	47	35	5,354588	397,98	0,828764792	infB Translation initiation factor IF-2	172,3201	2538,861	3477,706	2562,796	4419,754	26156,4	1,201713132
AUY56863.1	AUY58949.1	5	3	1,173829	35,6751	0,211834448	infC Translation initiation factor IF-3	6303,596	4631,673	5004,19	5696,92	5846,685	7166,6	5,354588189
AUY55519.1	AUY57608.1	9	7	1,321799	49,9442	0,236551777	ispB Polyprenyl synthetase	259,9375	737,3312	661,2829	295,4108	248,2551	711,1023	1,173829304
AUY56049.1	AUY58140.1	3	2	15,51982	17,0478	0,558431073	ispD 2-C-methyl-D-erythritol 4-phosphate cytidylyltransferase	498,3457	1088,304	946,5346	1112,277	1237,116	36965,17	0,756544501
AUY57119.1	AUY59205.1	4	2	4,541586	32,0552	0,262406572	ispE 4-diphosphocytidyl-2-C-methyl-D-erythritol kinase	10916,43	18969,35	17426,06	25409,78	37741,68	151719,3	15,51981812
AUY56498.1	AUY58586.1	41	28	1,164708	290,56	0,096045005	ispG 4-hydroxy-3-methylbut-2-en-1-yl diphosphate synthase	3433,114	8426,366	8705,718	6875,72	10726,35	54,88949	4,541586111
AUY56036.1	AUY58126.1	5	5	1,888534	39,4676	0,467415387	iunH2 Inosine-uridine preferring nucleoside hydrolase	144211,5	117039,8	97046,03	78243,81	83264,37	28214,29	0,858584419
AUY55389.1	AUY57479.1	66	51	2,138201	699,8824	0,119367147	katA catalase	18613,27	15989,07	12077,65	12629,8	7673,575	1528,056	0,529511159
AUY57120.1	AUY59206.1	12	6	1,866286	99,5381	0,177215208	ksgA Dimethyladenosine transferase	58601,56	60780,99	57300,34	46862,81	46070,25	1737,815	0,467682854
AUY55471.1	AUY57560.1	58	44	1,602496	484,5811	0,290843777	lcfA1 long-chain-fatty-acid--CoA ligase	1817,018	4388,479	5231,28	3732,859	5314,695	9279,833	0,535823693
AUY56968.1	AUY59052.1	3	2	1,409691	14,8058	0,305057108	lcfA2 Long-chain-fatty-acid--CoA ligase	74303,89	76177,56	50242,03	75539,32	61436,35	5412,602	1,602495891
AUY55517.1	AUY57606.1	25	21	1,750346	249,0374	0,398663461	ldh1 L-lactate dehydrogenase	95541,11	83001,95	75407,19	78113,37	66972,35	0	0,709375291

AUY55822.1	AUY57914.1	32	26	1,074446	294,2612	0,357772987	ldh2 L-lactate dehydrogenase	6791,353	11840,49	8205,792	10635,53	9611,226	4731,358	0,571315513
AUY56241.1	AUY58329.1	47	34	5,369455	277,1353	0,749383218	lepA GTP-binding protein LepA	1399,615	1281,929	624,6747	1796,575	1509,368	14446,65	0,930712087
AUY56480.1	AUY58569.1	3	3	1,174408	14,3041	0,198426016	lepB Signal peptidase I	4264,728	3270,559	4053,269	6193,101	7416,588	0	5,369455061
AUY55373.1	AUY57463.1	16	9	1,906246	84,2748	0,458099478	leuA 2-isopropylmalate synthase	13170,87	19136,43	20961,22	18379,46	10499,88	72663,56	1,174407741
AUY57246.1	AUY59328.1	58	42	1,327143	426,1754	0,611827818	leuS Leucyl-tRNA synthetase	4243,992	3906,268	4296,355	5001,626	4369,681	7,193889	1,90624616
AUY56740.1	AUY58829.1	6	4	2,070766	55,0459	0,398619716	levD Fructose-specific phosphotransferase enzyme IIA component	15001,52	13935,75	12058,63	9922,335	9875,124	0	0,75349809
AUY56935.1	AUY59019.1	41	28	1,059524	267,3018	0,334503905	ligA NAD-dependent DNA ligase LigA	2730,933	5579,198	7950,103	6535,393	10692,71	0	0,482913112
AUY56356.1	AUY58445.1	27	14	2,792811	171,3488	0,451439741	lipA Lipoyl synthase	6403,987	7459,381	11351,24	19456,11	11302,16	39661,37	1,059523554
AUY56011.1	AUY58101.1	11	8	2,853775	82,9538	0,082513647	lipY Secretory lipase	67170,82	49203,72	34282,47	23379,72	24910,67	4501,799	2,792811415
AUY55455.1	AUY57544.1	56	41	1,253391	469,5005	0,101585454	lpd Dihydrolipoamide dehydrogenase	18970,86	16637,79	10718,44	19445,42	24150,68	14469,86	0,350413072
AUY55683.1	AUY57772.1	48	39	1,002375	330,8252	0,364976206	lpdA Flavoprotein disulfide reductase	8376,864	11858,15	12630,23	11298,33	12137,1	9507,865	1,253390923
AUY56900.1	AUY58985.1	14	8	58,17505	83,1872	0,934605773	lpqC Poly(3-hydroxybutyrate) depolymerase	41436,1	1012,62	732,402	466,7443	255,6644	19,85318	1,002374935
AUY56047.1	AUY58138.1	9	7	1,394751	58,7874	0,122529832	lpqE Lipoprotein LpqE	2950,201	6522,305	5952,058	7212,362	8554,298	5746,762	0,017189499
AUY57036.1	AUY59120.1	16	13	1,190368	89,7616	0,243794704	lpqW Monoacyl phosphatidylinositol tetramannoside-binding protein LpqW	4998,496	21097,24	32918,5	11014,45	11345,09	27216,92	1,394750801
AUY56975.1	AUY59059.1	24	19	1,392012	212,0824	0,992019259	lutA Lactate utilization protein A	31348,05	45577,8	42117,07	26931,83	18000,95	40585,8	0,840076294
AUY56976.1	AUY59060.1	81	61	1,238925	622,5133	0,226376213	lutB Lactate utilization protein B	16753,62	19806,67	18999,69	24859,69	19762,38	223,2409	0,718384377
AUY56972.1	AUY59056.1	13	7	2,130219	78,0868	0,428624992	lysA1 Diaminopimelate decarboxylase	32350,78	37872,82	33013,63	23368,01	21681,19	3414,016	0,80715125
AUY55384.1	AUY57474.1	36	29	1,392506	302,8059	0,171211529	lysC Aspartate kinase	36327,35	27430,97	25493,89	30139,08	33955,6	0	0,469435374
AUY56033.1	AUY58123.1	45	33	2,041494	325,7841	0,384056136	lysS Lysyl-tRNA synthetase	22812,18	29000,73	23164,31	18605,27	18046,96	74,42515	0,718129822
AUY55888.1	AUY57982.1	89	55	1,163323	679,1546	0,308870573	lysS1 Lysyl-tRNA synthetase	5939,454	11856,54	11128,71	10279,73	8123,968	15245,08	0,489837458
AUY55710.1	AUY57801.1	24	14	5,343056	173,8574	0,60319221	lytR1 Transcriptional regulator	48342,25	50426,24	55207,95	86107,61	109903,6	626693,4	1,163323134
AUY55830.1	AUY57922.1	6	4	1,029788	39,8511	0,114105454	lytR2 LytR family transcriptional regulator	10747,58	6036,119	6213,537	10101,18	13311,08	270,012	5,343055713
AUY55692.1	AUY57783.1	8	6	4,924344	50,8937	0,561751966	maf Maf-like protein	6859,021	9230,255	5884,009	4372,274	3384,817	100446,9	1,029787765
AUY55591.1	AUY57679.1	30	23	3,456496	236,0223	0,695957679	malE1 Maltotriose-binding protein	2344,86	4074,939	5312,454	1718,563	1070,138	37763,78	4,92434385
AUY55708.1	AUY57799.1	18	16	1,106479	111,1317	0,93155028	malE2 Maltose/maltodextrin transport system substrate-binding protein	51470,48	45203,7	46514,11	46665,76	41777,98	69991,02	3,456495928
AUY55597.1	AUY57685.1	34	25	1,084586	243,2556	0,657729115	malK Glycerol-3-phosphate-transporting ATPase	8008,318	7636,419	11754,66	13008,57	11995,54	258,4337	1,106478531
AUY55261.1	AUY57352.1	15	7	6,615477	96,6551	0,501169287	malL Oligo-1,6-glucosidase 1	2835,316	5367,385	517,2737	6696,551	6102,851	44887,39	0,922010935
AUY55719.1	AUY57809.1	15	11	1,72651	103,7682	0,129564775	manA Mannose-6-phosphate isomerase	5254,678	5873,746	6014,473	4844,752	4796,998	287,4714	6,615477182
AUY55681.1	AUY57770.1	17	12	1,359452	124,2277	0,304120286	manB1 Phosphoglucomutase/phosphomannomutase	14341,92	23381,75	14332,73	18608,32	19681,22	2,658902	0,579203252
AUY55717.1	AUY57807.1	38	22	5,680996	283,0732	0,393257556	manB2 Phosphomannomutase ManB	6207,775	4595,745	2746,506	6070,779	7473,52	63433,35	0,735590427
AUY55712.1	AUY57803.1	43	31	1,360825	310,0806	0,201553927	manC Mannose-1-phosphate guanylyltransferase	14802,89	35020,25	34363,56	37813,81	24050,62	0	5,680996347
AUY56477.1	AUY58566.1	4	1	2,151121	26,1337	0,392612681	manP PTS system fructose-specific transporter subunit IIABC	26962,59	22647,56	17438,12	18269,25	12784,87	114,8702	0,734848159
AUY56500.1	AUY58588.1	10	8	1,030082	100,2582	0,290364462	mapB Methionine aminopeptidase	7625,5	3983,809	5081,034	4985,885	4628,314	6588,725	0,464873951
AUY56010.1	AUY58100.1	7	3	10,29478	55,4051	0,982680034	marR2 Multiple antibiotic resistance protein marR	8125,029	4971,099	11205,03	3912,4	6462,966	239799,7	0,970796317
AUY57234.1	AUY59316.1	28	20	1,01632	245,3169	0,547373256	marR3 Multiple antibiotic resistance protein marR	27857,3	43552,05	15785,09	30000,66	21243,07	34550,58	10,29477676
AUY56176.1	AUY58265.1	14	11	1,071559	129,2445	0,893842527	mas Mycocerosic acid synthase	49016,86	31267,58	49769,43	39938,96	81281,3	148,5295	0,983942531
AUY57080.1	AUY59165.1	17	14	1,618211	118,7531	0,446117817	mca Mycothiol conjugate amidase Mca	1621,447	1129,993	1956,883	2609,574	1653,138	3356,349	0,933219416
AUY55800.1	AUY57892.1	3	2	1,350984	13,568	0,151033228	mcbR TetR family transcriptional regulator	143677,4	165211,3	88578,31	150395,7	143774,6	35,12097	1,618211243
AUY56214.1	AUY58302.1	33	24	2,722153	340,2828	0,396715422	mdh Malate dehydrogenase	8508,568	8727,303	6489,798	4581,351	4126,774	7,649093	0,740200965
AUY55510.1	AUY57599.1	14	9	1,766163	85,7642	0,270505254	menB Naphthoate synthase	13129,78	12291,9	11504,25	9220,357	11687,07	0	0,367356311
AUY56801.1	AUY58889.1	16	13	1,155197	123,7456	0,352691324	merR3 MerR family transcriptional regulator	14265,9	9828,732	9905,765	26856,68	11935,76	484,7211	0,566199079
AUY57123.1	AUY59209.1	36	26	1,017117	254,194	0,585234138	metG methionyl-tRNA synthetase	9122,035	10092,26	10400,91	11904,98	18206,72	10,44663	1,155196805
AUY56676.1	AUY58764.1	20	14	3,047786	142,5663	0,451819918	metK S-adenosylmethionine synthase	24367,66	18546,86	20538,38	12491,92	8327,415	0	1,017117463
AUY55646.1	AUY57735.1	24	15	1,436194	173,6021	0,290340901	metN Methionine import ATP-binding protein MetN	10372,38	2214,895	3766,206	4182,729	7178,741	25,21429	0,328106988
AUY55647.1	AUY57736.1	3	2	1,622845	15,1534	0,442839295	metQ D-methionine-binding lipoprotein	22438,61	26379	8676,17	18904,29	15467,52	58931,66	0,696284888
AUY55649.1	AUY57738.1	27	14	83,18596	212,676	0,497931728	metQ1 D-methionine-binding lipoprotein metQ	146,3675	25,72208	37,51888	42,96595	114,6061	17278,91	1,622844505
AUY57094.1	AUY59179.1	8	7	1,474076	37,6849	0,331142911	mfd Transcription-repair coupling factor	6915,232	7159,843	15975,56	16554,05	18505,16	9237,718	83,18596232
AUY57008.1	AUY59092.1	25	14	1,551608	184,5623	0,289328177	mgtE2 Magnesium transporter mgtE	15011,34	13859,45	12155,76	11995,64	12319,6	2126,072	1,474076222
AUY56903.1	AUY58988.1	12	9	2,192586	99,7574	0,300063608	mhpD 2-hydroxyhepta-2,4-diene-1,7-dioate isomerase	165288,7	23842,56	23381,3	24079,93	23180,22	49663,12	0,644492801
AUY56672.1	AUY58760.1	16	11	6,700874	150,0441	0,595125132	mihF Integration host factor MihF	17886,77	25594,43	8591,524	6305,803	1465,232	0	0,456082495
AUY55869.1	AUY57963.1	10	7	1,927037	61,0273	0,197987253	mmpL Transmembrane transport protein MmpL	2454,121	3258,214	3087,856	4301,134	6535,013	6122,142	0,149234266
AUY55495.1	AUY57584.1	16	13	1,33036	114,1031	0,014374692	mmsB2 3-hydroxyisobutyrate dehydrogenase	2160,994	2500,523	1631,938	4380,836	3991,721	0	1,927036601
AUY56938.1	AUY59022.1	2	1	2,526268	10,6493	0,486864071	mnmA tRNA (5-methyl aminomethyl-2-thiouridylate)-methyltransferase	4149,909	12114,13	8930,741	5997,252	3975,868	0	1,330359511
AUY55531.1	AUY57620.1	4	2	1,6569	21,7412	0,317948644	mntB2 Manganese transport system ATP-binding protein MntB	3201,68	7734,057	5891,583	4716,903	3070,76	2368,245	0,395840767
AUY55654.1	AUY57743.1	7	4	2,097803	48,0997	0,216540146	mntB3 manganese ABC transporter ATP-binding protein	5043,157	6071,602	4029,751	3002,373	2821,006	1395,846	0,603536863
AUY55657.1	AUY57746.1	8	6	1,296095	70,599	0,045601627	mntR DtxR family transcriptional regulator	17969,83	9307,089	9594,479	6797,94	6210,408	34780,47	0,476689288
AUY57139.1	AUY59224.1	14	11	2,088148	87,3659	0,963004839	moaB molybdenum cofactor biosynthesis protein MoaB	6438,216	3995,798	29952,29	7701,557	6787,908	4851,26	1,296094712
AUY57133.1	AUY59219.1	15	10	2,023921	97,4695	0,584237611	moeA Molybdopterin molybdenum transferase	24470,76	41367,83	28682,89	21668,1	25018,1	15,96186	0,478893185
AUY56506.1	AUY58594.1	64	49	1,222814	505,5984	0,32848999	mqo Malate:quinone oxidoreductase	7351,257	4380,055	3372,288	2629,788	5270,255	10568,85	0,494090467
AUY56397.1	AUY58486.1	20	13	1,133256	112,7419	0,839643895	mraW S-adenosyl-methyltransferase MraW	1368,19	3705,736	4103,046	3799,761	4297,587	0,530269	1,222813865
AUY56396.1	AUY58485.1	7	4	1,446085	50,8555	0,43955484	mraZ cell division protein MraZ	6114,332	9301,501	7484,473	5191,222	4228,632	6416,215	0,88241295
AUY57010.1	AUY59094.1	16	14	1,435164	109,3075	0,097022852	mrp Protein	13560,37	27594,78	23899,6	14719,98	14694,91	15914,25	0,691522212
AUY57137.1	AUY59223.1	10	9	2,266282	70,7644	0,218911708	mscL Large-conductance mechanosensitive channel	3043,584	2716,921	2183,833	1605,238	1474,462	425,7504	0,696784494
AUY55472.1	AUY57561.1	7	4	1,819366	40,2305	0,093999461	mshA D-inositol-3-phosphate glycosyltransferase	4927,798	6379,107	5117,943	4042,832	4404,721	580,2424	0,441251435
AUY57035.1	AUY59119.1	3	3	1,009888	19,8305	0,241810019	mshB MshB deacetylase	21189,64	13322,05	9880,257	15879,25	14404,24	13673,82	0,54964187
AUY56749.1	AUY58838.1	12	9	1,715156	69,6385	0,87417437	mshC L-cysteine:1D-myo-inositol 2-amino-2-deoxy-alpha-D-glucopyranoside ligase	4566,954	3219,554	3798,819	4342,573	2412,107	0	0,990209077
AUY56093.1	AUY58184.1	6	4	3,6538	34,2551	0,351135346	mshD1 Mycothiol biosynthesis acetyltransferase	11910,7	4601,592	3732,779	3002,625	2089,105	449,0972	0,583037452
AUY56931.1	AUY59015.1	6	6	1,236648	37,1033	0,104700626	mshD2 mycothiol acetyltransferase	1412,893	3250,017	2393,325	3008,51	2697,428	0	0,273687672
AUY55821.1	AUY57912.1	3	1	1,117895	26,6692	0,413961908	msrA Peptide methionine sulfoxide reductase	24561,22	5043,717	3961,478	3578,893	3095,324	23352,24	0,808637777
AUY55725.1	AUY57816.1	15	12	1,091164	116,4225	0,817571813	mtrA DNA-binding response regulator	28023,48	24773	28473,43	34573,12	44158,49	9947,209	0,894538634
AUY56113.1	AUY58202.1	39	25	2,730062	323,1909	0,858890959	murA UDP-N-acetylglucosamine 1-carboxyvinyltransferase	7352,826	661,9226	703,606	842,5642	1455,52	21503,57	1,091164347
AUY55470.1	AUY57559.1	9	6	1,62252	51,4289	0,622361273	murB UDP-N-acetylenolpyruvoylglucosamine reductase	1406,483	4066,414	3926,043	2939,533	2805,345	47,92373	2,730062458
AUY56407.1	AUY58496.1	11	4	1,449661	67,8285	0,393070234	murC UDP-N-acetyl muramate--L-alanine ligase	2456,058	2723,475	2136,658	2437,088	2093,561	6075,349	0,61632506
AUY55371.1	AUY57461.1	13	11	1,714196	73,34	0,490171736	murD1 UDP-N-acetylmuramoylalanine--D-glutamate ligase	994,3483	1619,114	921,2036	1130,42	930,2867	1,288942	1,449661229
AUY56403.1	AUY58492.1	5	4	1,441831	35,4939	0,35507008	murD2 UDP-N-acetylmuramoyl-L-alanyl-D-glutamate synthetase	19466,28	6882,54	6583,719	7088,172	7130,393	33264,59	0,583363777
AUY56400.1	AUY58489.1	24	15	1,025649	158,7977	0,747979819	murE UDP-N-acetylmuramoylalanyl-D-glutamate--2, 6-diaminopimelate ligase	3267,078	5673,507	2648,646	2462,204	2793,915	6043,293	1,441831207
AUY56405.1	AUY58494.1	6	5	1,170247	37,808	0,885407594	murG N-acetyl glucosaminyl transferase	45246,07	47328,42	32595	47952,88	47533,56	11473,44	0,974992321
AUY56731.1	AUY58820.1	37	31	1,63244	326,749	0,540482598	mutA Methylmalonyl-CoA mutase small subunit	2240,642	1338,228	1311,161	875,5746	1255,675	5851,435	0,854520387
AUY56892.1	AUY58977.1	11	9	3,108966	68,2259	0,800613481	mutT2 7,8-dihydro-8-oxoguanine-triphosphatase	1623,125	611,5781	611,4409	1352,343	1144,505	6351,717	1,632440489
AUY56042.1	AUY58132.1	2	2	2,32457	10,122	0,217733152	mutY A/G-specific DNA glycosylase	16362,16	29054,2	26399,44	16883,49	14010,74	0	3,108965765
AUY56131.1	AUY58221.1	9	5	1,374773	71,6231	0,32610107	nadE NH(3)-dependent NAD(+) synthetase	4705,08	5591,891	5338,065	5887,838	5471,298	13,67874	0,430187174
AUY57257.1	AUY59339.1	7	6	2,607022	36,7719	0,393270528	nagA1 N-acetylglucosamine-6-phosphate deacetylase	86305,15	26854,9	72817,91	125642	262907,3	96299,29	0,727392957
AUY57256.1	AUY59338.1	11	9	2,093578	73,8379	0,10682017	nagB Glucosamine-6-phosphate isomerase	1806,683	1830,505	1587,119	2116,229	1533,134	7288,129	2,60702199
AUY57258.1	AUY59340.1	8	7	1,015676	43,2562	0,352485101	nanE N-acylglucosamine-6-phosphate 2-epimerase	48608,49	29884,58	44272,87	70578,41	50289,41	3,341807	2,093577501
AUY55601.1	AUY57690.1	68	47	1,231541	546,267	0,430089302	nanH neuraminidase	4724,878	3248,745	4101,698	2610,052	7189,965	5,030878	0,984565953
AUY57266.1	AUY59347.1	14	9	2,720135	95,1851	0,394559892	nanL Dihydrodipicolinate synthase	99805,44	45900,63	37381,69	31697,54	35595,39	15,40984	0,81199072
AUY56791.1	AUY58879.1	45	38	3,040705	368,4197	0,300530056	ndh NADH dehydrogenase	9950,224	10744,67	5122,947	6399,087	3745,818	68359,54	0,36762884
AUY56219.1	AUY58307.1	16	15	1,34745	136,3513	0,712414503	ndk Nucleoside diphosphate kinase	10785,58	9485,608	12665,79	9009,499	15365,35	69,08754	3,040705399
AUY55525.1	AUY57614.1	13	12	1,401502	89,4426	0,395675069	nemA N-ethylmaleimide reductase	2016,56	1655,531	2125,868	2358,636	1122,653	4644,562	0,742142674

AUY56711.1	AUY58799.1	5	2	1,008728	36,3726	0,683062285	nifU NifU family protein	2375,548	4194,407	3029,651	3217,46	3295,877	3170,051	1,401502045
AUY55343.1	AUY57433.1	5	4	1,123306	26,3478	0,83865245	norB Nitric-oxide reductase, cytochrome b-containing subunit I	7708,31	10757,56	10957,39	11695,82	11106,05	3391,584	1,008727614
AUY55286.1	AUY57376.1	19	15	1,641225	150,7242	0,596496992	noxC NADH dehydrogenase	888,1687	4338,529	3680,703	5142,535	2240,817	7235,7	0,890229325
AUY55545.1	AUY57634.1	9	7	6,57962	64,7234	0,38937624	nrdD Anaerobic ribonucleoside triphosphate reductase	7388,59	3052,321	5087,618	3332,23	7406,762	91432,83	1,641225256
AUY56135.1	AUY58225.1	49	34	1,925159	354,759	0,389702862	nrdE Ribonucleoside-diphosphate reductase subunit alpha	16138,25	22090,46	28187,48	15557,04	17755,02	1187,013	6,579619944
AUY56137.1	AUY58227.1	25	19	2,535324	196,4777	0,27152381	nrdF2 ribonucleoside-diphosphate reductase subunit beta	1544,023	5063,79	4619,865	3208,18	3364,517	21893,1	0,519437678
AUY56570.1	AUY58659.1	12	11	3,699638	81,9063	0,444336212	nrdR Transcriptional repressor NrdR	2795,387	4440,607	4764,281	6151,955	3905,183	34339,53	2,535323875
AUY55962.1	AUY58053.1	23	10	10,54976	126,079	0,267675721	nrfA Nitrite reductase periplasmic cytochrome c552	25748,74	1885,715	3069,967	1074,728	931,3361	904,3746	3,699637771
AUY55423.1	AUY57512.1	2	2	1,104345	12,6901	0,10189206	nudL Nudix hydrolase	9078,312	11309,41	11549,75	10194,38	9677,316	9048,139	0,094788907
AUY56519.1	AUY58607.1	34	22	1,28684	231,8949	0,324684945	nusA Transcription elongation protein	11170,08	7596,143	8068,769	10674,06	5532,529	18325,76	0,905514381
AUY56661.1	AUY58749.1	8	6	1,303643	51,2939	0,698380644	nusB N utilization substance protein B	26450,23	13916,95	11847,35	21350,48	46718,62	0	1,286840226
AUY55521.1	AUY57610.1	26	19	1,094963	201,6849	0,459403378	nusG Transcription anti-termination protein NusG	6978,51	16255,6	17023,11	15821,08	11984,74	16274,33	1,30364262
AUY56224.1	AUY58312.1	22	19	2,162793	143,5215	0,636374695	obgE GTPase ObgE	60489,5	17412,22	25323,4	55656,22	167565,3	33,09222	1,094962645
AUY57006.1	AUY59090.1	119	94	2,316687	867,8728	0,611752641	odhA 2-oxoglutarate dehydrogenase E1 component	153701,6	56462,6	50834,24	47037,31	42690,87	22932,02	2,162793233
AUY56804.1	AUY58892.1	19	13	11,39608	175,0849	0,146132365	odhI Oxoglutarate dehydrogenase inhibitor	710,0645	2450,5	2366,752	5005,853	3245,788	54738,11	0,431650864
AUY55570.1	AUY57659.1	34	26	1,930055	210,5074	0,137922972	oppA1 oligopeptide-binding protein OppA	11592,02	34592,73	41283,97	74928,62	70782,44	23108,4	11,39608173
AUY57179.1	AUY59262.1	43	28	1,602903	272,8504	0,293102153	oppA2 Oligopeptide-binding protein OppA	70568,92	23458,3	26259,14	20714,97	53553,25	774,5874	1,930055319
AUY57045.1	AUY59129.1	57	45	1,204729	458,5208	0,381153836	oppA3 Oligopeptide-binding protein OppA	46176,16	13126,61	16160,91	25915,86	34645,73	2077,95	0,623867934
AUY57042.1	AUY59126.1	44	30	1,803778	306,3095	0,600914392	oppD2 Oligopeptide transport ATP-binding protein OppD	65848,02	10351,89	18239,02	50892,83	111024,8	8429,3	0,830062091
AUY57265.1	AUY59346.1	18	15	3,199791	123,3614	0,658477964	oppF Oligopeptide transport ATP-binding protein OppF	407,5825	310,4324	242,6156	1569,527	1504,29	0	1,803778194
AUY55317.1	AUY57407.1	3	2	1,360282	28,9585	0,706135709	opuAC Glycine betaine-binding protein	5689,49	6650,129	6060,554	6038,964	5993,022	12997,44	3,199790908
AUY55273.1	AUY57363.1	25	19	2,570268	170,2669	0,410324269	opuBA Choline transport ATP-binding protein OpuBA	8449,753	7195,761	5661,862	5052,477	5123,499	44589,69	1,360282163
AUY56174.1	AUY58263.1	15	14	2,568283	127,5147	0,611406889	orn Oligoribonuclease	13400,19	4923,031	4500,621	4103,066	4783,745	0	2,570268034
AUY56057.1	AUY58148.1	7	4	1,799969	38,4238	0,322586967	otsA Trehalose-6-phosphate synthase	570,4992	901,6271	699,8737	651,6782	531,282	2726,572	0,38936521
AUY56089.1	AUY58180.1	6	3	2,310116	30,2425	0,577908734	pabC 4-amino-4-deoxychorismate lyase	3533,176	2286,848	1916,048	1569,401	1701,239	78,14177	1,799969006
AUY56577.1	AUY58666.1	8	3	32491,1	54,2405	0,235771552	pac2 Proteasome assembly chaperones 2 (PAC2)	0,042354	0,097301	0,971132	0,346808	0,977224	36089,37	0,432878735
AUY56767.1	AUY58855.1	11	8	1,656516	60,1543	0,343958904	pafA Pup--protein ligase	79876,12	79915,46	68121,59	67905,93	56709,91	12970,02	32491,09632
AUY56026.1	AUY58116.1	7	4	1,375681	35,9528	0,242955794	panC Pantothenate synthetase	8165,109	8973,35	12951,43	8280,809	13591,92	0	0,603676632
AUY57051.1	AUY59135.1	8	4	1,316308	52,7203	0,384699862	panE 2-dehydropantoate 2-reductase	3540,12	5274,62	6068,726	6383,4	4919,556	4,023979	0,726912951
AUY57303.1	AUY59384.1	15	9	10,29168	97,8186	0,402030933	parA Chromosome partitioning protein	10313,05	5787,198	10220,15	8008,599	48386,84	214485,7	0,759700737
AUY57302.1	AUY59383.1	42	27	21,321	313,7577	0,166348923	parB Chromosome partitioning protein ParB	239,4624	1051,832	652,4983	774,7776	883,71	39785,12	10,2916808
AUY55339.1	AUY57429.1	5	3	1,194835	26,3679	0,279550532	pat Phenylalanine aminotransferase	25526,25	35813,34	40145,79	56857,54	64396,98	3,767612	21,32099781
AUY57232.1	AUY59314.1	20	13	2,102143	143,8542	0,461712363	pbp1A Penicillin-binding protein	7254,948	10326,41	7409,911	12104,77	16387,77	24042,69	1,194835068
AUY56015.1	AUY58105.1	10	7	60,86159	75,2578	0,035212577	pbp4 D-alanyl-D-alanine carboxypeptidase	607,1843	961,9822	878,9148	1416,197	2589,721	144988,2	2,102143363
AUY55249.1	AUY57340.1	16	9	1,26589	91,2366	0,18801431	pbpA Penicillin-binding protein A	2074,667	1844,793	1716,769	1355,288	1691,149	4088,409	60,86159473
AUY55414.1	AUY57503.1	4	3	3,388243	18,927	0,746615286	pbpB1 Penicillin binding protein transpeptidase	944,7969	1460,856	1568,17	1906,881	3159,347	8398,051	1,265889949
AUY56499.1	AUY58587.1	5	5	1,686312	26,1354	0,085864865	pbpB2 Secreted penicillin-binding protein	64988,27	60589,97	48994,64	51856,73	40015,58	11651,16	3,3882432
AUY55696.1	AUY57787.1	60	39	1,75008	481,8743	0,213457371	pccB1 Propionyl-CoA carboxylase subunit beta	4995,547	2580,88	2743,87	2881,472	3015,572	0	0,593010039
AUY55697.1	AUY57788.1	24	17	1,80926	183,5441	0,359366227	pccB2 Propionyl-CoA carboxylase subunit beta	77667,93	120387,4	86965,52	77210,24	79817,67	506,6198	0,571402573
AUY55872.1	AUY57966.1	66	48	1,049089	586,9169	0,333241561	pckG Phosphoenol pyruvate carboxykinase	15819,59	11029,38	8406,165	15110,08	15239,68	6636,013	0,552712221
AUY57160.1	AUY59245.1	20	17	2,840749	101,2567	0,969044769	pcrA ATP-dependent DNA helicase	86294,08	42940,7	32142,69	28263,9	28443,55	100,6268	1,04908875
AUY55346.1	AUY57436.1	31	24	5,264749	290,6974	0,270473559	pdxS pyridoxal biosynthesis lyase	1022,555	1026,288	522,8234	200,1938	288,2752	0	0,352019868
AUY55347.1	AUY57437.1	2	1	1,298151	12,7824	0,188311275	pdxT Glutamine amidotransferase subunit	23871,8	22562,2	27725,61	57959,75	38109,28	201,3636	0,189942584
AUY56281.1	AUY58368.1	11	11	1,137292	93,1346	0,555457249	pdxY Pyridoxamine kinase	10946,24	20426,68	15351,96	11941,34	6764,104	34434,37	1,298151159
AUY56364.1	AUY58453.1	37	28	2,51078	319,1075	0,897234352	pepB Leucyl aminopeptidase	9331,734	10795,9	8105,208	5184,878	6059,079	0,691296	1,137291786
AUY56762.1	AUY58850.1	15	11	1,078402	101,8132	0,304757289	pepC2 Aminopeptidase 2	18008,2	16992,98	17084,47	15058,64	11234,84	29875,8	0,398282554
AUY57140.1	AUY59225.1	33	23	2,234983	206,554	0,970930142	pepD Serine protease	15430,38	17310,95	10429,59	9669,334	9613,63	33,03201	1,078402177
AUY56773.1	AUY58861.1	11	10	5,094416	86,1479	0,297786697	pepE Dipeptidase	8810,525	7758,077	4720,147	2325,905	1852,935	0	0,447430633
CpCAP3W_00312	CpCAPJ4_00310	4	2	2,490415	31,9634	0,226858223	pepG Aminopeptidase G	48132,37	29580,49	23129,53	14093,59	22877,87	3520,74	0,196293347
AUY55314.1	AUY57404.1	64	49	1,556114	515,1437	0,135043839	pepO Metalloendopeptidase	101589,6	104736,7	76250,23	82048,15	76169,55	23373,49	0,401539508
AUY56924.1	AUY59008.1	22	18	1,151178	194,7264	0,24439349	pfkA 6-phosphofructo kinase	26508,83	12695,87	21856,24	23149,67	47101,64	40,7073	0,642626586
AUY55465.1	AUY57554.1	12	9	1,114614	71,0085	0,49169813	pflA Pyruvate formate-lyase activating enzyme	131025	102909,5	101302,6	97681,85	98203,37	177774,9	1,15117809
AUY55467.1	AUY57556.1	87	67	1,172072	757,3351	0,748630319	pflB Formate acetyltransferase 1	101366,2	35328,88	38658,77	29893,36	38431,96	81284,85	1,114614196
AUY57163.1	AUY59248.1	60	48	2,313856	491,4392	0,785852315	pgi Glucose-6-phosphate isomerase	139044,2	67847,8	61415,98	61207,75	54747,27	2,049162	0,853190002
AUY56692.1	AUY58780.1	39	31	2,507341	401,2516	0,331488123	pgk Phosphoglycerate kinase	11708,67	14834,13	9183,233	6229,524	4203,282	79144,52	0,432179048
AUY56696.1	AUY58784.1	26	16	1,95383	200,7705	0,929773933	pgl 6-phosphogluconolactonase	812,2039	1269,618	985,4194	806,3204	760,3834	3,157182	2,507341074
AUY56119.1	AUY58208.1	7	4	6,09105	39,6861	0,320252023	pgm1 Phosphoglucomutase	1460,334	3203,522	2313,81	1445,015	1292,83	39763,47	0,511815271
AUY56858.1	AUY58944.1	21	18	2,048628	164,0152	0,605458638	pheS Phenylalanyl-tRNA synthetase subunit alpha	8905,585	7565,786	7447,563	13852,16	17952,44	17196,42	6,09105028
AUY56857.1	AUY58943.1	56	47	1,553115	360,2442	0,00192058	pheT Phenylalanyl-tRNA synthetase subunit beta	9507,157	17990,27	14986,98	12169,47	14980,09	204,7628	2,048628487
AUY56278.1	AUY58365.1	19	15	19,3834	117,2505	0,374073967	phoH Phosphate starvation-inducible protein PhoH	1054,908	599,976	1068,913	3290,859	1951,484	47554,12	0,643867212
CpCAP3W_00373	CpCAPJ4_00370	18	9	2,21177	108,9441	0,113087453	phoN Acid phosphatase	17458,43	16662,95	14315,52	9716,887	11974,09	208,631	19,38340467
AUY56066.1	AUY58157.1	15	10	2,331601	127,8947	0,263051851	phoP Two component system response transcriptional protein	1649,624	1396,603	2315,412	537,2387	296,7302	11667,24	0,452126527
AUY56067.1	AUY58158.1	6	4	1,506145	45,6261	0,77484603	phoR Two component system sensor histidine kinase protein	6590,468	31597,59	26192,22	21058,31	18479,06	3207,688	2,331601371
AUY56098.1	AUY58189.1	10	6	1,154383	51,4229	0,563942448	phoU Phosphate uptake regulator	14136,83	13418,75	20383,89	23029,98	18498,25	0	0,663946588
AUY56763.1	AUY58851.1	18	15	3,943238	120,8781	0,408743585	pimT SAM-dependent methyltransferase	15631,73	16443,49	14543,66	23363,61	19682,51	140783,2	0,866263722
AUY57108.1	AUY59193.1	27	19	2,727737	220,5627	0,205958107	pip Proline iminopeptidase	1642,96	1403,889	1254,648	713,197	863,7497	0	3,943237886
AUY55248.1	AUY57339.1	2	2	2,805968	12,3529	0,282012992	pknA Serine/threonine-protein kinase	6195,616	6193,257	5759,839	2797,704	3396,62	273,5732	0,366604212
AUY55247.1	AUY57338.1	19	12	3,006025	124,7683	0,141365141	pknB Serine/threonine-protein kinase	7037,813	3788,107	7122,266	11318,02	8135,669	34499	0,356383234
AUY56387.1	AUY58476.1	15	13	2,250985	80,3969	0,124481074	pknL Serine/threonine protein kinase	10415,51	19882,09	20371,26	31925,42	20830,13	61299,31	3,006024722
AUY55241.1	AUY57332.1	20	16	15,80926	178,0338	0,119654191	pld Phospholipase D	536,9148	516,3524	582,7366	939,6552	1919,282	23005,07	2,250985075
AUY56383.1	AUY58472.1	17	10	1,937872	113,8673	0,129184526	plsC 1-acyl-sn-glycerol-3-phosphate acyltransferase	4006,947	6097,162	5020,301	4296,093	3132,667	375,8886	15,8092611
AUY55838.1	AUY57932.1	3	2	6,678413	17,8377	0,241896947	plsC1 Acyltransferase family protein	18936,69	38264,95	32657,39	12122,48	1332,668	0	0,516029924
AUY55378.1	AUY57468.1	53	35	1,859875	420,4277	0,181433644	pmmB phosphoglucosamine mutase	6485,651	748,7927	1758,145	1777,422	3057,628	0	0,14973618
AUY56147.1	AUY58236.1	11	7	3,122541	63,8444	0,405441727	pncB Nicotinate phosphoribosyl transferase	91207,84	84017,07	66262,14	35651,08	38008,83	3676,796	0,537670525
AUY56531.1	AUY58619.1	99	76	2,137699	965,8466	0,117288448	pnp Polyribonucleotide nucleotidyltransferase	6175,028	6806,655	6549,195	5905,692	8001,062	27844,38	0,320251968
AUY56875.1	AUY58961.1	74	46	1,20807	523,8808	0,33421232	polA DNA polymerase I	36990,52	52119,13	44246,03	44885,38	42846,53	73371,03	2,137698535
AUY56014.1	AUY58104.1	19	13	2,355039	191,428	0,445662304	ppa Inorganic pyrophosphatase	154133,1	44706,86	41301,29	34219,22	66068,54	1681,338	1,208069583
AUY56694.1	AUY58782.1	101	69	3,170755	881,7501	0,299495968	ppc Phosphoenolpyruvate carboxylase	2604,636	3578,855	3536,765	2857,768	3188,358	24774,42	0,424621363
AUY56590.1	AUY58679.1	20	15	1,283883	156,6402	0,417524444	ppgk Polyphosphate glucokinase	124352,2	88753,56	87147,42	101424,9	101392,4	31046,1	3,170754992
AUY55234.1	AUY57325.1	17	14	1,106145	204,7756	0,421809513	ppiA Peptidyl-prolyl cis-trans isomerase A	12613,44	32893,8	45626,35	58030,94	38018,71	4757,307	0,778887262
AUY56632.1	AUY58720.1	13	11	1,919522	87,4301	0,831139265	ppiB Peptidyl-prolyl cis-trans isomerase B	21024,68	7879,68	9103,804	10555,51	9245,335	0	1,106144919
AUY56784.1	AUY58872.1	21	16	3,644846	145,9735	0,356779143	ppm1 Polyprenol-phosphate-mannose synthase domain 1	1029,752	401,2581	653,5884	456,1153	1194,492	5947,434	0,520962918
AUY56841.1	AUY58926.1	3	2	2,642682	15,1907	0,357338926	ppnk Inorganic polyphosphate/ATP-NAD kinase	10071,16	8279,314	10562,26	11131,78	22863,54	42411,85	3,644846495
AUY55863.1	AUY57957.1	75	56	1,266872	504,187	0,101094313	ppsA Phthiocerol synthesis polyketide synthase type I PpsA	2135,066	1967,876	1865,116	2241,673	2340,839	128,3492	2,642682377
AUY56870.1	AUY58956.1	7	4	13,79692	38,2632	0,442832087	pqqE Coenzyme PQQ synthesis protein E	4561,654	2987,596	2058,955	2747,647	2255,603	127560,4	0,789345806
AUY56966.1	AUY59050.1	43	36	1,448861	271,9478	0,450895819	prfA Peptide chain release factor 1	95498,18	52901,54	49826,4	36612,5	36536,46	63666,21	13,79691817
AUY55784.1	AUY57875.1	24	18	1,636575	170,8928	0,263497879	prfB Peptide chain release factor 2 (RF-2)	13856,17	16291,39	16266,68	25278,15	16100,62	34581,59	0,690197448
AUY57107.1	AUY59192.1	27	21	2,432897	198,5644	0,121283144	prfC Peptide chain release factor 3	49502,24	4611,418	5338,14	13218,03	11218,6	0	1,636574505
AUY56229.1	AUY58317.1	23	14	6,48236	160,4261	0,401848759	proA Gamma-glutamyl phosphate reductase	3005,952	1329,184	3836,783	4509,962	8786,557	39676,8	0,411032664

AUY56225.1	AUY58313.1	3	2	1,725634	21,4464	0,098211915	proB Glutamate 5-kinase	3590,5	2545,134	2693,875	2183,964	2916,304	16,40597	6,482360389
AUY55479.1	AUY57568.1	4	2	1,889512	20,6899	0,340456934	proC Pyrroline-5-carboxylate reductase	25981,53	7269,573	7541,48	12085,15	14369,85	50623,1	0,579497089
AUY56516.1	AUY58604.1	61	39	1,190801	456,8796	0,380172608	proS Prolyl-tRNA synthetase	18684,22	31219,2	25814,25	18497,56	35016,18	10071,76	1,889512477
AUY57099.1	AUY59184.1	32	26	1,722936	287,6211	0,513867896	prsA Ribose-phosphate pyrophosphokinase	31765,96	38426,89	33976,67	33970,37	29063,28	116443,8	0,83977091
AUY56543.1	AUY58631.1	34	26	1,892172	331,3685	0,485289459	pspA Phage shock protein A (IM30)	35437,9	30927,69	24246,63	19505,77	15531,28	136416,8	1,722936235
AUY57217.1	AUY59300.1	52	42	1,730394	464,1202	0,843366264	pspA1 phage shock protein A	896,6729	966,945	739,3712	744,9033	1263,894	2495,4	1,8921717
AUY56096.1	AUY58187.1	3	3	1,922944	14,2339	0,294197537	pstB Phosphate import ATP-binding protein	2015,894	1171,315	782,984	688,0215	773,3262	6173,11	1,730394269
AUY57102.1	AUY59187.1	17	9	1,334329	126,6185	0,81427176	pth1 Peptidyl-tRNA hydrolase 1	928,8194	1652,605	1521,076	1247,6	1090,244	736,7367	1,922943652
AUY57105.1	AUY59190.1	5	3	1,918398	26,4779	0,304157263	pth2 Peptidyl-tRNA hydrolase 2	13698,65	9955,076	7657,308	6198,241	10077,94	45,26568	0,749440549
AUY56076.1	AUY58167.1	21	17	3,289168	151,0794	0,319421725	ptrB Protease II	2582,651	2922,209	2111,555	1214,227	1101,378	0	0,521268258
AUY56563.1	AUY58651.1	8	5	1,149567	72,8352	0,263398001	ptsF PTS system fructose-specific transporter subunit IIABC	57328,01	64239,67	46110,6	31920,93	44015,57	116820,9	0,304028229
AUY56872.1	AUY58958.1	23	16	1,113698	212,6516	0,978858129	ptsG Phosphotransferase system II Component	82017,37	72765	97863,35	94954,18	186136,9	280,043	1,149567158
AUY56566.1	AUY58654.1	77	62	1,1226	578,9007	0,484250411	ptsI Phosphoenolpyruvate-protein phosphotransferase	13764,91	19333,57	22595,55	23922,65	36068,4	2531,057	1,113698434
AUY57100.1	AUY59185.1	6	5	1,726535	35,2017	0,707828833	pulA Pullulanase	9765,234	14685,42	10823,47	9755,824	14179,72	36966,47	1,122599635
AUY55960.1	AUY58051.1	20	13	1,219521	160,1776	0,392653746	purA Adenylo succinate synthetase	25147,5	22107,67	19299,62	18721,91	21152,94	14699,7	1,726534862
AUY56073.1	AUY58164.1	56	41	1,028861	424,5142	0,195909407	purB Adenylosuccinate lyase	28285,63	37430,49	40117,37	56814,48	45276,12	774,0784	0,81999425
AUY56074.1	AUY58165.1	30	25	1,24841	232,3566	0,506533399	purC Phosphoribosylaminoimidazole-succinocarboxamide synthase	37609,81	32203,13	27358,9	41791,06	33083,43	2962,006	0,971948227
AUY56070.1	AUY58161.1	37	30	1,484076	285,7259	0,458797062	purD Phosphoribosylamine--glycine ligase	4542,879	12068,01	11718,84	9431,635	9513,362	144,1343	0,801018975
AUY55702.1	AUY57793.1	6	4	1,432918	37,999	0,414581499	purE Phosphoribosyl amino imidazole carboxylase catalytic subunit	7473,969	6234,211	6304,758	6952,286	7012,065	2,211123	0,673819763
AUY56085.1	AUY58176.1	15	9	1,35471	108,8648	0,381971039	purF Amidophosphoribosyltransferase	12442,5	13224,97	9939,505	18646,98	7619,093	17,76996	0,697876659
AUY55701.1	AUY57792.1	16	10	1,679628	137,211	0,364548824	purK Phosphoribosyl amino imidazole carboxylase ATPase subunit	16738,79	31002,85	25471,3	20464,53	23124,25	0	0,595369826
AUY56080.1	AUY58171.1	16	10	2,44541	99,4173	0,310716041	purL Phosphoribosylformylglycinamidine synthase II	2572,399	5267,999	3750,812	2758,504	1981,482	0	0,408929343
AUY56086.1	AUY58177.1	29	21	8,061691	224,1768	0,329379831	purM Phosphoribosylaminoimidazole synthetase	21349,76	31392,76	32219,67	30578,09	32180,49	622180,3	8,061690525
AUY57157.1	AUY59242.1	17	12	1,431613	120,3177	0,509312541	purN Phosphoribosylglycinamide formyltransferase	11330,43	9481,218	16764,35	16665	28427,52	8701,773	1,431613484
AUY56079.1	AUY58170.1	16	11	1,289416	106,9067	0,4453045	purQ Phosphoribosylformylglycinamidne synthase subunit I	4701,392	6554,961	6746,813	7405,544	5946,088	610,6286	0,775544719
AUY56078.1	AUY58169.1	15	12	4257,036	128,8105	0,688035168	purS Phosphoribosylformylglycinamidine synthase subunit PurS	5,84942	10,06584	7,201341	0,649412	0,406281	98407,14	4257,035959
AUY55972.1	AUY58063.1	14	8	2,188112	82,224	0,321469163	purT Phosphoribosyl glycinamide formyltransferase 2	5887,018	4796,265	5163,475	4232,958	3009,247	0	0,457014897
AUY57115.1	AUY59201.1	23	16	1,126803	159,8433	0,966607239	pvdS Transcriptional regulatory protein PvdS	10341,93	16390	14583,07	10009,3	8274,321	28270,24	1,1268029
AUY55684.1	AUY57773.1	18	13	2,70392	130,1285	0,297625221	pyc Pyruvate carboxylase	7913,757	8007,292	6868,067	4659,636	3768,541	0	0,369833374
AUY56451.1	AUY58540.1	53	39	1,535352	527,5375	0,367593763	pyk Pyruvate kinase	168828,9	127977,9	109795,9	144313,1	117005,8	3508,247	0,651316555
AUY56666.1	AUY58754.1	3	3	1,854285	18,542	0,005143823	pyrB Aspartate carbamoyltransferase	10169,69	11197,86	9298,465	6426,872	5541,839	4569,209	0,539291316
AUY56667.1	AUY58755.1	8	7	3,155755	44,3686	0,050570225	pyrC Dihydroorotase	960,6555	1878,994	1586,696	3006,649	2945,22	8016,592	3,155754698
AUY56745.1	AUY58834.1	3	3	1,177173	14,6864	0,50196366	pyrD Dihydroorotate dehydrogenase 2	3812,97	329,1413	1275,37	2132,146	2469,963	0	0,849492571
AUY55947.1	AUY58038.1	7	6	1,574716	64,7026	0,892105971	pyrE Orotate phosphoribosyltransferase	7480,361	5287,348	5117,162	2159,877	2928,95	23074,77	1,574716405
AUY56670.1	AUY58758.1	3	3	10,35189	12,5107	0,512863474	pyrF Orotidine 5-phosphate decarboxylase	344,9338	286,5836	269,6625	264,1415	169,0625	8895,716	10,35189475
AUY56489.1	AUY58578.1	18	16	1,29935	151,3467	0,968196969	pyrH uridylate kinase	29864,13	40879,05	51975,52	70832,96	75869,41	12752,17	1,299349919
AUY56376.1	AUY58465.1	31	21	3,629168	224,6292	0,275432409	qcrA Ubiquinol-cytochrome c reductase iron-sulfur subunit	30958,84	29040,67	20893,3	12645,27	9644,366	0	0,275545241
AUY56377.1	AUY58466.1	12	6	1,697226	99,3543	0,666282533	qcrB Ubiquinol-cytochrome C reductase cytochrome B subunit	23085,34	2668,294	2128,401	1078,101	2059,355	13290,55	0,589196703
AUY55338.1	AUY57428.1	15	11	2,264951	106,4844	0,107315185	qorA Quinone oxidoreductase 1	2152,799	4673,129	5438,266	5334,707	8730,115	13712,98	2,264950769
AUY56522.1	AUY58610.1	18	16	1,852367	155,3702	0,112807446	rbfA Ribosome-binding factor A	23882,71	25186,92	19361,29	14055,37	17241,01	5645,836	0,539849798
AUY55232.1	AUY57323.1	11	9	6,417709	62,9304	0,579449389	rbsA Ribose import ATP-binding protein RbsA	1734,658	1064,411	471,3143	475,7806	840,3514	19672,24	6,417709189
AUY55693.1	AUY57784.1	14	9	1,541296	105,3977	0,011152392	rbsK Ribokinase	8500,487	9753,784	7088,892	5381,191	5329,201	5732,366	0,6488045
AUY55694.1	AUY57785.1	6	4	208,8075	35,3722	0,438214139	rbsR Ribose operon repressor	11,95413	11,39734	8,870132	20,50194	3,239439	6704,371	208,8075245
AUY56548.1	AUY58636.1	31	21	1,597298	255,7427	0,078547399	recA Recombinase A	9751,374	9923,152	10256,82	8214,703	6585,733	3938,299	0,626057224
AUY56760.1	AUY58848.1	4	4	8416,266	23,9953	0,547335852	recB2 Recombinase B	15,59727	0	0,763391	0	0,074974	137695,6	8416,265674
AUY55219.1	AUY57310.1	2	1	1,448294	10,3574	0,51622661	recF Recombination protein F	143,9321	1367,279	160,3396	626,227	527,9245	0	0,690467426
AUY56840.1	AUY58925.1	4	4	1,658955	26,1932	0,242715891	recN DNA repair protein	1200,758	1411,042	924,4502	938,5205	966,5534	226,5389	0,602789003
AUY55475.1	AUY57564.1	10	7	29,6455	47,9441	0,238001775	regX3 Sensory transduction protein	1550,136	699,5011	974,6332	1297,877	2413,908	91873,31	29,64549909
AUY56630.1	AUY58718.1	6	6	1,81698	24,6059	0,597799312	relA GTP pyrophosphokinase	1396,727	1308,02	1582,971	1059,769	1229,82	5501,106	1,816979674
AUY55335.1	AUY57425.1	11	5	3,442476	57,7014	0,85758666	rfbE O-antigen export system ATP-binding protein	1152,193	971,0896	797,0761	382,4743	459,0161	9211,776	3,442476462
AUY55746.1	AUY57837.1	15	11	1,225072	93,766	0,256824943	rhlE ATP-dependent RNA helicase	7023,238	7531,865	7224,531	7826,841	7394,408	11460,36	1,225071613
AUY56967.1	AUY59051.1	45	28	1,654141	339,9257	0,506048847	rho Transcription termination factor Rho	6918,449	29900,95	30565,91	21478,11	14797,34	4461,888	0,604543331
AUY56683.1	AUY58771.1	5	3	3,268349	44,2418	0,081418133	ribE Riboflavin synthase subunit alpha	1230,509	1498,473	1124,973	2599,501	2010,502	7986,069	3,268349033
AUY56528.1	AUY58616.1	20	11	2,049777	132,2014	0,33659617	ribF Riboflavin biosynthesis protein	9531,355	14619,45	11268,11	9192,368	8087,028	0	0,487857957
AUY56685.1	AUY58773.1	9	6	1,741743	61,4032	0,182915525	ribH 6,7-dimethyl-8-ribityllumazine synthase	3159,054	2005,835	983,9492	2350,269	4311,608	4047,817	1,741742791
AUY56529.1	AUY58617.1	13	8	1,963973	103,2004	0,33604408	rihA Pyrimidine-specific ribonucleoside hydrolase	17560,48	7303,383	5607,791	7357,007	7905,586	252,7199	0,509171942
AUY56474.1	AUY58563.1	7	5	2,213254	41,7328	0,163901398	rimM Ribosome maturation factor	2697,755	1309,116	1276,828	2260,681	2579,529	6853,959	2,213254317
CpCAP3W_01316	CpCAPJ4_01313	7	4	1,715609	35,3196	0,360827889	rlmN Ribosomal RNA large subunit methyltransferase N	7043,16	18653,71	14797,86	13570,35	9829,958	203,3961	0,58288349
AUY56828.1	AUY58913.1	5	2	2,724945	29,2033	0,036954077	rluB RNA pseudouridine synthase B	8222,556	7175,756	4549,244	3629,664	2363,743	1326,946	0,366979951
AUY55448.1	AUY57537.1	11	7	12,63681	65,3421	0,274611359	rmlD dTDP-4-dehydrorhamnose reductase	131,5349	733,8355	753,1273	607,9549	740,6886	19104	12,63680508
AUY56463.1	AUY58552.1	31	21	4,703231	222,4048	0,875435591	rnc Ribonuclease III	14814,79	2227,135	2049,549	1412,13	1191,343	87188,13	4,703231422
AUY56221.1	AUY58309.1	83	60	1,338103	620,0722	0,317791756	rne Ribonuclease E/G family	33479,41	118263,6	37369,38	79368,56	80389,59	93293,82	1,338103319
AUY56313.1	AUY58402.1	6	4	3,593106	39,6686	0,255028805	rnr Ribonuclease R	5437,768	4343,279	4269,241	1417,56	2492,786	0	0,278310718
AUY56681.1	AUY58769.1	3	3	1,75025	34,3392	0,58950882	rpe Ribulose-phosphate 3-epimerase	20808,99	29440,78	35379,47	87624,03	62186,45	62,05375	1,750249504
AUY57209.1	AUY59291.1	12	8	6,333505	83,9571	0,100939705	rpfA Resuscitation-promoting factor	2511,329	1361,54	1933,969	5313,043	3272,868	28191,73	6,333505438
AUY57121.1	AUY59207.1	33	23	2,881877	248,2086	0,167402053	rpfB Resuscitation-promoting factor RpfB	20532,72	9946,947	18203,26	29213,81	19133,56	91950,86	2,881877188
AUY56153.1	AUY58242.1	20	15	1,110613	140,2667	0,459542626	rph Ribonuclease PH	12986,09	16207,12	19440,97	19267,33	24386,78	136,275	0,900403689
AUY56203.1	AUY58291.1	17	11	1,923641	159,5126	0,403136668	rpiB Ribose-5-phosphate isomerase B	14379,81	23406,88	21203,63	18654,42	19558,85	75262,92	1,923640797
AUY55523.1	AUY57612.1	34	21	1,321997	316,9576	0,326514705	rplA 50S ribosomal protein L1	46636,82	60320,43	50996,62	57408,38	51823,07	99583,05	1,321996758
AUY55563.1	AUY57652.1	33	23	2,063812	293,5228	0,261693929	rplB 50S ribosomal protein L2	23682,68	28708,13	18878,3	17151,03	16632,05	749,6741	0,48454034
AUY55560.1	AUY57649.1	26	19	1,071442	237,128	0,560420954	rplC 50S ribosomal protein L3	51150,84	60145,6	43279,37	60224,72	57126,98	48267,31	1,071441941
AUY55561.1	AUY57650.1	22	17	1,129002	232,5618	0,894406937	rplD 50S ribosomal protein L4	71535,56	76784,62	44086,25	53707,15	43725,7	119794,3	1,129001545
AUY55578.1	AUY57666.1	18	17	2,056829	175,227	0,2941144	rplE 50S ribosomal protein L5	31941,02	71821,34	66992,43	47923,26	33947,93	1147,288	0,486185387
AUY55585.1	AUY57673.1	24	20	1,317863	244,6013	0,669869358	rplF 50S ribosomal protein L6	114558,4	40298,57	33750,27	51208,61	42229,07	155120,9	1,317863268
AUY57226.1	AUY59309.1	17	12	1,771709	167,3902	0,262850483	rplI 50S ribosomal protein L9	94246,62	103597,9	97603,66	84232,47	73700,07	8826,294	0,564426773
AUY55526.1	AUY57615.1	37	29	1,04497	322,7802	0,738855704	rplJ 50S ribosomal protein L10	44902,53	51590,55	43328,07	56937,76	47871,3	41299,91	1,044970489
AUY55522.1	AUY57611.1	18	15	1,147598	155,9087	0,546507962	rplK 50S ribosomal protein L11	82237,77	108242,2	97020,44	115671,8	111282,4	23569,45	0,871385446
AUY55527.1	AUY57616.1	23	18	1,31307	285,5746	0,245049181	rplL 50S ribosomal protein L7/L12	95727,58	105876,6	120503,2	109408,2	84429,04	51471,41	0,761574032
AUY55618.1	AUY57707.1	17	12	1,648543	170,5685	0,21943793	rplM 50S ribosomal protein L13	15057,56	25762,33	27142,27	35414,76	22146,77	54477,03	1,64854323
AUY55576.1	AUY57664.1	19	14	1,655678	184,8804	0,699770395	rplN 50S ribosomal protein L14	87374,62	119611,9	123027,1	263623,9	280307,5	2464,811	1,655678114
AUY55589.1	AUY57677.1	11	8	1,094493	100,5975	0,643686936	rplO 50S ribosomal protein L15	50609,57	55036,65	36373,65	37056,37	35136,93	57565,3	0,913665132
AUY55567.1	AUY57656.1	11	10	2,966576	119,2832	0,298515419	rplP 50S ribosomal protein L16	28622,56	35544,86	22397,64	15739,31	13440,82	0	0,337088967
AUY55608.1	AUY57697.1	14	9	2,33448	124,0297	0,212251292	rplQ 50S ribosomal protein L17	147261,3	158666,8	99835,22	92639,83	75734,89	5438,415	0,428360844
AUY55586.1	AUY57674.1	10	7	3,321195	85,5017	0,390749378	rplR 50S ribosomal protein L18	31729	38703,32	37130,02	37498,63	32481,14	287255,7	3,321195004
AUY56479.1	AUY58568.1	7	7	1,135643	65,2948	0,824118789	rplS 50S ribosomal protein L19	10902,43	10489,07	8791,589	9217,738	7498,521	17560,96	1,135643217
AUY56861.1	AUY58947.1	6	5	1,407666	45,1674	0,688903067	rplT 50S ribosomal protein L20	4259,378	10450,02	7075,012	5583,184	5457,391	19624,59	1,407665623
AUY56222.1	AUY58310.1	10	8	2,517272	85,0145	0,113216626	rplU 50S ribosomal protein L21	30560,56	30989,22	27506,81	14301,24	18263,03	2813,949	0,39725551
AUY55565.1	AUY57654.1	24	16	1,724216	211,9303	0,512786991	rplV 50S ribosomal protein L22	35502,12	32928,54	12338,97	24767,72	21661,93	92834,62	1,724215778
AUY55562.1	AUY57651.1	12	11	1,457499	120,4999	0,376634034	rplW 50S ribosomal protein L23	40253,82	49388,36	42906,88	51747,99	39194,84	0	0,686106975

AUY55577.1	AUY57665.1	12	9	2,03235	122,5007	0,186261405	rplX 50S ribosomal protein L24	40513,38	42527,8	27998,82	25503,45	24776,03	4356,778	0,492041278
AUY57101.1	AUY59186.1	11	8	1,136749	105,7461	0,737582072	rplY 50S ribosomal protein L25	42463,57	67719,25	75430,26	101121,1	101344,1	8530,367	1,136749431
AUY57143.1	AUY59228.1	4	2	1,220494	36,7853	0,320520491	rpmF 50S ribosomal protein L32	46615,82	32214,05	31892,36	41316,04	37367,06	56452,78	1,220494447
AUY57146.1	AUY59231.1	4	3	1,629803	49,7361	0,463830886	rpmG 50S ribosomal protein L33	80655,91	29447,57	21606,96	25890,94	27176,2	27746,6	0,613571265
AUY55607.1	AUY57696.1	49	36	1,215201	513,3845	0,442007792	rpoA DNA-directed RNA polymerase subunit alpha	67279,97	88161,42	61164,52	98018,16	79126,99	1101,82	0,822909048
AUY55535.1	AUY57624.1	120	86	1,599189	959,3362	0,361952242	rpoB DNA-directed RNA polymerase subunit beta	38552,85	50274,37	48799,68	38788,25	47202	70,19943	0,625317114
AUY55536.1	AUY57625.1	159	124	1,236331	1338,2447	0,637740953	rpoC DNA-directed RNA polymerase subunit beta	61854,16	151581,1	208604,5	298372	220643,2	2765,775	1,236331343
AUY56674.1	AUY58762.1	5	4	17,1837	37,9589	0,285089282	rpoZ DNA-directed RNA polymerase subunit omega	1545,511	2145,909	2146,667	3001,971	2204,264	95113,69	17,18369813
AUY56873.1	AUY58959.1	62	49	2,044844	724,158	0,160858044	rpsA 30S ribosomal protein S1	176539,3	101287,2	87214,9	83133,52	73462,99	21921,43	0,489034935
AUY56487.1	AUY58576.1	51	42	2,647966	425,8256	0,220501474	rpsB 30S ribosomal protein S2	99478,63	94754,09	109904,4	58778,46	54854,83	1223,594	0,377648387
AUY55566.1	AUY57655.1	32	27	2,212312	299,1764	0,332282607	rpsC 30S ribosomal protein S3	89650,99	95942,16	103710,8	76698,9	54071,08	0	0,452015848
AUY55606.1	AUY57695.1	33	16	1,796971	316,8595	0,064771899	rpsD 30S ribosomal protein S4	128551,5	94139,82	77293,77	69321,48	59877,99	37739,84	0,556492039
AUY55587.1	AUY57675.1	17	13	1,467077	162,4366	0,063529672	rpsE 30S ribosomal protein S5	42397,57	51403,45	56579,59	43060,32	31520,79	27922,47	0,681627606
AUY57228.1	AUY59311.1	12	10	3,641609	106,6258	0,424970856	rpsF 30S ribosomal protein S6	3354,044	7904,12	6118,893	5935,141	4555,963	52789,34	3,641608692
AUY55548.1	AUY57637.1	17	14	2,121581	156,7238	0,322169481	rpsG 30S ribosomal protein S7	55991,66	69173,82	50453,96	45862,6	36900,72	14,30573	0,471346628
AUY55584.1	AUY57672.1	27	21	1,023085	245,2526	0,755336014	rpsH 30S ribosomal protein S8	65030,02	76851,36	68293,8	68597,75	63085,72	73749,25	0,977435723
AUY55619.1	AUY57708.1	10	9	1,725923	118,6233	0,236320754	rpsI 30S ribosomal protein S9	18405,27	77586,75	84574,18	90448,92	84599,76	136594,6	1,72592271
AUY55605.1	AUY57694.1	6	4	1,074408	44,9506	0,916906329	rpsK 30S ribosomal protein S11	6039,123	10512,72	7213,974	6266,242	4248,176	15019,75	1,074407631
AUY55547.1	AUY57636.1	3	3	234,0326	15,8474	0,663419944	rpsL 30S ribosomal protein S12	149,5321	13,637	11,73172	10,61522	3,926923	40917,96	234,0326141
AUY55604.1	AUY57693.1	13	9	1,231205	146,846	0,47048428	rpsM 30S ribosomal protein S13	58824,47	59962,14	48260,2	65209,37	63839,55	6628,577	0,812212466
AUY56472.1	AUY58561.1	15	13	1,450051	167,5173	0,183112514	rpsP 30S ribosomal protein S16	89096,12	59225,16	72581,9	58995,57	62095,1	31250,94	0,689630688
AUY55569.1	AUY57658.1	10	8	1,445599	99,2029	0,224610386	rpsQ 30S ribosomal protein S17	27686,44	38724,16	33801,25	37050,84	37733,41	70081,86	1,445598607
AUY55564.1	AUY57653.1	12	12	1,166383	143,6974	0,438914176	rpsS 30S ribosomal protein S19	41535,91	51049,57	39204,14	32752,33	26492,29	53745,43	0,857351642
AUY57019.1	AUY59103.1	23	19	1,159683	190,3258	0,743457749	rrmA Ribosomal RNA methyltransferase	14285,44	12955,63	10577,59	10571,75	10878,84	22407,07	1,159683126
AUY56883.1	AUY58968.1	10	8	3,631005	57,6266	0,094233973	rsmD Ribosomal RNA small subunit methyltransferase D	11195,95	12691,44	14153,97	20716,67	24371,96	93039,74	3,631005318
AUY57124.1	AUY59210.1	10	4	1,249293	58,6546	0,291987013	rsmI Ribosomal RNA small subunit methyltransferase I	5728,231	5112,831	4454,128	6588,266	4667,181	7852,724	1,249292742
AUY56421.1	AUY58510.1	4	2	4,683769	29,0027	0,218395241	rulD Ribosomal large subunit pseudouridine synthase D	2760,75	3556,231	3234,809	1284,9	754,4388	0	0,213503265
AUY56623.1	AUY58711.1	4	3	1,699079	22,0085	0,381937925	ruvA Holliday junction DNA helicase subunit RuvA	1026,188	3470,155	2150,877	1936,911	1952,54	22,79763	0,588554077
AUY56984.1	AUY59068.1	13	7	1,424987	76,6051	0,42677922	sbcD Exonuclease, SbcD-family	5570,235	2296,69	2291,358	2914,49	4146,009	68,18579	0,701760813
AUY56732.1	AUY58821.1	63	47	6,523537	529,3586	0,352054914	sbm Methylmalonyl-CoA mutase large subunit	22748,36	21369,83	17729,29	21298,66	21907,75	360258	6,52353734
AUY57018.1	AUY59102.1	36	32	2,190609	260,8624	0,202303969	scrB Sucrose-6-phosphate hydrolase	9764,162	14579,52	10518,37	14235,76	14729,59	47403,78	2,190609026
AUY56640.1	AUY58728.1	13	8	1,436872	84,3165	0,387918133	sdaB L-serine dehydratase	26896,15	18619,02	18840,52	21323,04	23375,51	90,19393	0,695956282
AUY55458.1	AUY57547.1	83	64	1,425284	706,0968	0,404100736	sdhA Succinate dehydrogenase flavoprotein subunit	66795,4	72822,63	126385,9	84421,13	100861,5	1349,57	0,701614471
AUY55459.1	AUY57548.1	14	12	1,091735	134,6767	0,717227133	sdhB Succinate dehydrogenase iron-sulfur subunit	21669,52	17721,84	34121,56	39930,22	27075,12	18650,02	0,915973501
AUY55457.1	AUY57546.1	2	1	33,88006	11,9562	0,238296531	sdhC Succinate dehydrogenase cytochrome b556 subunit	77,90842	30,69082	3593,58	31,71492	77,5582	0	0,029515888
AUY55730.1	AUY57821.1	63	43	2,951202	432,204	0,276205356	secA Preprotein translocase subunit SecA	46569,33	15881,54	16290,88	8100,097	18564,9	16,248	0,338844974
AUY56626.1	AUY58714.1	30	16	3,014584	182,5519	0,095932204	secD Protein-export membrane protein	1836,448	20387,02	5239,617	26282,89	34722,87	21784,02	3,014583766
AUY55520.1	AUY57609.1	8	5	1,535987	46,2377	0,314803617	secE Preprotein translocase subunit SecE	3795,237	4459,002	4633,915	5873,425	10236,82	3685,791	1,53598658
AUY56627.1	AUY58715.1	11	8	4,751528	82,4946	0,015596752	secF Protein-export membrane protein	7702,694	5774,551	13274,87	2337,645	2354,191	938,3758	0,210458593
AUY56625.1	AUY58713.1	2	2	3,254354	22,2578	0,134223516	secN Preprotein translocase subunit YajC	7295,364	4600,69	3633,901	2428,332	2154,52	189,2019	0,307280623
AUY56411.1	AUY58500.1	7	7	1,021528	54,4613	0,397201724	sepF Cell division protein	2202,026	1542,95	1459,206	199,8622	343,4119	4551,233	0,978925528
AUY56909.1	AUY58994.1	34	23	15,39497	264,5591	0,04026159	serA D-3-phosphoglycerate dehydrogenase	80282,09	93352,06	81705,19	8442,41	7902,063	241,4175	0,064956267
AUY57199.1	AUY59281.1	11	6	1,142455	66,7423	0,533707122	serC Phosphoserine aminotransferase	6641,937	17306,66	12572,12	19817,13	21846,34	59,78005	1,142454541
AUY55837.1	AUY57931.1	33	28	1,754345	286,4958	0,355822977	serS Seryl-tRNA synthetase	40202,34	33495,07	31054,44	33751,5	25958,46	0	0,570013418
AUY55299.1	AUY57389.1	4	2	25,06052	25,2597	0,297401781	sgaA PTS system transporter subunit IIA	30,57787	35,60036	65,2164	83,03919	38,37484	3171,404	25,06052351
AUY56589.1	AUY58678.1	31	24	1,165427	204,1085	0,953174152	sigA RNA polymerase sigma-A factor	11150,43	10430,87	6581,093	4946,135	6682,007	21193,06	1,165427058
AUY56582.1	AUY58671.1	29	15	1,139506	194,3714	0,457864047	sigB RNA polymerase sigma-B factor	13630,71	41660,36	53679,93	55596,18	68576,87	0	1,139505522
AUY55633.1	AUY57722.1	3	1	10,77404	18,2932	0,076221405	sigD RNA polymerase sigma-D factor	290,9688	166,3004	104,8141	29,75338	22,41678	0	0,092815709
AUY55266.1	AUY57356.1	6	3	1,143716	30,5924	0,605308859	sigK RNA polymerase sigma-D factor	685,4672	730,2544	742,3884	233,1916	243,3499	1991,723	1,143715864
AUY55452.1	AUY57541.1	40	33	6,863268	444,9143	0,980397515	slpA Surface layer protein A	115891,7	175,1391	33,14036	20,60224	29,30874	796775,1	6,863268451
AUY55789.1	AUY57880.1	2	2	444,942	9,4291	0,474089102	smpB SsrA-binding protein/SmpB superfamily	4,20845	3,853196	0,50903	3,347786	0,259426	3809,847	444,9420486
AUY55819.1	AUY57910.1	10	8	1,854953	108,6066	0,351460645	sodA Manganese superoxide dismutase	47636,4	59664,99	67249,59	42670,42	51429,51	0	0,539097144
AUY57194.1	AUY59276.1	14	8	1,440558	109,1113	0,323381077	sodC Superoxide dismutase	38633,39	14932,78	17756,96	22553,02	32501,72	47690,34	1,440557769
AUY56835.1	AUY58920.1	6	3	7,796408	40,0235	0,187901295	soj Chromosome partitioning ATPase protein	504,5189	1210,081	1022,477	1648,398	1355,631	18335,34	7,796408462
AUY55658.1	AUY57747.1	8	7	1,245412	61,2719	0,281418813	spoU1 tRNA/rRNA methyltransferase SpoU	10436,11	8736,876	9088,834	7841,787	9820,082	5030,871	0,80294694
AUY57202.1	AUY59284.1	14	10	1,373263	99,6731	0,39035421	spoU2 tRNA/rRNA methyltransferase SpoU	2504,252	2287,437	3670,306	2784,363	3377,597	0	0,728192408
AUY55771.1	AUY57861.1	2	2	1,566326	10,1482	0,579700191	sprT Trypsin	183,8953	138,2587	125,197	356,8589	343,8389	0	1,566326145
AUY57041.1	AUY59125.1	6	6	1,280592	74,8377	0,973815408	sprX Trypsin-like serine protease	15161,72	12994,03	25033,9	15470,54	6484,888	46158,82	1,280591948
AUY55912.1	AUY58006.1	11	6	1,878874	66,0622	0,323494365	srtA1 Fimbrial associated sortase-like protein	12255,01	15283,78	14413,04	11213,14	11060,76	54,26495	0,532233597
AUY55937.1	AUY58028.1	3	3	56,33252	21,4961	0,381335974	srtA2 Sortase	4593,909	1801,137	1869,546	2534,371	2432,025	460599	56,33252497
AUY57227.1	AUY59310.1	9	7	5,176062	72,1472	0,326443754	ssb1 ssDNA-binding protein	4906,242	10218,4	7622,511	8576,961	7935,311	101228,4	5,176062056
AUY55945.1	AUY58036.1	29	23	1,924398	217,8316	0,346183559	sseB Thiosulfate sulfurtransferase	30268,23	26456,64	20941,23	20218,85	20139,79	0	0,51964297
AUY56707.1	AUY58795.1	33	25	9,062516	243,449	0,366642694	sufB FeS cluster assembly protein	5548,392	6537,269	5267,637	6067,355	5695,258	145501,9	9,062515744
AUY56709.1	AUY58797.1	26	19	2,587887	184,8905	0,265847598	sufC FeS assembly ATPase SufC	7753,962	16554,96	3157,157	13632,95	9089,989	48356,15	2,587886885
AUY56708.1	AUY58796.1	37	24	1,97765	244,2627	0,37325023	sufD FeS cluster assembly protein SufD	28563,28	11621,06	5133,51	11350,43	11564,57	0	0,505650741
AUY56706.1	AUY58794.1	2	1	1,788704	9,9683	8,41385173	sufR Iron-sulfur cluster biosynthesis transcriptional regulator SufR	397,0905	9,410921	413,7383	737,8339	729,3323	0	1,78870412
AUY56699.1	AUY58787.1	38	24	1,603952	295,7218	0,036890899	tal Transaldolase	58237,93	63565,05	85530,38	36113,03	50665,36	42485,65	0,623459926
AUY57122.1	AUY59208.1	12	9	5,082482	70,5966	0,347857195	tatD TatD family hydrolase	3961,564	3721,332	4380,644	3107,434	6037,375	52167,91	5,082481576
AUY55806.1	AUY57898.1	11	9	1,267529	75,957	0,32362274	tcsR Two-component system transcriptional regulatory protein	14963,84	13546,6	11100,17	9573,459	15083,96	6592,843	0,788936535
AUY57142.1	AUY59227.1	23	14	1,729344	206,1433	0,351266812	tcsR4 two component system response regulator	23898,04	18938,47	23061,32	18223,58	29122,48	66613,93	1,729343579
AUY56161.1	AUY58250.1	10	9	1,69492	75,891	0,345867009	tcsR5 Two component transcriptional regulator	10701,01	14208,48	11182,68	10274,88	10955,75	63,6884	0,589998244
AUY56619.1	AUY58707.1	10	6	2,6872	56,8621	0,067221364	tesB Acyl-CoA thioesterase II	5935,697	4576,622	3807,993	2404,527	2285,468	639,0888	0,372134579
AUY57073.1	AUY59158.1	5	2	5,114419	32,1551	0,166470527	tetR2 TetR family transcriptional regulator	513,0327	510,8913	427,6203	197,304	86,51008	0	0,195525643
AUY55352.1	AUY57442.1	14	8	1,136122	111,1896	0,007849669	tgt queuine tRNA-ribosyltransferase	12950,67	13491,69	12450,99	11348,51	11659,92	11225,01	0,880187482
AUY56971.1	AUY59055.1	43	29	1,002609	334,7676	0,902276407	thrA Homoserine dehydrogenase	12849,05	23198,88	20500,92	20506,49	16850,36	19044,85	0,997397711
AUY56970.1	AUY59054.1	16	14	1,489981	116,3621	0,346910532	thrB Homoserine kinase	13345,1	10711,69	12593,69	9963,322	13304,64	1329,998	0,671149586
AUY56323.1	AUY58412.1	52	39	1,49099	378,271	0,48540521	thrC Threonine synthase	32103,25	17698,61	23694,28	21634,31	23710,89	64236,86	1,490990468
AUY56610.1	AUY58698.1	58	36	2,023605	361,1908	0,292339426	thrS Threonyl-tRNA synthetase	14369,71	12160,53	13163,82	10982,68	8510,504	122,332	0,494167502
AUY56208.1	AUY58296.1	35	25	1,543131	331,0182	0,292667101	tig Trigger factor Tig	45145,26	67124,62	58621,64	70125,72	54649,29	138932,6	1,54313072
AUY56700.1	AUY58788.1	71	48	2,110122	606,6459	0,073206385	tkt transketolase	42115,09	55582,89	42460,17	28112,17	27998,48	10311,18	0,473906315
AUY56797.1	AUY58885.1	2	1	1,847558	15,5373	0,619276212	tlyC Hemolysin-related protein	1803,132	227,221	1167,327	2184,269	3723,629	0	1,847557682
AUY55724.1	AUY57815.1	5	2	4,540278	25,4272	0,273739214	tmk Thymidylate kinase	198,5319	358,791	362,68	351,2193	395,9029	3429,947	4,540278473
AUY55439.1	AUY57528.1	36	21	1,853896	225,3265	0,705763827	topA DNA topoisomerase I	6425,965	3886,137	4787,759	2792,687	4404,442	20796,44	1,853895964
AUY56002.1	AUY58093.1	37	26	3,721231	339,4297	0,74518029	tpdA Thiol precursor dipeptidase	23928,63	32143,18	28938,71	13652,77	16046,92	286644,1	3,721231187
AUY56693.1	AUY58781.1	36	29	1,530551	372,4873	0,372602907	tpiA Triosephosphate isomerase	61141,36	84854,08	70900,07	68118,27	73590,56	1,916079	0,653359511
AUY56633.1	AUY58721.1	18	11	1,629395	194,3891	0,360503747	tpx Thiol peroxidase	59912,86	86924,57	92563,59	77503,29	69233,96	189,0964	0,613724803
AUY56072.1	AUY58163.1	2	2	1,260415	18,6912	0,399694463	trkA2 Trk system potassium uptake protein	8697	5207,431	6591,301	9381,914	6879,191	0	0,793389781
AUY55871.1	AUY57965.1	10	8	2,421939	63,4772	0,002421462	trmB tRNA (guanine-N (7))-methyltransferase	4500,005	3470,262	3590,159	7684,5	9460,906	10853,24	2,421938935
AUY55333.1	AUY57423.1	29	19	1,772195	222,6708	0,06827219	troA Periplasmic zinc-binding protein	9304,872	18242,71	14900,46	22126,94	21110,77	31988,51	1,772195218

AUY57285.1	AUY59366.1	4	2	1,373326	35,349	0,392627392	trpA Tryptophan synthase subunit alpha	1273,478	1102,227	1846,019	1651,182	1422,905	0	0,728159
AUY56449.1	AUY58538.1	16	12	11,80635	104,1404	0,386426349	trpC2 Indole-3-glycerol phosphate synthase	2535,776	1327,85	1401,318	2004,034	1370,529	58785,23	11,80635372
AUY57279.1	AUY59360.1	7	5	5,549968	32,3051	0,937910064	trpE Anthranilate synthase	5144,059	1111,172	176,5917	315,2468	148,3264	35232,84	5,549968193
AUY57280.1	AUY59361.1	2	2	12,18959	17,2581	0,067825491	trpG Anthranilate synthase component II	19833,64	3247,663	12020,42	56585,72	31735,12	339554,6	12,18958573
AUY55673.1	AUY57762.1	31	22	2,12992	241,3851	0,592919738	trpS Tryptophanyl-tRNA synthetase	18664,82	20952,27	18976,09	15704,57	14521,97	94572,26	2,129920088
AUY56948.1	AUY59032.1	16	11	1,233184	118,2029	0,345677695	trxA1 Thioredoxin TrxA	4809,279	6099,485	5697,927	8255,267	7384,282	4839,563	1,233184385
AUY57219.1	AUY59302.1	11	10	1,950456	110,3718	0,262239306	trxA2 Thioredoxin TrxA	52760,54	35634,74	55462,57	67420,04	167405,8	45762,62	1,950456392
AUY57299.1	AUY59380.1	9	7	1,185015	76,9116	0,471873896	trxA3 Thioredoxin	18352,69	12625,26	13399,67	10221,96	8001,637	19225,39	0,843871359
AUY57298.1	AUY59379.1	9	6	1,076979	82,7508	0,59383217	trxB Thioredoxin reductase	10592,28	11061,38	9313,567	12289,19	9367,98	7096,632	0,928523409
AUY56488.1	AUY58577.1	50	37	1,222748	465,3423	0,824393682	tsf elongation factor Ts	71259,25	61260,24	37766,76	37781,09	47450,64	122985,4	1,222747619
AUY56859.1	AUY58945.1	4	1	2,869681	23,9912	0,701254633	tsnR rRNA methyltransferase	843,8655	937,3929	188,1831	2743,987	2907,682	0	2,8696809
AUY55550.1	AUY57639.1	63	47	1,166836	721,4627	0,878832584	tuf Elongation factor Tu	273123,7	362197,9	305335,2	294982,8	199863,6	602746,3	1,166836402
AUY57037.1	AUY59121.1	47	31	4,130822	372,7394	0,678274884	typA GTP-binding protein TypA/BipA	9216,841	14438,22	12668,3	10419,09	4710,009	134916,2	4,130821579
AUY56847.1	AUY58933.1	14	9	3,079664	88,179	0,531867523	tyrS Tyrosyl-tRNA synthetase	6949,869	1169,969	966,389	1594,273	1863,788	24524,47	3,079664198
AUY55516.1	AUY57605.1	9	7	1,357173	55,4678	0,364455319	ubiE Ubiquinone/menaquinone biosynthesis methyltransferase	2514,989	3514,053	3520,339	2905,249	3705,248	6349,669	1,357173343
AUY56887.1	AUY58972.1	11	3	3,146232	67,2226	0,271707678	ung Uracil-DNA glycosylase	2422,944	3287,908	3377,84	1411,181	1477,574	0	0,317840468
AUY55679.1	AUY57768.1	18	17	1,923064	165,7275	0,250338139	upp Uracil phosphoribosyltransferase	28098,74	21854,63	21800,44	26177,11	28564,22	83245,85	1,923064109
AUY57078.1	AUY59163.1	2	2	1,923772	11,0206	0,657546433	uppS1 Undecaprenyl pyrophosphate synthase 1	524,0979	1920,302	721,1096	1932,925	4153,457	3,33517	1,923771601
AUY55893.1	AUY57986.1	6	4	2,912402	34,8698	0,214381225	ureA Urease subunit gamma	1177,794	944,9418	1126,518	582,6716	524,7689	8,220613	0,343359189
AUY55894.1	AUY57987.1	6	5	4,886135	33,6599	0,310983795	ureB Urease beta subunit	509,9591	2111,602	75,16278	844,3404	787,7005	11544,51	4,886134812
AUY55895.1	AUY57988.1	12	8	1,004473	65,2407	0,999373377	ureC Urease alpha subunit	6388,067	3673,206	4603,286	4907,163	3637,534	6054,563	0,995547171
AUY55896.1	AUY57989.1	16	14	1,304522	114,9832	0,868080016	ureE Urease accessory protein UreE	8243,774	8626,328	8109,518	4133,565	4215,129	24237,78	1,30452249
AUY55898.1	AUY57991.1	20	16	6,430802	145,6363	0,587831003	ureG Urease accessory protein UreG	2146,62	3148,912	2843,291	2239,203	1302,678	48797,28	6,43080202
AUY55797.1	AUY57889.1	33	24	1,277773	271,8856	0,392457791	uspA Universal stress protein A	39927,05	40374,68	33706,52	37716,88	37970,83	69988,91	1,277772547
AUY56868.1	AUY58954.1	25	11	1,093872	190,4007	0,513932281	uspA3 Stress related protein	12456,87	18586,51	17765,78	19441,51	24375,66	803,3777	0,914183669
AUY57235.1	AUY59317.1	6	5	1,030355	35,7771	0,547310704	uspA4 Universal stress protein UspA	3206,327	4349,208	4235,345	5502,301	6510,008	136,4865	1,030355242
AUY56864.1	AUY58950.1	10	7	1,89215	50,9588	0,359297327	uvrA1 UvrABC system protein A	58278,68	26885,47	21357,8	30772,2	25524,56	0	0,528499203
AUY56869.1	AUY58955.1	5	3	5,277281	22,3267	0,208208801	uvrB2 UvrABC system protein B	531,3605	1907,495	2767,179	1898,262	3627,999	21947,45	5,277280577
AUY55754.1	AUY57845.1	8	7	2,064957	45,4181	0,321907713	uvrD3 DNA helicase II	1995,16	4846,069	4016,438	2830,376	2416,559	11,12468	0,484271564
AUY56215.1	AUY58498.1	79	60	1,030375	569,9353	0,468883781	valS Valyl-tRNA synthetase	33178,68	20891,08	25156,61	36034,44	40750,99	105,3457	0,970520033
AUY56759.1	AUY58847.1	5	4	1,133808	44,2089	0,672367021	vapI Virulence-associated protein I	6985,253	8124,556	8166,631	14787,65	10762,1	841,2759	1,13380841
AUY56690.1	AUY58778.1	27	20	5,360294	161,56	0,888759281	whiA Sporulation transcription regulator	1998,729	1791,67	2409,899	423,3037	321,2792	32490,84	5,36029355
AUY56836.1	AUY58921.1	2	2	1,986368	17,1864	0,343379283	xerD tyrosine recombinase XerD	4571,586	8801,348	10269,13	5068,855	6833,004	0,297922	0,503431359
AUY55985.1	AUY58076.1	24	17	1,278266	146,1221	0,550866144	xthA Exodeoxyribonuclease III	20450,93	9734,233	9019,926	7517,753	9157,5	13995,28	0,782309836
AUY55417.1	AUY57506.1	10	8	2,748947	81,2145	0,619333196	yabJ RutC family protein	1183,474	3718,713	5153,33	1857,694	2315,685	23468,7	2,748946825
AUY55362.1	AUY57452.1	8	5	1,427724	90,3192	0,333076568	ybaB Hypothetical protein	130628,7	83716,44	139448,5	135895,5	79000,6	32906,35	0,700415246
AUY55739.1	AUY57830.1	4	2	2,618716	19,8421	0,272685801	ybaK Cys-tRNA(Pro)/Cys-tRNA(Cys) deacylase	168,4801	305,7623	330,4997	183,4967	123,8074	0	0,381866528
AUY56652.1	AUY58740.1	2	1	48,87037	11,0343	0,059103425	yceG Amino deoxychorismate lyase	1019,538	824,8702	7042,244	137,5522	44,28913	0	0,020462298
AUY56787.1	AUY58875.1	14	8	1,165039	80,1591	0,430478864	yceI1 Protein yceI	59137,55	14084,4	17139,25	29262,12	48298,57	0	0,858340618
AUY57058.1	AUY59143.1	46	29	1,310284	327,2159	0,425941603	ychF GTP-binding protein YchF	100444,9	90731,68	85551,23	99137,56	106466,3	5592,97	0,76319328
AUY56921.1	AUY59006.1	22	21	1,112126	167,3728	0,734317326	yghZ Aldo-keto reductase	24485,69	26593,93	19764,19	21461,34	20416,91	36908,99	1,112125802
AUY57305.1	AUY59386.1	6	5	1,191857	41,3677	0,668553173	yidC1 Inner membrane protein translocase component YidC	692,763	5311,826	6525,766	3804,531	2210,038	8919,821	1,191856893
AUY56998.1	AUY59082.1	6	4	6,539747	49,8081	0,69540692	yihR Aldose 1-epimerase	1108,371	1484,876	1146,445	452,267	845,7979	23158,57	6,539747115
AUY55412.1	AUY57501.1	6	5	1,414824	36,3693	0,441850675	ykuE Metallophosphoesterase	1709,85	4074,419	2112,399	2603,062	2766,312	212,0049	0,706801857
AUY55413.1	AUY57502.1	12	9	1,306578	79,4263	0,868687645	yqeY Hypothetical protein	3496,992	12090,53	16882,05	7176,659	9979,663	7694,537	0,765358202
AUY55304.1	AUY57394.1	2	2	2,158639	9,8103	0,205793343	yusR Short-chain dehydrogenase/reductase	68,7854	620,6017	791,3358	1287,465	930,7103	978,1696	2,158638509
AUY56112.1	AUY58201.1	15	10	1,100729	109,7907	0,452142607	yvqK Cob(I)yrinic acid a,c-diamide adenosyltransferase	10253,65	11746,61	9530,856	10878,39	10131,03	13697,81	1,100729246
AUY55843.1	AUY57937.1	16	12	5,450478	115,9668	0,221081892	yvrC ABC transporter substrate-binding protein	367,3068	2770,374	2331,435	2694,534	2591,424	24523,34	5,450477888
AUY56964.1	AUY59048.1	9	6	1,726789	63,6508	0,000851	ywlC tRNA threonylcarbamoyladenosine biosynthesis protein YwlC	4477,625	4673,114	4506,059	7200,37	7606,383	8775,653	1,726788827
AUY57069.1	AUY59154.1	7	5	1,830747	56,8165	0,378862904	ywnH Phosphinothricin acetyltransferase YwnH	2912,302	10427,35	2756,929	4582,605	4209,75	0	0,546224988
AUY56698.1	AUY58786.1	36	28	2,341064	271,0882	0,023239773	zwf Glucose-6-phosphate 1-dehydrogenase	42247,08	33264,74	21287,63	15534,6	14940,83	10873,05	0,42715617
