# Supplementary material for: Comparative Proteomic Analyses Between Biofilm-Forming and Non-biofilm-Forming Strains of Corynebacterium pseudotuberculosis Isolated From Goats
Source: Front Vet Sci. 2021 Feb 16;8:614011. doi: 10.3389/fvets.2021.614011 (PMC7921313; doi:10.3389/fvets.2021.614011)
Supplement: Supplementary File 2 — Proteins identified exclusively in the proteomes of CAP3W or CAPJ4. [file Data_Sheet_2.PDF]

Supplementary file 2: Proteins identified exclusively in the proteomes of CAP3W or CAPJ4.

Accession CAP3W	Score	Description	Biological Process
AUY55707.1	9,9903	Hypothetical protein	Function unknown
AUY55849.1	10,768	LPxTG domain-containing protein	Cell wall/Membrane and Envelope biogenesis
AUY56631.1	12,9822	Hypothetical protein	Function unknown
AUY56511.1	43,6007	Methylated DNA-protein cysteine methyltransferase	DNA Metabolism: replication, recombination and repair
**Accession CAPJ4**			
AUY58300.1	25,6214	ATP-dependent Clp protease ATP-binding subunit	Post-translational modification, protein turnover, chaperones
AUY58488.1	96,1091	Penicillin-binding protein	Cell wall/Membrane and Envelope biogenesis
AUY58697.1	32,5608	Dyp-type peroxidase family protein	Post-translational modification, protein turnover, chaperones
