# Supplementary figures and images for: Comparative Proteomic Analyses Between Biofilm-Forming and Non-biofilm-Forming Strains of Corynebacterium pseudotuberculosis Isolated From Goats
Source: Front Vet Sci. 2021 Feb 16;8:614011. doi: 10.3389/fvets.2021.614011 (PMC7921313; doi:10.3389/fvets.2021.614011)

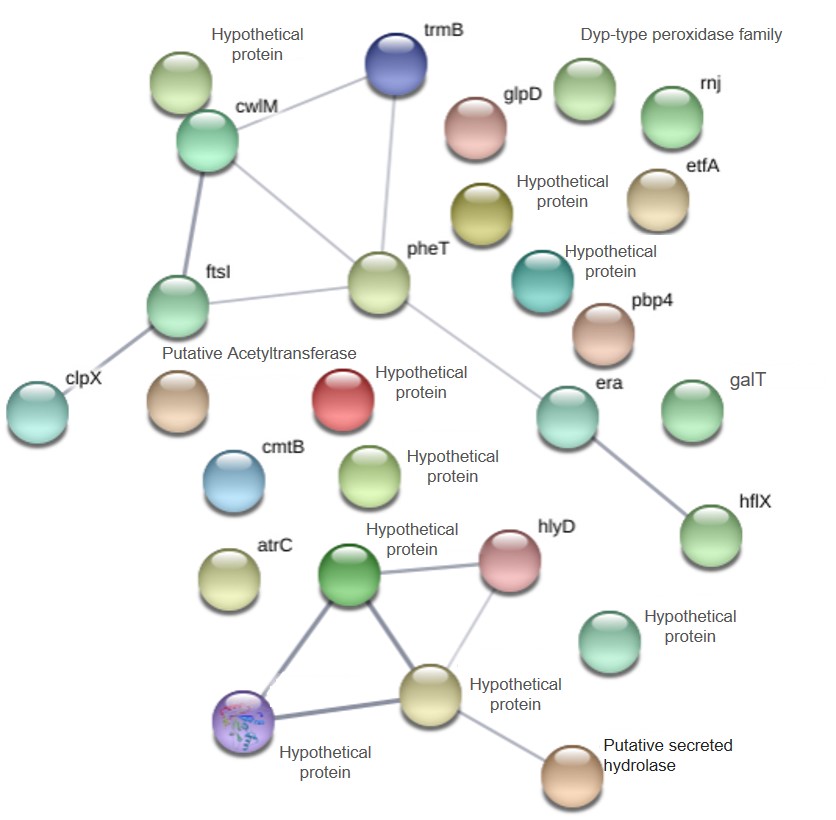

Supplement: Supplementary Figure 1 — STRING protein-protein network analysis of additional induced and exclusive proteins detected in the CAPJ4 strain. The thickness of the line indicates the degree of confidence prediction of the interaction. All proteins had 100% identity and a median confidence score ≥ 0.400. [file Image_1.JPEG]
